# Range-wide whole-genome resequencing of the brown bear reveals drivers of intraspecies divergence

**DOI:** 10.1038/s42003-023-04514-w

**Published:** 2023-02-06

**Authors:** Menno J. de Jong, Aidin Niamir, Magnus Wolf, Andrew C. Kitchener, Nicolas Lecomte, Ivan V. Seryodkin, Steven R. Fain, Snorre B. Hagen, Urmas Saarma, Axel Janke

**Affiliations:** 1 Senckenberg Biodiversity and Climate Research Institute (SBiK-F), Georg-Voigt-Strasse 14-16, Frankfurt am Main, 60325 Germany; 2 Institute for Ecology, Evolution and Diversity, Goethe University, Max-von-Laue-Strasse. 9, Frankfurt am Main, Germany; 3 Department of Natural Sciences, National Museums Scotland, Chambers Street, Edinburgh, EH1 1JF UK; 4 School of Geosciences, University of Edinburgh, Drummond Street, Edinburgh, EH8 9XP UK; 5 Canada Research Chair in Polar and Boreal Ecology, Department of Biology, University of Moncton, Moncton, New Brunswick E1H1R2 Canada; 6 Pacific Geographical Institute of the Far Eastern Branch of the Russian Academy of Sciences, 7 Radio St., Vladivostok, 690041 Russia; 7 National Fish & Wildlife Forensic Laboratory, Ashland, OR USA; 8 Norwegian Institute of Bioeconomy Research, Division of Environment and Natural Resources, Svanhovd, N-9925 Svanvik, Norway; 9 Department of Zoology, Institute of Ecology and Earth Sciences, University of Tartu, J. Liivi 2, Tartu, 50409 Estonia; 10 LOEWE-Centre for Translational Biodiversity Genomics (TBG), Senckenberg Nature Research Society, Georg-Voigt-Strasse 14-16, Frankfurt am Main, Germany

**Keywords:** Evolutionary genetics, Comparative genomics

## Abstract

Population-genomic studies can shed new light on the effect of past demographic processes on contemporary population structure. We reassessed phylogeographical patterns of a classic model species of postglacial recolonisation, the brown bear (*Ursus arctos*), using a range-wide resequencing dataset of 128 nuclear genomes. In sharp contrast to the erratic geographical distribution of mtDNA and Y-chromosomal haplotypes, autosomal and X-chromosomal multi-locus datasets indicate that brown bear population structure is largely explained by recent population connectivity. Multispecies coalescent based analyses reveal cases where mtDNA haplotype sharing between distant populations, such as between Iberian and southern Scandinavian bears, likely results from incomplete lineage sorting, not from ancestral population structure (i.e., postglacial recolonisation). However, we also argue, using forward-in-time simulations, that gene flow and recombination can rapidly erase genomic evidence of former population structure (such as an ancestral population in Beringia), while this signal is retained by Y-chromosomal and mtDNA, albeit likely distorted. We further suggest that if gene flow is male-mediated, the information loss proceeds faster in autosomes than in X chromosomes. Our findings emphasise that contemporary autosomal genetic structure may reflect recent population dynamics rather than postglacial recolonisation routes, which could contribute to mtDNA and Y-chromosomal discordances.

## Abstract (web summary)

The genetic structure of modern-day brown bears mainly reflects contemporary population connectivity. Recent gene flow barriers have obscured the genomic signatures of postglacial recolonisation.

## Introduction

While inferring present-day population structure from genetic data is relatively straightforward, it remains challenging to unearth the true underlying demographic processes out of the myriad of potential alternative scenarios. Population-genomics studies can complement mitochondrial-DNA (mtDNA) phylogeographical inferences^1–3^, and provide new insights into the long-standing question of how species differentiate over time in different parts of their geographical ranges. We present the first comprehensive population-genomics study of the brown bear (*Ursus arctos*), a text book example species of the effect of Quaternary glaciation cycles on present-day intraspecies diversity and divergence^4^.

Brown bears have a broad Palaearctic distribution^5^ and exhibit extensive morphological, ecological, and behavioural variability across a large spatial scale^6–10^. While numerous nuclear DNA (nDNA) studies have elucidated the evolutionary relationship between brown bears and polar bears (*Ursus maritimus*)^11–17^, brown bear phylogeography still relies mostly, with few exceptions^17–19^, on analyses of mtDNA data^20–31^. These analyses revealed dissimilarity across contiguous populations and haplotype sharing across populations separated in time and space^23^, resulting in strikingly disjunct distributions of mtDNA haplotypes. Complex and contested postglacial recolonisation models have been proposed to account for these cryptic patterns^20,24^.

For example, mtDNA haplotype 1a is present in Iberia and southern Scandinavia, but is very rare in other parts of Europe^32,33^. This disjunct distribution is considered a vestige of the recolonisation of southern Scandinavia from an Iberian glacial refuge^4,21,34,35^. However, this hypothesis is inconsistent with the reported lack of mtDNA phylogeographical structure in Europe during and following the Last Glacial Maximum (LGM: 25-18 kya)^27,32,36,37^. Another highly debated aspect of brown bear phylogeography concerns the ABC-Islands brown bears, in the Alexander Archipelago in southeastern Alaska. These bears carry mtDNA haplotypes of clade 2, most similar to those of polar bears^30^. This observation has been attributed to recent (post-LGM) hybridisation between polar bears and ABC-Islands brown bears^15^, but this purported introgression event does not in itself explain the co-occurrence of haplotype 2 variants in extinct Holocene Irish brown bears^24^.

The competing or complementary explanation, according to which brown bear mtDNA structure reflects stochastic single-locus dynamics rather than the true evolutionary relationships between populations^3,38^, can only be tested using a dataset of multiple independently segregating loci^39^. Existing brown bear nDNA studies either span a fraction of the entire species range or are based on a limited number of markers^13,14,17–19,33,40^, and hence do not suffice.

To fill this gap, we compiled a whole-genome dataset of 128 brown bear individuals spanning the entire species range, of which 95 genomes were generated for this study. We employed genetic markers from genomic regions with four different inheritance properties (autosome, X chromosome, Y chromosome and mitogenome) to obtain a comprehensive overview of brown bear population structure and genetic diversity. We found that, unlike mtDNA and Y-chromosomal phylogeographical patterns, brown bear autosomal population structure is largely explained by recent population connectivity, with the notable exception of an apparent genomic discontinuity in western Siberia. We argue, using SLIM2 forward-in-time simulations and a novel approach of multispecies coalescent (MSC) based analyses applied to haploblocks, that the discrepancies between genetic markers either reflect differences in temporal resolution or single-locus stochastics. We also present evidence that postglacial recolonisation created intraspecific hybridisation zones in Alaska and eastern Siberia, and that North American and Hokkaido brown bears could preserve introgressed polar bear DNA owing to their isolation from the Eurasian mainland.

## Results

### Autosomal population structure

**Fig. 1:**
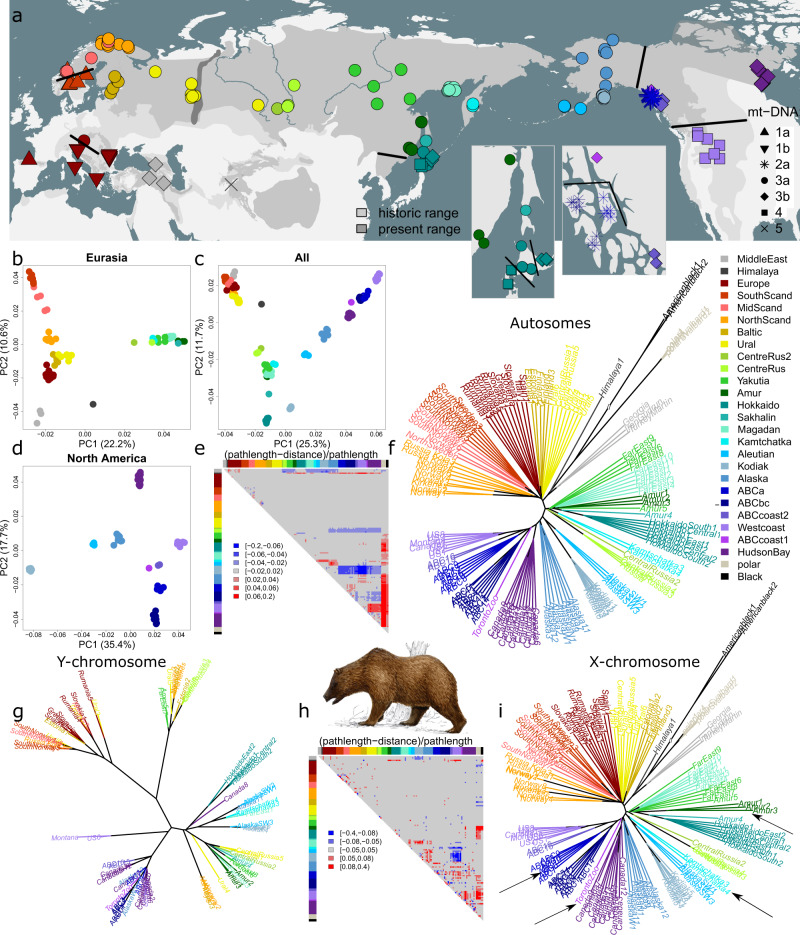
Brown bear genetic structure: unlike single-locus (mtDNA and Y chromosome) clustering, multi-locus (autosomes and X chromosomes) clustering is largely explained by present-day population connectivity, except for a genomic discontinuity in western Siberia. **a** Sample distribution. Dark and light grey: present-day and historical geographical range of brown bears (https://www.iucn.redlist.org). Symbol types indicate mtDNA haplotypes. Black lines indicate mtDNA discontinuities. The Ural Mountains (darkgrey shape) and the rivers Ob, Yenisei and Lena (from west to east) are depicted for reference. **b**–**d** PCoA-scatterplots based on allele sharing distances calculated from autosomal SNP data. **e** Residual heatmap for the dendrogram in f, depicting the difference between path lengths in the dendrogram relative to the actual genetic distances in the underlying distance matrix. Red (attraction) indicates that samples are more similar than suggested by the dendrogram, whereas blue (repulsion) indicates the opposite. **f** Unrooted ‘biological neighbour-joining’ (bioNJ) dendrogram (note: not evolutionary phylogeny) based on autosomal allele sharing distances. **g** Unrooted Y-chromosomal maximum-likelihood phylogeny (generated with the software RAXML), excluding the distant outgroup samples from the Middle East and Himalayas. **h** Residual heatmap for the dendrogram in i. **i** Unrooted bioNJ dendrogram depicting X-chromosomal Euclidean genetic distances. Arrows indicate samples or populations which cluster differently or have different branch lengths relative to the autosomal bioNJ dendrogram in f.

**Fig. 2:**
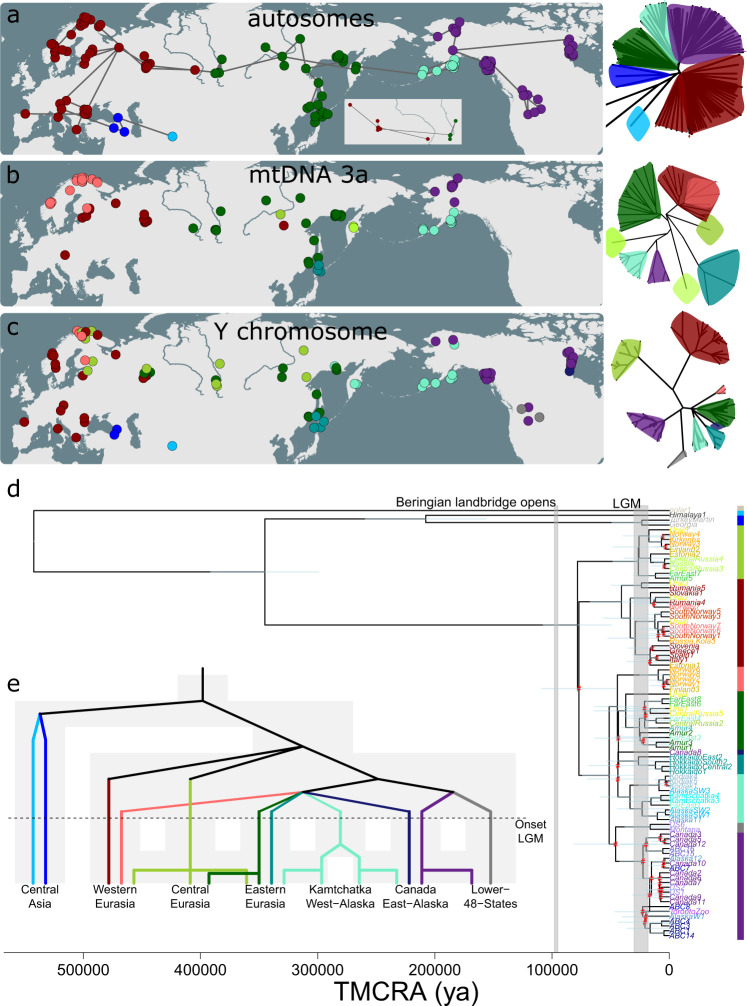
Spatial distribution of genetic clusters, depicting the (in)consistencies between markers, possibly partly caused by incomplete lineage sorting. Geographical distribution of individuals, with colour coding according to genetic cluster instead of population assignment. **a** Geographical map showing hierarchical clustering (using bioNJ method) of brown bear individuals based on Euclidean distances for autosomal SNP data. Lines connecting samples represent a minimum spanning tree (generated with the function ‘mst’ of the R-package ‘ape’), based on allele sharing distances. Inset: detail of minimum spanning tree in central Russia, which highlights that the transition in western Siberia between the western (red) and eastern (darkgreen) Eurasian clades is abrupt rather than gradual. **b** Geographical map showing the distribution of variants of mtDNA haplotype clade 3a, according to a maximum likelihood phylogeny generated with the software IQtree. **c** Geographical map showing the distribution of Y chromosome haplotypes (see d). **d** Rooted Y-chromosomal maximum-likelihood phylogeny, generated with the software IQtree. The phylogeny has been linearised using the mean path length method, and branch lengths have been converted in TMCRA-estimates assuming a mutation rate of 1.3 × 10^−9^ per site per year. Lightblue bars indicate confidence intervals of the node ages. Nodes with ultrafast bootstrap support values below 0.95 are highlighted in red. The colour bar on the righthand side corresponds to the genetic clusters in Fig. 2c. The tip colour coding (sample names) corresponds to population assignment as in Fig. 1. **e** Speculative model on Y-chromosomal phylogeography, with background grey shaded areas depicting population connectivity. Colours correspond to genetic clusters in Fig. 2c.

Cluster analyses of 128 brown bear samples (Figs. 1a, S1A, B and Table S1), based on allele sharing distances and depicted using non-hierarchical (i.e., principal coordinate analyses (PCoA)) and hierarchical clustering (i.e., biological neighbour-joining (bioNJ)^41^ and the ordinary least squares (OLS) version of the minimum evolution principle), revealed that brown bears on the Eurasian continent split into a western and an eastern clade (Figs. 1a, b, f, i and 2a). The western Eurasian clade comprises individuals from Europe, Fennoscandia, and western Russia (including Ural Mountains), while the eastern Eurasian clade comprises individuals from Siberia and the Far East.

The boundary between these clades appears to be in western Siberia, west of the river Yenisei. Low similarity was observed between individuals occurring on either side of the contact zone (Figs. 1a, b and S1C, D), with minimum-spanning tree analyses suggesting that eastern clade individuals in eastern Siberia were more similar to western clade individuals in the remote Ural Mountains than to a nearby western clade individual sampled just west of the Ob River (Fig. 2a). These findings suggest an abrupt discontinuity rather than a gradual transition. Individuals sampled east of the discontinuity (‘CentreRus’) were closely related to each other (Fig. S1E).

In the western Eurasian clade, southern Scandinavian individuals clustered away from continental European individuals, including Iberia (Figs. 1b and S2A). The OLS algorithm clustered Middle Eastern individuals together with continental European individuals (Fig. S2B), whereas the bioNJ algorithm clustered Middle Eastern individuals with the Himalayan individual (Fig. 1f). Either topology resulted in discrepancies between tree path lengths and true genetic distances for these samples (Figs. 1e and S2B), suggesting non-tree-like population demographics. A similar discrepancy between path length and genetic distance was observed for various other population pairs, including polar bears and North American bears, as well as Kamchatkan bears and south Alaskan bears (Figs. 1e, f and S2C).

The population structure inferred from a dataset of ~3000 microsatellite loci mirrored the population structure suggested by autosomal SNP data, except that central Russian bears and Kamchatka bears were excluded from the eastern Eurasian clade (Fig. S2D).

### X-chromosomal population structure

The X-chromosomal dendrogram (Fig. 1i), obtained by bioNJ clustering of Euclidean distances calculated from X-chromosomal SNP data, was mostly consistent with the autosomal phylogeny and supported the existence of a western Eurasian and an eastern Eurasian clade, excluding the Middle Eastern and Himalayan bears. However, there were several discrepancies (Fig. 1i). Unlike autosomal data, X-chromosomal data suggested that Amur bears are highly divergent from all other brown bears. X-chromosomal data also suggested that Kamchatka bears are more similar to Aleutian and Kodiak bears, whereas autosomal data suggested these bears are more similar to eastern Eurasian bears. Furthermore, X-chromosomal data suggested that Admiralty Island bears constitute a monophyletic cluster with Baranof Island and Chichagof Island bears, whereas autosomal data suggested that Admiralty Island bears are equally similar to bears of mainland south-eastern Alaska. Lastly, X-chromosomal data suggested that bears northeast of Admiralty Island (‘ABCcoast1’) are more similar to interior Alaskan bears than suggested by autosomal data.

### Y-chromosomal population structure

The Y-chromosomal phylogeny deviated strongly from the autosomal and X-chromosomal phylogeny, both in terms of topology and the ratio between internal and external branch lengths (Fig. 1g). Apart from the highly divergent haplotypes found in the Middle East and the Himalayas, three clades were observed with a split time of approximately 80 kya (Figs. 2d and S3), assuming a mutation rate of 1.3 × 10^−9^ per site per year^42^. Although confounded by considerable geographical overlap, the haplotypes of these three clades were predominantly located in (1) western Eurasia, (2) central Eurasia, and (3) eastern Eurasia and northern America combined (Fig. 2c–e). Further subdivision revealed clusters which separated prior to the LGM, including a cluster of Y-chromosomal haplotypes uniquely observed in the contiguous United States (Fig. 2c–e).

### MtDNA population structure

The mtDNA dataset revealed the same phylogeographical patterns as reported by previous studies (Figs. 1a, 2b and S3), and identified the mitochondrial haplotype clusters 1a (southern Scandinavia and Spain), 1b (Europe), 2a (ABC-Islands), 2b (polar bears), 3a (Eurasia, Alaska and Hokkaido Central), 3b (Canada and Hokkaido East), 3c (Middle East), 4 (West Coast, Hokkaido West and Himalayas East) and 6 (Himalayas West), with TMCRA-estimates pre-dating the Eemian interglacial (130–115 kya) (Fig. S3). The geographically wide spread haplotype cluster 3a could be subdivided in subclusters centred on Fennoscandia, Eastern Europe, Central and Eastern Russia, Hokkaido, Kamchatka and Alaska (Fig. 2b).

### Modelling climate suitability

**Fig. 3:**
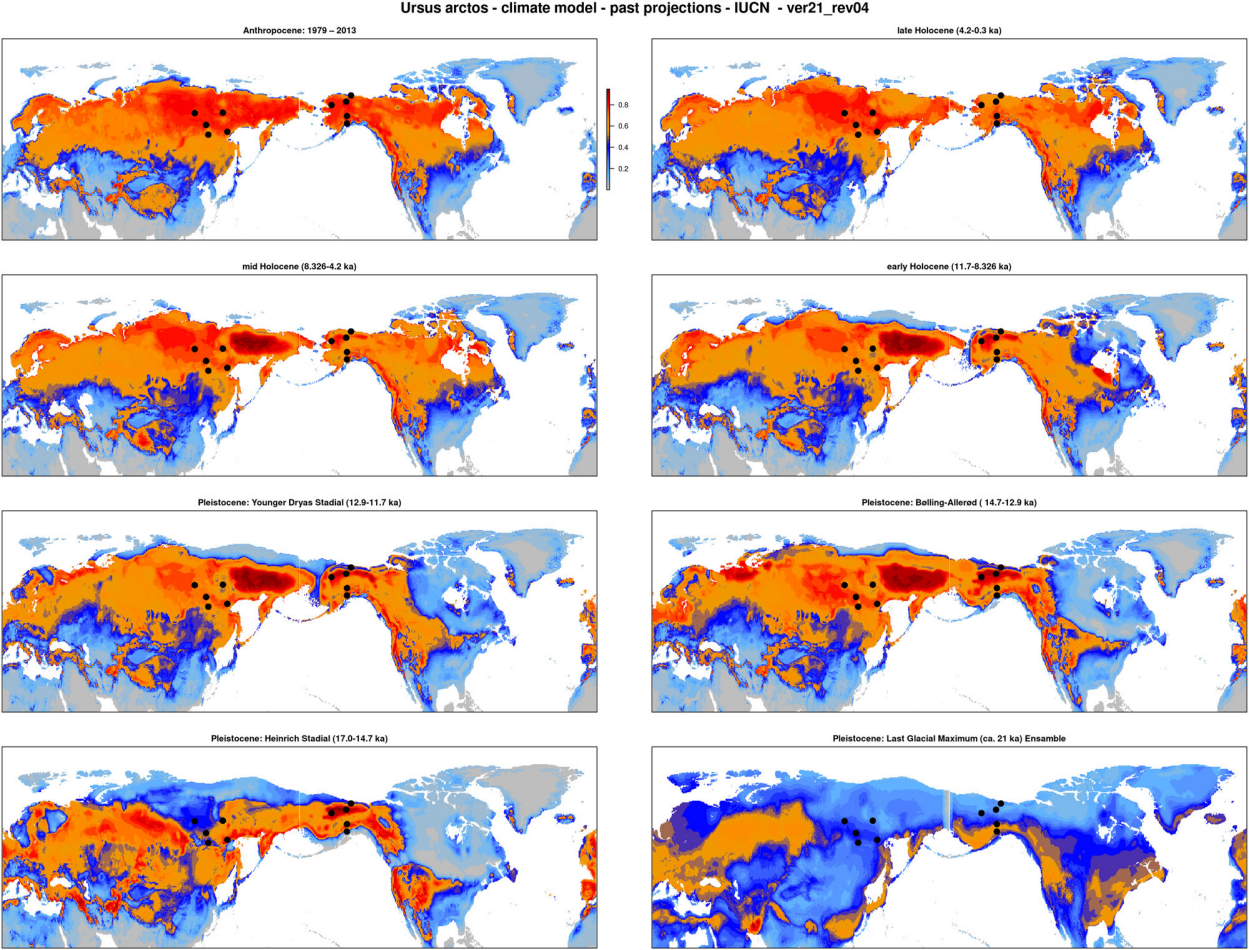
Climate suitability modelling indicates that brown bears were absent from interior Alaska and eastern Siberia during the Last Glacial Maximum. Climate suitability projections on brown bear climatic suitability ranges for various time periods suggest that the species range of brown bear has remained relatively stable (at least in Eurasia) for nearly 20ky, including during stadials. In contrast, the projection for the brown bear range during the Last Glacial Maximum differs markedly from the present-day species range, especially with regard to climatic suitability in eastern Siberia. Note that these maps show climatic suitability only. Not considered are potential additional limitations caused by biotic factors (e.g., vegetation), abiotic factors (e.g., ice cover) and migration barriers. Black dots indicate the geographic location of samples of populations which are marked by f3-analyses as admixed (‘Yakutia’ and ‘Alaska’, see Fig. 4a, b).

Projections of the climate suitability models of brown bears to past climate conditions (Fig. S4A) suggested relatively stable climatic suitability ranges throughout the Late Glacial and the Holocene, including during stadials (Fig. 3). The inferred climatic suitability map resembled the present-day map, with a contiguous zone of high climatic suitability spanning across northern Eurasia and north-western North America (Fig. 3 and S4B). One exception was western Europe (including Britain), for which high suitability was inferred throughout the Late Glacial, but low suitability during the Holocene (Fig. 3).

In contrast to the relative climate suitability stability during the Bolling-Allerod interstadial, Younger Dryas stadial and Holocene, the LGM projection differed markedly from the present-day projection (Fig. 2e). Most notably, low LGM climatic suitability was inferred for eastern Siberia, separating the zone of climatic suitability into two main parts: a broad western range spanning from western Europe to central Russia (including western Siberia), and a narrower eastern range spanning along the coastlines of eastern Eurasia and northwestern North America (Fig. 3).

### Admixture analyses

**Fig. 4:**
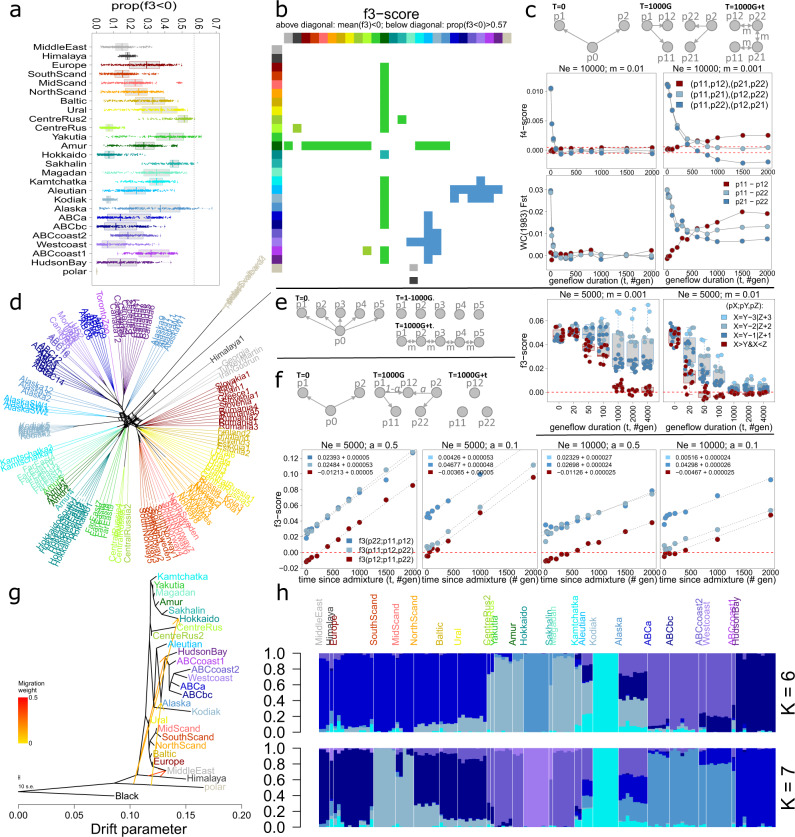
Admixture analyses suggest hybridisation zones in Alaska and eastern Siberia (‘Yakutia’). **a** Boxplots with overlaying stripcharts, depicting for all 7800 population triplets (A;B,C) the proportion of 50 Kb windows with a f3-score below zero, with the putatively admixed population A along the *y*-axis. The dashed line denotes an arbitrary cut-off at 0.57. **b** Matrix highlighting population triplets with negative genome-wide f3-score (above diagonal) or high proportions (>0.57) of 50 Kb windows with a negative f3-score (below diagonal), for all populations triplets (A;B,C). Colours along the *x*- and y-axes represent donor population B and C, while field colours denote admixed population A (i.e., Yakutia, Alaska, Sakhalin and CentreRus2). **c** Consistent with expectations, SLIM2 genetic forward-in-time simulations indicate that signals of former population structure (here estimated using f4- and F_ST_-estimates) are lost in approximately 1/m generations, with *m*, migration rate, denoting the proportion of individuals that are immigrants. Initially, population p12 clusters with sister population p11, but gene flow ultimately causes p12 to cluster with unrelated population p22. **d** Phylogenetic network constructed with the ‘Neighbor-Net’ algorithm, based on genome-wide uncorrected genetic distances. The tiplabels of the outgroup (American black bears) are not shown. Polar bears cluster either with black bears or North American brown bears. Bears from the Middle East cluster either with European bears or Himalayan bears. Southwestern Alaskan (‘Aleutian’) bears cluster either with Alaskan bears or Kodiak bears. **e** SLIM2 simulations indicate that negative f3-scores can arise as a result of isolation-by-distance. **f** SLIM2 simulations indicate that, as a result of genetic drift, f3-scores increase every generation with a fixed value of ¼Ne, causing evidence of past pulse admixture events to eventually disappear. **g** Treemix maximum likelihood phylogeny with the four strongest admixture edges. Consistent with MSC-based analyses (Fig. 5A), the admixture edges suggest gene flow from Himalayan bears into Syrian bears (Middle East), as well as gene flow from polar bears into Hokkaido and North American brown bears. **h** Stacked barplots, generated with the R package LEA, depicting inferred ancestry coefficients assuming 6 or 7 ancestral populations. For each pair of closely related individuals, only one individual was included in the analysis.

Of all possible 7800 population triplets (A;B,C), 24 triplets had a negative f3-score, and these 24 triplets suggested two admixed (A) populations: Alaska and Yakutia (Figs. 4b and S5A). Alaskan bears had as one donor Beringian bears (Aleutian, Kamchatka, Kodiak) and as second donor North American bears (ABCa, ABCbc, ABCcoast, ABCcoast2, HudsonBay and West Coast) (Fig. 4b). Yakutian bears had as one donor Ussuri bears (south of the Amur River), and as second donor either Beringian bears (Aleutian, Kamchatka, Kodiak) or bears from western Eurasia (Fig. 4b). Roughly similar results were observed when using the proportion of negative 50 kb windows as metric instead of genome-wide mean scores (Figs. 4a and S5B).

Consistent with the outcome of f3-analyses, a Neighbor-Net phylogenetic network, based on genome-wide uncorrected genetic distances, suggested 1) that bears from the Middle East cluster either with European bears or Himalayan bears, 2) that southwestern (Aleutian) bears cluster either with Alaskan bears or Kodiak bears, and 3) that polar bears cluster either with black bears or North American brown bears (Fig. 3d).

The topology of the maximum likelihood phylogeny inferred by the software Treemix^43^ (Fig. 4g) was consistent with the topology of the bioNJ hierarchical clustering dendrogram, except for the position of bears from the Middle East (Fig. 1a). The strongest migration edge suggested gene flow from the Himalayas into the Middle East. Migration edges with lower weight all suggested gene flow from polar bears into brown bear populations, more specifically into North American brown bears, Hokkaido bears and Alaskan bears (Figs. 4g and S5C). These results are generally consistent with the hierarchical clustering residuals, i.e., the observed discrepancies between true genetic distances and the path lengths in hierarchical structuring dendrograms (Figs. 1e, f and S2C).

When assuming six or seven ancestral populations – optimum K according to cross-entropy scores (Fig. S6A) – admixture analyses suggested that Alaskan and Aleutian individuals share genetic variation with eastern Eurasian bears as well as North American bears (Fig. 4h and S6B–D), and that individuals from Siberia (‘CentreRus2’, ‘CentreRus’ and ‘Yakutia’) share genetic variation with bears from western Eurasian and from the Far East (Fig. 4h). Westcoast bears shared genetic variation with ABC-Islands bears and Nunavut bears (‘Hudson Bay’). Bears in Fennoscandia (‘Mid-Scand’ and ‘North-Scand’) shared genetic variation with southern Scandinavian bears as well as European bears (Figs. 4h and S6B–D). The observed discrepancy with other admixture analysis methods – e.g., the admixture signal for ‘Westcoast’ is not supported by f3-analyses – could result from the confounding factor of population size differences^44^.

### Genetic simulation outcomes on population structure and admixture signals

We performed forward-in-time genetic modelling simulations, using the software SLIM2^45^, to examine to which extent population-genetic analyses on biallelic SNP datasets can reveal former population structure. We simulated a demographic scenario in which two isolated populations (p1 and p2) split at *t* = 0 each into two subpopulations (p11, p12 and p21, p22), creating a stepping-stone population structure with constant migration rates (*m*) between populations, with peripheral populations p11 and p12 (Fig. 4c and Table S2). The simulations indicated that the f4-score of the population quartet which represents the new population structure ((p11,p21),(p12,p22)), converges to zero within approximately 1/*m* generations (Fig. 4c). Similarly, Weir & Cockerham Fst-values between population pairs reach their new equilibrium values after approximately 1/m generations (Fig. 4c), consistent with theoretical expectations^46^.

Next, we investigated the duration required for admixture signals to appear in the face of continuing gene flow. We simulated a star population structure of five populations (p1, p2, p3, p4, p5) which at *t* = 0 transforms into a stepping stone population structure (isolation-by-distance in order of their population number), with a *Ne* of 5000 individuals per population (Fig. 4e and Table S3). Negative f3-scores, indicative of admixture, were observed after approximately 1/*m* generations (Fig. 4e). These negative scores were observed for all population triplets (pX;pY,pZ) for which X > Y and X < Z (Fig. 4e), implying that negative f3-scores may indicate indirect gene flow. This might explain our empirical finding that Yakutia and Alaska have negative f3-scores for a wide range of population triplets (Fig. 4c).

Lastly, we investigated the duration required for genetic drift to erase admixture signals (i.e., f3 < 0) originating from a pulse admixture event occurring at *t* = 0 (Fig. 4f and Table S4). We simulated a demographic scenario in which migrants from two isolated populations (p1 and p2) founded at *t* = 0 an admixed population (p12), with admixture ratios 1:1 (a = 0.5) and 1:9 (a = 0.1; Fig. 4f). Our findings indicated that the f3-score increases linearly with time, rising each generation with 1/(4·*Ne*). Thus, given a fixed population size, the time for the f3-score to become positive is -b·4·*Ne* generations, with b (the intercept) representing the initial f3-score directly following the admixture event. In this respect, the f3-score differs from the f4-score (i.e., D-statistics), of which signals of past shared ancestry can be erased by subsequent gene flow (Fig. 4c), but not by random fixation of alleles^47^.

#### Multispecies coalescent (MSC) analyses

**Fig. 5:**
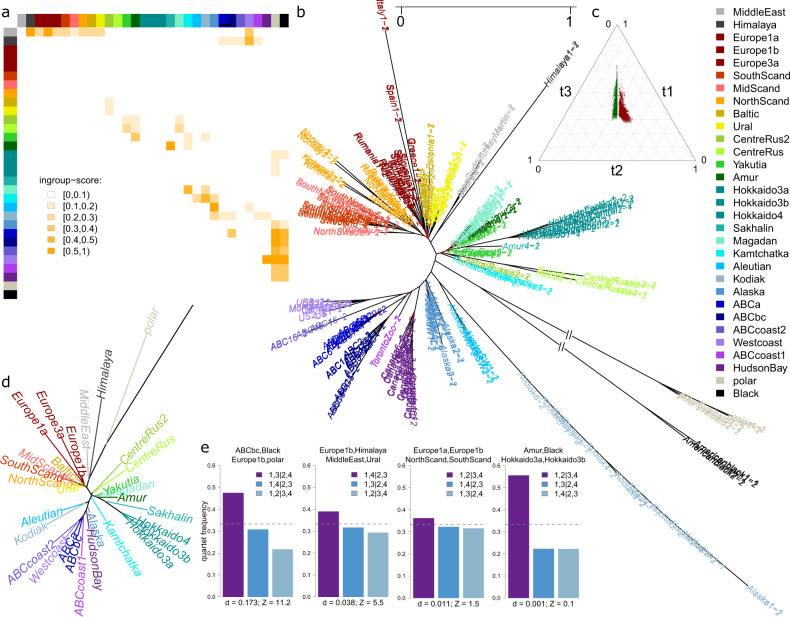
Multispecies coalescent (MSC) based analyses: testing for inequality of alternative quartet topology frequencies. Graphical summary of MSC-based analyses on 3075 haploblocks of at minimum 25 kb length. Haploblocks were detected using Plink, and each haploblock dataset was phased with Beagle, generating two haplotypes per individual (which were randomly marked −1 and −2). Quartet frequencies were calculated and aggregated per population quartet using the software Twisst. The populations of Europe and Hokkaido were subdivided according to mtDNA clusters. Quartet frequencies were defined as t1, t2 and t3, referring respectively to the species tree topology q1, the most frequent alternative topology q2, and the less frequent alternative topology q3. **a** Heatmap depicting the ‘ingroup-score’, which we define as the proportion of quartets for which populations *x* and *y* group together in q2 and for which the hypothesis of equal alternative quartet topology frequencies (i.e., t2 ≈ t3) is rejected (based on a *z*-score threshold of 5), relative to the total number of quartets in which populations *x* and *y* are both present but do not group together in q1. The population pairs which occur most repeatedly as an ingroup of the more frequent alternative topology are: Alaska-Aleutian, Amur-Sakhalin, ABCbc-polar, Europe1b-MiddleEast, MiddleEast-Himalaya, ABCa-polar, polar-Westcoast, and Black-Westcoast. Outside of North America, an excess of polar bear genetic material is also found on Hokkaido Island. **b** ASTRAL supertree based on an input dataset of 3075 haploblock bioNJ trees. Nodes with local posterior probabilities below 0.8 are marked in red. Branch lengths are in coalescent units (2N generations). **c** Simplex depicting quartet topology frequencies (t1, t2 and t3) of all 31465 quartets. Red: quartets for which the null hypothesis of equal alternative quartet topology frequencies (i.e., t2 ≈ t3) was rejected, using a conservative z-score threshold of 5. Green (mirrored relative to y-axis): null hypothesis not rejected. **d** Reference phylogeny, used to distinguish between the species tree quartet topology (q1) and the two alternative quartet topologies (q1 and q2). This reference tree was constructed using Neighbor-joining clustering based on Nei’s genetic distances between populations, calculated from the autosomal SNP dataset. The tiplabel of the outgroup (American black bears) is not shown. **e** Barplots depicting quartet topology frequencies for an arbitrary subset of four quartets (out of all 31465 quartets). Dashed lines indicate the one-third quartet topology frequency threshold. Unequal frequencies of t2 and t3 suggest introgression between ABC brown bears and polar bears as well as between Syrian bears (‘Middle East’) and European bears. In contrast, no statistical support was found for an excess of discordant gene trees for South Scandinavian bears and Spanish bears (which both carry mtDNA haplotype 1b), and also not for bears from the Amur region and from Hokkaido which share mtDNA haplotype 3a. The latter findings question existing hypotheses on brown bear (postglacial) migration routes.

We generated bioNJ gene trees for a dataset of 3075 phased, highly polymorphic haploblocks. The topology of the Astral^48^ supertree (Fig. 5b) largely reflected the topology of the bioNJ phylogeny generated from autosomal SNP data (Fig. 1f), albeit with more distinct population clustering (e.g., Scandinavia versus Europe, and western Eurasia versus eastern Eurasia). Long branch lengths (in units of 2Ne generations) indicated low effective population sizes for brown bear populations in Kodiak, Himalaya, Spain and Italy (Fig. 5b).

Quartet topology frequencies were calculated and aggregated per population using the software Twisst^49^. For these analyses, the populations Europe and Hokkaido were subdivided according to mtDNA haplotype (i.e., Europe1a, Europe1b, Europe3a, Hokkaido3a, Hokkaido3b, Hokkaido4), resulting in 31 population clusters. For each quartet, the species tree topology was determined through comparison to the topology of a neighbour-joining phylogeny constructed from Nei genetic distances between populations (Fig. 5d). When applying a z-score threshold of 5, for 4176 (13.3%) out of all (31 choose 4) 31.465 quartets, the null hypothesis of equal alternative frequencies was rejected (Fig. 5c, e).

The population pairs which occurred most repeatedly as an ingroup of the more frequent alternative topology (here defined as the ingroup-score) were, in decreasing order: Alaska-Aleutian, Amur-Sakhalin, ABCbc-polar, Europe1b-MiddleEast, MiddleEast-Himalaya, ABCa-polar, polar-Westcoast, and Black-Westcoast (Fig. 5a). Using an arbitrary ingroup-score threshold of 0.1 (Fig. 5a), discordant signals were observed for Beringian population pairs, for central and eastern Eurasian population pairs, and for population pairs involving comparisons between North America and Himalaya, and population pairs involving comparisons between western Eurasia and the Middle East (Fig. 5a). The results furthermore indicated that Hokkaido bears also carry relatively high proportions of polar bear genetic material, albeit to less extent than North American bears (Fig. 5a). The suggestion that North American brown bears do not only share a relative excess of genetic material with polar bears, but also with American black bears (Fig. 5a), could be an indirect consequence of sharing ancestral material with polar bears.

Individuals from southern Scandinavia and Spain, which share mtDNA haplotype 1a, did not occur as ingroup in any quartet with significantly different alternative frequencies (Fig. 5a, e). The same was true for individuals from Primorsky Krai (‘Amur’) and central Hokkaido which share mtDNA haplotype 3a (Fig. 5a, e).

### Genetic diversity and population differentiation

**Fig. 6:**
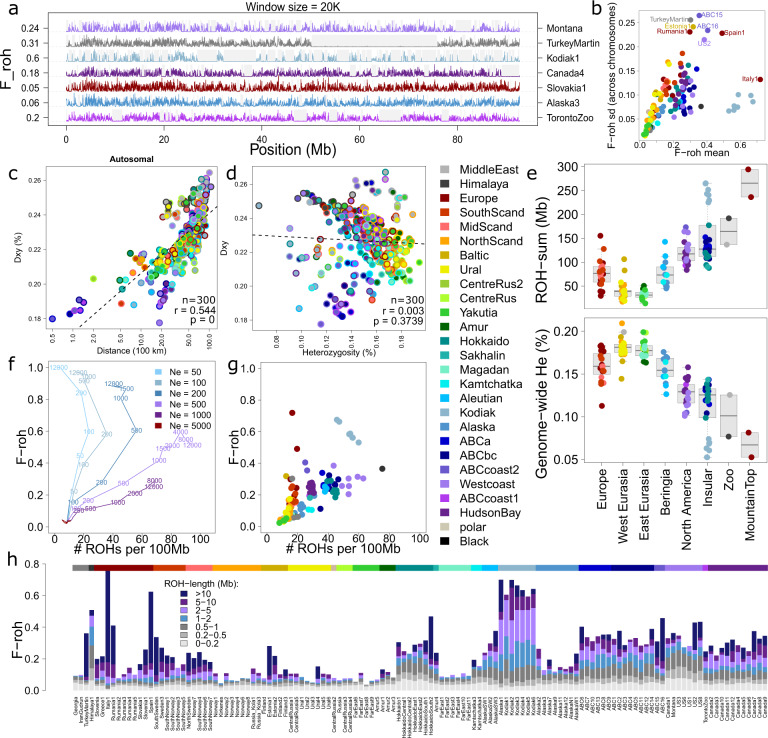
Heterozygosity analyses reveal recent inbreeding on isolated mountain-tops, and ancient population bottlenecks in Kodiak and grizzly bears. **a** Variation of heterozygosity levels (*He*) across a randomly chosen chromosome, for a random subset of samples, depicting He-levels of non-overlapping 20 kb windows, with grey segments highlighting the genomic regions which are marked as run of homozygosity (ROH) by the newly developed ROH-detection software Darwindow. Values along the y-axis represent sample and chromosome-specific F_ROH_-values (proportion of chromosome marked as ROH). **b** Standard deviation of sample-specific F_ROH_-values across chromosomes versus genome-wide mean F_ROH_-values. High standard deviations are indicative of recent inbreeding events. **c** Mantel plot showing, for all (25 ∙ 24/2=) 300 brown bear population pairs, mean genome-wide genetic distance (D_XY_) versus geographic distance. **d** D_XY_-values against mean genome wide He-estimates, for all possible 300 population pairs. Population pairs involving bears from Middle East and North America affect the significance of the linear regression model. **e** Boxplots depicting sample-specific ROH-length and genome-wide *He* estimates, with samples grouped according to sample origin. Centre lines indicate median, and box limits indicate upper and lower quartiles. **f** SLIM2 genetic forward-in-time simulations indicate that following a population size reduction (from an initial *Ne* of 5000 individuals), and given sufficient time, ROH-distributions converge to a new equilibrium, with the relationship between F_ROH_ and number of ROHs depending on the new *Ne*. Shown are the number of generations since the population size reduction. **g**, **h** Sample specific ROH-distributions. The colour bar above the stacked barplot indicates population assignment.

We calculated genome-wide heterozygosity and screened the genomes for runs of homozygosity (ROH) using a purpose-built tool named Darwindow, which allows visual examination of the levels of heterozygosity in genomic regions marked as runs of homozygosity (Figs. 6a and S7A). An inverse linear relationship was observed between genome-wide heterozygosity (*He*) and the proportion of run of homozygosity (F_ROH_, Fig. 6e). The mean sample *He* estimate (excluding the Himalayan individual with low mean sequencing depth) was 0.157% (sd.: 0.028%). Exclusion of ROHs raised the mean sample *He* estimate (excluding the Himalayan individual) to 0.195% (sd.: 0.014%) (Fig. S7B). Heterozygosity levels within ROHs were inversely related to ROH-length, with the longest ROHs (>40 Mb) having a heterozygosity level of approximately 0.003% (Fig. S8). This value is considerably higher than predicted by coalescent theory for ROHs stemming from identity by descent (i.e., *He* = 2·ut), suggesting these values are biased upwards due to genotype errors.

Present-day genetic diversity was found to vary between and within geographical regions (Fig. 6e). Bears in Eurasia had generally higher *He-*levels and lower F_ROH_-scores than bears in North America (Fig. 6e). Fennoscandian bears exhibited generally lower *He-*levels than other Eurasian bears. The lowest He-levels and the highest number of and/or longest runs of homozygosity were observed in insular populations (especially Kodiak Island), mountain relict populations (Cantabrian bears in Spain and Apennine bears in Italy) and individuals from glacier-bounded inlets along the rugged coastline of south-east Alaska (‘ABCcoast1’ and ‘ABCcoast2’, Fig. 6e, g, h). The F_ROH_ estimates of these wild bears approached or exceeded the F_ROH_ value of a zoo animal (‘TurkeyMartin’) with a pedigree based inbreeding coefficient of 0.3. The ROH-content of Kodiak Island bears was relatively constant across chromosomes (Fig. 6b) as well as across individuals (Fig. 6h), suggesting their high F_ROH_ values were not caused by recent chance inbreeding events, but instead by an ancient population bottleneck or long-term small effective population sizes.

*He*-estimates ranged between 0.05% and 0.21% (Fig. 6e), and D_XY_-estimates (mean uncorrected genetic distance between populations) ranged between 0.17% and 0.27% (Fig. 6c, d). The autosomal dataset revealed a significant correlation between D_XY_ and the geographical distance of population pairs (linear regression analysis for autosomal data: n = 300, r = 0.55, and p < 0.001), regardless of the autosomal clade (western Eurasian, eastern Eurasian or North American) to which a population belonged (Fig. 6c). The correlation between D_XY_ and the mean heterozygosity of population pairs was significant only after exclusion of samples from Kodiak, the ABC-Islands and the Middle East (Fig. 6d).

### Genetic simulation outcomes on ROH-patterns

We performed forward-in-time genetic modelling simulations, using the software SLIM2^45^, to investigate the dependency of ROH-distributions (number of ROHs per length bin) on time (number of generations) and effective population size (*Ne*). The simulation outcomes showed, as expected, that small populations are characterised by few, long ROHs, medium-sized populations by many short ROHs, and larger populations by few short ROHs (Fig. 6f and Table S5). The time required for ROH-distributions to reach an equilibrium (assuming a fixed population size following a population-size change) is positively related to Ne, and ranges from 10^2^ to 10^4^ generations. These simulation outcomes suggest that the ROH-distribution of the genetically impoverished Kodiak bears can arise in populations with a long-term, fixed *Ne* of approximately 200 individuals (Fig. 6f, g).

## Discussion

The analysis of 128 brown bear genomes allows us to reconstruct a comprehensive picture of the contemporary, range-wide brown bear population structure. The observed genetic structure is generally consistent with the geographical locations of extant populations (as evidenced by a significant isolation-by-distance trend (Fig. 6c)), and thus does not mirror the prominent discontinuities in the geographical distribution of mtDNA haplotypes^23^. Notably, no genomic evidence exists for a dual ancestry of Fennoscandian bears, as proposed earlier based on mtDNA^39^. Bears from south of the River Dal do not cluster with Iberian bears, with whom they share mtDNA haplotype 1a (Figs. 1a and 5a, e)^26,35,39^. Similarly, whereas mtDNA suggests high genetic dissimilarity between Alaskan (mtDNA haplotype 3a) and other North American brown bears (which carry mtDNA haplotypes 3b and 4)^50^, genomic analyses instead indicate that Alaskan bears, with the exception of those from southwestern Alaska (‘Aleutian’), are genetically more similar to North American bears than to bears in Eurasia (Fig. 1c, f, i). As reported previously, ABC-Islands brown bears cluster with other Northern American brown bears, not with polar bears, despite sharing mtDNA haplotypes from clade 2^11,17^. Likewise, Hokkaido bears cluster as a monophyletic unit, and not paraphyletically with other populations as they do in terms of mtDNA (haplotypes 3a, 3b and 4)^51^.

These incongruences between nDNA and mtDNA clustering emphasise the uncertainty associated with drawing conclusions solely from a single, non-recombining locus such as mtDNA. The co-occurrence of mtDNA haplotype 2 in polar bears and ABC-Islands brown bears, which initially was proposed to support the hypothesis that polar bears evolved from within the lineage of brown bears, famously illustrates how multi-locus genomic analyses are crucial for an accurate understanding of evolutionary history^11^.

Unlike what is observed for polar bears and ABC-Islands brown bears (Fig. 5a, e), the null hypothesis of equal alternative quartet topology frequencies cannot be rejected for Iberian and southern Scandinavian bears, nor for Hokkaido bears and nearby mainland bears carrying mtDNA haplotype 3a (Fig. 5a, e). These findings, which suggest that these disjunct geographical distributions of mtDNA haplotypes stem from incomplete lineage sorting (ILS, i.e., random genetic drift of ancestral variation) and not from gene flow or ancestral population structure, challenge several hypotheses previously inferred from mtDNA analyses. More specifically, they question the notion that Hokkaido was colonised during multiple migration waves^25,51^, as does the monophyletic clustering of Hokkaido Y-chromosomal haplotypes (Fig. 2d). The findings also appear inconsistent with the classic brown bear recolonisation model, which envisions postglacial recolonisation of Fennoscandia from both an Iberian (haplotype 1a) and a central European (haplotype 3a) refuge^4,21,34,35^. Ancient-DNA-studies have previously thrown doubt on this classic postglacial model, and indicated that after the LGM, and contrary to the present-day situation, southern Scandinavia was temporarily home to both haplotypes (1a and 3a)^32^.

The mtDNA break in southern Scandinavia is located in an area of low bear-population density, which separates two adjacent female concentration areas^39^. This lowland extends for over 100 kilometres and is rarely crossed by females^39^. Likewise, the occurrence of the three distinct mtDNA haplotypes (3a, 3b, 4) on Hokkaido Island coincides with the geographical ranges of three mountain areas, which are separated by unforested lowlands. Polar-bear like haplotype 2a is exclusively found on islands which are surrounded by water barriers which are of sufficient width to prevent female crossings^52^, and which belong to an archipelago harbouring numerous other endemic mtDNA lineages^53^. In all three cases, the divergence times of the mtDNA haplotype clades vastly predate the population split times suggested by the timing of geological events, such as the emergence of land bridge islands or the establishment of LGM glacial refugia. It thus appears that many present-day brown bear mtDNA disruptions arose as a result of random differential fixation of ancestral alleles, facilitated by female philopatry and enforced by geographical barriers.

In several locations, including the ABC-Islands, Ireland and western Europe, the emerging dominant mtDNA haplotype happened to be an introgressed polar bear allele. The deep split times of these haplotypes relative to those of present-day polar bears (Fig. S3) suggests that these alleles were first introduced in the brown bear gene pool during ancient, pre-Eemian hybridisation events^54^. Given the stochastic nature of genetic drift and the vast potential for population turnovers ever since, the current geographical ranges of introgressed mtDNA haplotypes may be uninformative about the whereabouts of these introgression events. The present-day confinement of haplotype 2a to the ABC-Islands may simply be a case of paleoendemism^53^, an equilibrium state which establishes itself more slowly and therefore is still not reached in species with higher population sizes and smaller dispersal distances^55^.

The proportion of polar bear ancestry in present-day brown bear populations appears to depend on the extent to which a population has been isolated from the Eurasian continent, either by a geographical barrier or through isolation-by-distance (Fig. S2c). Possibly, hybrid populations which best preserved polar bear genetic material are those which interacted the least with descendants of a lineage of pure brown bears from mainland Eurasia (Fig. 4c). This could explain the relatively high proportions of polar bear DNA on Hokkaido Island (Figs. 4g, 5a and S2C), as well as the variation in polar bear ancestry proportions among the ABC-Islands^15^ (see below for further discussion).

An alternative potential explanation for mitonuclear discordances, other than ILS, is that nuclear DNA and mtDNA may convey signals of population structure from different moments in time (e.g., contemporary versus ancestral population structure). As reemphasised by our genetic simulations, the establishment or removal of a dispersal barrier can quickly erase genomic evidence of the former population structure^46,56,57^, including even signals obtained from Four Taxon Tests (Fig. 4c). In contrast, mtDNA, being non-recombining and inherited strictly maternally, is unaffected by male-mediated gene flow and therefore can preserve the signatures of former population structure, especially in species with female philopatry. Hence, the discordant distributions of mtDNA haplotypes may provide a window into the past (albeit likely distorted by single-locus stochastic processes), while nDNA mainly represents contemporary population structure. This could, for instance, explain why Alaskan brown bears cluster with Russian bears in terms of mtDNA, but with North American bears in terms of autosomal DNA (Fig. 1a–f).

Male-mediated gene flow affects X-chromosomal loci less than autosomal loci: immigrant males pass on their X chromosomes to female offspring only. Therefore, X-chromosomal population structure may lag behind autosomal population structure, and thus retain longer the vestiges of a former population structure. X-chromosomal data suggest that Kamchatkan bears cluster with southwestern Alaskan bears (which is consistent with morphological-based inferences^7^), while autosomal data suggest that Kamchatkan bears are genetically more similar to bears of eastern Eurasia (Fig. 1e, f, h, i). If Kamchatkan bears and southwestern Alaskan bears derive from a common ancestral Beringian population, male immigrants from other populations could have driven a wedge between the sister populations following the flooding of the Bering Strait, but less so in terms of X-chromosomal loci.

A mismatch in temporal resolution of mtDNA and nuclear DNA markers may not only cause discordant mtDNA phylogeographic breaks, but conversely also nuclear-DNA phylogeographic breaks too recent to be detected by mtDNA studies. Our hierarchical and non-hierarchical structure analyses on multi-locus (autosomal and X-chromosomal) datasets revealed a pronounced genomic discontinuity in western Siberia, apparently situated between the Ob and the Yenisei (Figs. 1b, f, i and 2a), dividing two distinct Eurasian clades (i.e., a western and eastern). This division roughly coincides with the currently accepted boundary between the European (*U. a. arctos*) and East Siberian (*U. a. collaris*) subspecies, originally deduced from morphological and behavioural data^9^. Because individuals on either side of the phylogeographic break carry haplotype 3a (Fig. 1a), the discontinuity is not evident from mtDNA markers. Only on closer inspection an indistinct geographical division appears to exist between variants of this haplotype (Fig. 2b)^20,31^.

The relatively high genetic differentiation between the western and eastern clade, in comparison to the lower divergence levels within the two clades itself, might suggest that the genomic discontinuity represents a hybridisation zone between two ancient lineages which existed long before anthropogenic range fragmentation. On the other hand, the narrow width of the hybridisation zone argues against a secondary contact zone following postglacial recolonisation. Theory predicts that, in the absence of a migration barrier, genetic exchange would have widened the hybridisation cline over time, resulting in a more gradual west-east transition^56,58^. Therefore, the abruptness of the discontinuity could indicate a more recent, Holocene barrier to gene flow^57^.

The western Siberian lowlands have been reported to sustain a relatively low density of brown bears, possibly due to low primary productivity and an abundance of wetlands^59^. To the east, roughly demarked by the Yenisei, lays the transition between the evergreen forests of spruce, pine and fir of western Eurasia and the deciduous forests of permafrost-tolerant larch in eastern Eurasia. Future studies focusing on this area may increase the local sample density to verify the width and age of the hybridisation zone, and elucidate if any habitat condition^60,61^ act or have acted as an ecological barrier to gene flow.

If the genomic discontinuity is indeed of relatively recent origin, it may have contributed to obscuring the population structure initially created by postglacial recolonisation. The f3-analyses highlighted two admixed populations, ‘Alaska’ and ‘Yakutia’ (Fig. 4a, b), corroborating morphological-based inferences that these populations may be of hybrid origin^9,10^. Climate suitability projections (Fig. 3) and previously published LGM vegetation maps^62,63^ suggest that both populations occur in regions with low suitability for brown bears during the LGM. Although other scenarios – such as recent isolation by distance rather than an ancient admixture event (Fig. 4e) – cannot be excluded, the consistency between the outcomes of admixture analyses and climatic suitability analyses lends credence to the hypothesis that the negative f3-scores for Yakutia and Alaska are a relic of secondary contact following postglacial recolonisation.

Our genetic simulations revealed that, after cessation of gene flow, genetic drift causes the f3-statistic to increase linearly in time, with 1/(4·*Ne*) each generation (Fig. 4e). Assuming an initial magnitude of −0.02 (Fig. 4f) and a *Ne* of 20.000 individuals, genetic drift would require more than 1500 generations to drive the f3-score above zero. Given an average brown bear generation time of 10 years, it is conceivable that present-day weak negative f3-scores are a vestige of secondary contact following postglacial recolonisation.

Further support for the hybrid nature of brown bears in Alaska and eastern Siberia (‘Yakutia’) is obtained from Y-chromosomal data. This dataset reveals that both populations sit on the borders of neighbouring ranges of Y-chromosomal clades, with TMCRA estimates predating the LGM (Fig. 2c, d). On the other hand, the extensive geographical overlap of Y-chromosomal clades in Eurasia (Fig. 2c, d), appears at odds with the notion of a genomic discontinuity in western Siberia, and instead suggests genetic exchange between the western and eastern Eurasian clade (Fig. 2e). At present, our limited sampling in central Asia provides no evidence for population connectivity between the Middle East and central Russia via the Pamir, Tien-Shan and Altai Mountain ranges.

Lower Y-chromosomal diversity is observed in North America (Fig. 2c–e), in line with the overall lower genetic diversity of grizzly bears (Fig. 6e). The contiguous United States are home to endemic Y chromosome haplotypes (‘US6’ and ‘Montana’). The pre-LGM split time estimate of this clade appears to support the scenario of colonisation of North America prior to the LGM. This scenario is also consistent with signals of shared ancestry between North American bears and the basal lineage of brown bears from the Himalaya and the Middle East (Figs. 1f and 5a).

Palynological studies indicate that during the LGM two major isolated forest regions persisted west of the Great Plains (in north-western US and the southern Rocky Mountains)^64^, potentially implying two disconnected continental refuges in present-day contiguous United States. Evidence furthermore exists for a coastal refuge along the southeast coast of Alaska^55^. Possibly, offspring from any of these refuges spread their mtDNA and Y-chromosomal haplotypes across Canada and into Alaska (Fig. 2d). The Y-chromosomal clade which at present occurs at both sides of the Bering Strait (Fig. 2c, d) may derive from an ancestral population which during the LGM inhabited Beringia (Fig. 2e).

Y-chromosomal haplotype sharing between the mainland and Admiralty Island (i.e., between samples ABC7, Canada10 and Alaska12, Fig. 2d) confirms earlier reports of male brown bear crossings^14,52^. Furthermore, the monophyletic clustering of Y-chromosomal haplotypes from the Baranof and Chichagof Islands (BC-Islands) confirms earlier claims that the 7-km wide Chatham Strait, which separates the Admiralty Island from the BC-Islands, effectively prevents male crossings (Fig. 2d). As a result, gene flow may drive a wedge between the ABC-Island bears, causing them to appear as a paraphyletic unit based on autosomal DNA (Fig. 1f)^52^. In contrast, because gene flow is male-mediated, X-chromosomal DNA still identifies ABC-Island bears as a monophyletic unit (Fig. 1i), consistent with a ‘X-chromosomal lagging effect’.

In conclusion, our brown bear population-genomic analyses have revealed numerous discrepancies between single-locus and multi-locus inferences. These incongruences between mtDNA, Y-chromosomal, X-chromosomal and autosomal phylogeographical patterns may be partly caused by stochastic processes which particularly affect single loci, leading to puzzling instances of mtDNA and Y-chromosomal haplotype sharing between unrelated brown bear populations. However, an alternative explanation is that different parts of the genome are, depending on their inheritance properties, differentially affected by male-mediated gene flow. Discordant mtDNA and Y-chromosomal phylogeographical patterns can reveal insights into past demographic processes which have been erased by gene flow and recombination, and therefore remain obscure from multi-locus inferences^52^.

## Materials and methods

### Sample collection

We obtained the short read sequences for 33 brown bear genomes, four polar bears (*Ursus maritimus*) and two American black bears (*Ursus americanus*), publicly available from NCBI’s SRA repository (Table S1 and Fig. 1a)^12,13,15,16,40,51,65^. Next, we selected from our private collections a total of 95 additional samples for sequencing, among them three zoo individuals and 92 wild-caught individuals (Table S1). No animals were killed for this study. Blood samples from zoo individuals were extracted during routine procedures.

The combined brown bear dataset covered the following geographical areas (Fig. 1a): Himalayas (Tajikistan), Middle East (Turkey, Georgia and Iran), Europe (Carpathian, Balkan, Alpine, Apennine and Cantabrian Mountains), southern Scandinavia (mtDNA haplotype 1a, Dalarna and Hedmark), mid-Scandinavia (mtDNA haplotype 3a, Trondelag), northern Scandinavia (Lapland and Russian Kola Peninsula), the Baltic States (including the border region with Finland and Russia), Ural Mountains, central Russia (Krasnoyarsk Territory), Yakutia, Magadan, Kamchatka, Primorye (‘Amur’), Hokkaido, Sakhalin, interior Alaska, southwestern Alaska (here named after the Aleutian Mountain Range), Nunavut territory in northern Canada (here denoted as ‘Hudson Bay’), the ABC-Islands (Alexander Archipelago, in southeastern Alaska), and the North-American Westcoast (here defined as the western contiguous United States and southern British Columbia).

### Sequencing

A total of 95 brown bear individuals (one duplex for verifying data integrity) was sequenced at minimum 10x coverage by BGI Genomics and Novogene Europe, using a DNBSEQ-T7 (BGI Genomics) and an Illumina Novaseq 6000 machine (Table S1 and Fig. 1a). The mean nuclear genome sequencing depth was 9.4 reads per site per sample, with values ranging between 4.6 and 16.4 (Fig. S1). The maximum read depth used for mtDNA genotype calling was limited to the default of 250 reads (defined by the –max-depth flag of bcftools mpileup), resulting in a mean depth of 236.7 reads (range: 203–245) per sample (Fig. S1). The rate of alternative homozygous calls for the reference genome sample was 86 kb out of 1250 Mb of retained autosomal sites, suggesting a genotyping error rate of 0.007% for diploid data. The number of different calls at the Y chromosome between the two copies of the duplex sample was 136 out of 1.35 Mb retained sites, suggesting a genotyping error rate of 0.01% for haploid data.

### Read mapping and genotype calling

Read quality check was performed using the software FastQC and MultiQC^66^. Reads with high adapter content were removed using the software AdapterRemoval v2^67^. Reads were mapped, using the software BWA^68^, against a south-eastern Alaskan grizzly bear reference genome^65^ with chromosome-level resolution^69^, as well as to a full brown bear mitochondrial sequence (NC_003427.1)^70^. Samtools^71^ was used to remove reads with a mapping quality below 20 and/or alignment scores below 100, as well as reads that mapped discordantly or to multiple locations in the genome. Read duplicates were removed using the software picard^72^.

Genotype likelihoods and calls were generated using the bcftools mpileup and call pipeline^73^. When calling genotypes from genotype likelihoods (bcftools calls), the ploidy-level was set according to genome type (i.e., MT, autosomal and sex chromosome) and sample sex. For samples with missing sex information, the sex was determined from levels of missing data at the Y chromosome (Fig. S9).

When calling genotypes from genotype likelihoods (bcftools call), we used the ‘group-samples’ option to assign each individual to its unique group (i.e., we disabled the option of influencing genotype calls based on information from other samples). We established experimentally, through the comparison of heterozygosity scores and examination of the allelic depths of the three genotypes (homozygous reference, heterozygous, homozygous alternative), that this approach yields the most unbiased results for our dataset (which contains an uneven number of samples from highly divergent populations), especially at low read depths (Fig. S10). We did not find evidence for above-average heterozygosity levels near indels (Fig. S11), and therefore did not use the option –SnpGap. Indels were normalised and realigned using ‘bcftools norm’.

The bcftools filter pipeline was used to mask sites with a read depth below three, after establishing experimentally this provided a balance between disposal of useful data and incorrect heterozygosity estimation (Fig. S10A). We retained sites with a total read depth of 675 to 2150 for all 135 individuals combined. We found that higher upper thresholds would include a satellite peak of above average read depths (Fig. S12A). Pseudo-autosomal regions were identified based on deviations of sequencing depth relative to chromosome-wide means, and subsequently excluded from the X-chromosomal and Y-chromosomal datasets (Fig. S12B).

### Genome-wide statistics (He, F_ROH_, D_XY_)

The total number of retained homozygous and heterozygous sites per sample were counted on a sliding-window basis, using non-overlapping windows with a fixed size of 20 kb. The counting was performed using the custom-built tool Darwindow, which depends on the software Tabix^74^ for the extraction of genomic regions, and which subsequently converts the count data into estimates of heterozygosity (*He*) and run-of-homozygosity content (F_ROH_). Based on examination of the sensitivity of ROH-analyses to different settings, ROHs were defined as continuous regions of at least 200 kb (i.e., ≥10 adjacent windows of 20 kb) with an average *He* value below 0.05%.

Uncorrected genetic distances (*d*) for each sample pair were also generated using custom-built Unix and R scripts. For haploid datasets (mtDNA and Y chromosome) we used the formula: *d* = n1/(n0 + n1), in which n0 denotes the number of similar sites and n1 denotes the number of dissimilar sites. For diploid datasets we used the formula: *d* = (n1*0.5 + n2he*0.5 + n2ho)/(n0 + n1 + n2he + n2ho), in which n0, n1, n2he, and n2ho denote the number of sites with the genotype combinations AA/AA, AA/AT, AT/AT, and AA/TT respectively. Thus, the *d* value of a comparison of the genotypes of one and the same individual equals half its genome-wide heterozygosity, which is the expected mean difference when randomly sampling, with replacement, two haplotypes.

For unphased diploid data, the genetic distances between and within individuals (*d* and *He* respectively) reflect mean coalescence times of haplotypes rather than population split times. Dendrograms depicting these genetic distances are therefore expected to exhibit long external branches. Assuming recent population splits, internal branches mainly denote shared genetic drift, not number of substitutions.

For efficiency reasons, autosomal and X-chromosomal *d* estimates were calculated over randomly thinned datasets rather than the full dataset. Prior to the calculations, the haplodiploid X-chromosomal DNA-dataset was converted to diploid format. Sites with missing data for one or both individuals involved in the pairwise comparison were excluded. Population pairwise D_XY_ values were derived from the sample pairwise *d* values, namely as the mean of all possible sample pairwise comparisons between the two populations.

### Y chromosome and mitogenome phylogeny

Dendrograms of Y chromosome and mitogenome haplotypes were generated with the softwares IQtree and RAXML, using default settings, and linearised using the function ‘chronoMPL’ of the R package ape, which implements the mean path length method (Britton et al. 2002). Genetic distance measures were converted into split time estimates (which may serve as lower limits of population split times) assuming a mutation rate of 1.3·10^−9^ per site per year for the Y chromosome^42^ and 1.9·10^−8^ per site per year for the mitogenome^20^.

### SNP data analyses

A subset of biallelic sites was extracted from autosomal chromosomes for SNP data analyses by filtering number of alleles (i.e., 2), on levels of missing data (max 5 percent allowed), and by subsequently thinning the dataset using vcftools^75^, retaining a maximum of one biallelic SNP for every 20 kb. Similar procedures were followed for SNPs present at both sex chromosomes, but the thinning step was omitted in case of the Y-chromosomal data. To facilitate the data analysis, haploid and haplodiploid datasets were converted to diploid.

Plink version 1.90b.20 (Purcell et al., 2007) was used to convert the SNP data from VCF format into PED/RAW and MAP/BIM (using the flags make-bed, recode A, chr-set 95, and allow-extra-chr). SNP data management and analyses were performed in R-4.2.0^76^, using wrapper functions of the R package SambaR version 1.08^77^. The data were imported into R and stored in a genlight object using the function ‘read.PLINK’ of the R package adegenet-2.1.1^78^. The autosomal data set was filtered using the function ‘filterdata’ of the R package SambaR, with indmiss = 0.1, snpmiss = 0.01, min_mac = 2, and dohefilter = TRUE. The number of retained SNPs was 88417, 9607 and 3687 for the autosomal, X-chromosomal and Y-chromosomal datasets respectively.

### Microsatellite genotyping and data analyses

To investigate whether different types of genetic markers reveal the same population structure and genetic diversity, we extracted microsatellite genotype data from the sequencing reads. First, the software TandemRepeatsFinder^79^ was used to create a database of autosomal tandem repeats (microsatellites) present in the reference genome. Because read length imposes an upper limit to the length of microsatellites which can be reliably genotyped, and because tetranucleotides can be more reliably genotyped than dinucleotides, we selected tetranucleotide repeats with a repeat length of 7 to 9 units and an alignment score of 100, retaining 3007 microsatellites in total. We used the software bedtools^80^ to ascertain that the selected microsatellite loci did not overlap with the SNP data.

Second, we genotyped each individual for the 3007 selected loci, using the following approach. We used the software bedtools^80^ to extract from the bam files all reads which overlapped with one or more of the selected microsatellites. We used a custom Unix script to discard reads with a truncated microsatellite and to subsequently determine repeat lengths in the retained reads (Fig. S13). We used a custom R script for genotype scoring. In effect, alleles with a read depth below three were discarded. In cases where more than two alleles remained, the two best supported alleles were chosen. In cases where the second and third option alleles were supported by equal amounts of reads, the locus was scored homozygous for the best supported allele. The genotype data were analysed in R using functions of the R package adegenet^78^.

### Population structure analyses

The genetic distance between samples was estimated from whole genome data or alternatively from SNP data using either the allele sharing distance (ASD) and Euclidean distance (ED) metrics, and subsequently visualised using principal coordinates analyses (PCoA), a minimum spanning tree, and two hierarchical clustering methods (bioNJ and the ordinary least squares (OLS) version of the minimum evolution (ME) principle), by running the functions ‘pcoa’, ‘mst’, ‘bionj’ and ‘fastme.OLS’ of the R package ape-5.3^81^.

We established experimentally that bioNJ and OLS clustering of ASD and ED scores gives minimal discrepancy between the values in the distance matrix and the genetic distances suggested by the dendrogram (i.e., path length between tip pairs), thereby outperforming other combinations of distance metrics and hierarchical clustering methods (Fig. S14). The distance metrics ASD and ED are suitable for our purposes because 1.) they are free of assumptions regarding the underlying cause of observed differences (i.e., whether the observed differences originate from novel mutations or instead from genetic drift of standing variation) and 2.) are designed particularly for comparisons of individual genotypes instead of population allele frequencies.

### Admixture analyses

We used five methods to detect admixture. First, ancestry coefficients were calculated from the autosomal SNP dataset using the software Admixture^82^ (default settings) as well as the R package LEA-2.8.0^83^ (functions ‘snmf’ and ‘Q’, with alpha set to 10, tolerance to 0.00001, and number of iterations to 200). The optimal number of clusters (*K*) was determined using the elbow method on cross-entropy scores generated for *K* = 2 to *K* = 12 (with 50 independent runs each), with the assumption that the optimal K coincides with start point of a plateau.

Second, f3-statistics were generated with the software Admixtools^84^. The advantage of the f3-statistic compared to the D-statistic is that admixture signals can be reliably inferred without an established phylogeny. The f3-statistic is defined as (*a*-*b*)·(*a*-*c*), in which *a, b* and *c* represent vectors with the allele frequencies in the putatively admixed population A and the two putative donor populations B and C respectively. If, and only if, *a* is intermediate between *b* and *c*, will the product (*a*-*b*)(*a*-*c*) be negative. Hence, a negative f3-score is indicative of admixture. In the conjectural extreme scenario in which all alleles have been differentially fixed in the two ancestral populations, and in which the two populations contribute equally to the admixed population, the initial f3-value equals −0.25, as given by (0.5–1)·(0.5–0). In reality, the allele frequency differences between the two ancestral populations will be smaller, and the relative contributions unequal, resulting in f3-scores closer to zero.

We computed f3-scores for all possible population triplets (A;B,C), for non-overlapping windows with a fixed width of 50 kb. For 26 populations (excluding the population of American black bears), the total number of triplets is (26·25·24)/2 = 7800. From the window scores we derived the proportion of the genome with negative f3-scores, as well as the overall mean score. Because runs of homozygosity resulted in highly positive or highly negative f3-values, windows with f3-scores below −5 or above 5 were not included in the calculation of mean genome-wide values. We furthermore determined that discrepancy between the genome-wide average and the proportion of windows with a negative score could arise due to differences in the magnitude of f3-scores. For example, 59% of 50 kb windows of the triplet (Sakhalin; Amur, Hokkaido) had a negative f3-score, with an average value of −0.18. A minority of windows (41%) had a positive f3-score, but with an average value of 0.69. The resulting genome-wide f3-score was positive.

Third, we created admixture graphs using the software Treemix^43^, with default settings, using the autosomal SNP dataset as input. Fourth, we compared the path lengths between all sample pairs in the phylogeny (see section on population structure analyses) with the actual genetic distances in the distance matrix. We reasoned that a difference between genetic distance and path length might indicate violation of the assumption of a strictly bifurcating tree. Admixture events, which cause a node to have more than one parental node, will result in two lineages having lower or higher genetic distances than suggested by the path length in the tree (Figs. 1e, h and S2).

Fourth, we constructed a phylogenetic network following the neighbor-net algorithm^85^, using the ‘neighborNet’ function of the R package phangorn^86^, applied to a dataset of genome-wide uncorrected genetic distances (see section on genome-wide statistics). Fifth, we tested for the presence of quartets with unequal alternative quartet topology frequencies (see next section on multispecies coalescent analyses).

### Multispecies coalescent analyses

Multispecies coalescent (MSC) based analyses were performed on a dataset of 3075 haploblocks of a minimum 25 kb length, containing at minimum 50 biallelic sites with a minor allele frequency of 0.2 or higher. These haploblocks were detected using Plink (–blocks option), with the options ‘block-max-kb’ and ‘blocks-min-maf’ set to 1500 and 0.2, respectively. Linkage disequilibrium estimates within and between haploblocks were visually inspected using the software LDBlockShow^87^ (Fig. S15A). The rationale behind dividing the genome into haploblocks is to meet two key assumptions underlying MSC-based analyses: no recombination within loci, and no linkage between loci. The rationale behind selecting haploblocks with a certain minimum length and/or number of variable sites is to obtain sufficient phylogenetic signal per locus. The median number of variable sites per haploblock (regardless of minor allele frequency) was 462, with ~95% of haploblocks containing at least 300 variable sites (Fig. S15B).

All sites (monomorphic and polymorphic) within the boundaries of a haploblock were extracted from the vcf-file using bcftools view, and subsequently phased with the software Beagle version 5.4^88^, using default settings, resulting in a dataset of (135*2=) 270 haplotypes. The two haplotypes per individual were randomly labelled ‘1’ and ‘2’. For all 3075 haploblocks, matrices of uncorrected genetic distances between all (270*269)/2 = 36315 haplotype pairs were generated using custom-built Unix and R scripts, from which ‘biological neighbour-joining’ phylogenies were inferred using the function ‘bionj’ of the R-package ape. From these 3075 unrooted haploblock trees, a supertree was computed using the software Astral 5.7.8, with American black bears as outgroup.

The software Twisst was used to calculate quartet topology frequencies aggregated by population, for all (31 choose 4) 31,645 quartets. (For these analyses, the populations of Europe and Hokkaido were subdivided according to mtDNA clusters, resulting in 31 populations instead of 27 populations.) Quartet topology frequencies were summed over all 3075 loci, and depicted in a simplex plot^89^.

Quartet frequencies were defined as t1, t2 and t3, corresponding to, respectively, the species tree topology q1, the most frequent alternative topology q2, and the less frequent alternative topology q3. The species tree topology t1 was determined by calculating Robinson-Foulds distances between the three quartet topologies and a reference tree, using the ‘RF.dist’ function of the R package phangorn. The reference tree was generated through neighbour-joining clustering based on Nei’s genetic distances between populations (as inferred from the SNP dataset), using functions of the R packages StAMPP^90^ and phangorn.

The difference between t2 and t3 was quantified using the D-statistic, namely as: D = (t2–t3)/(t2 + t3). The null hypothesis of equal alternative topology frequencies (i.e., t2 ≈ t3) was tested by calculating z-scores, i.e., by expressing the mean (genome-wide) D-value in units of the standard deviation of chromosome specific D-values (sd). In formula: Z = D_mean/D_se, with D_se = D_sd/√n_chroms, with D_sd = sd(D_chrom) and n_chroms = 36. The null hypothesis was considered rejected when *z* ≥ 5.

To summarise the population composition of quartets with unequal alternative frequencies, we developed the ingroup-score, here defined as *n*_*t2*_/*n*_*alt*_. The numerator *n*_*t2*_ denotes the number of quartets for which populations *x* and *y* group together in q2 and for which the null hypothesis of equal frequencies (t2 ≈ t3) is rejected. The denominator *n*_*alt*_ is the total number of quartets in which populations *x* and *y* are both present but do not group together in q1. (The total number of quartets containing population pair x and y is the sum of *n*_*alt*_ and *n*_*t1*_, and equals: (npops–2) choose 2.)

### Geographical maps

Geographical maps depicting the sample distribution were generated using the function ‘map’ of the R package ‘maps’, using the ‘world’ database from the Natural Earth data set, which is in the public domain^91^. The shape files underlying the present-day and historic brown bear ranges were obtained from IUCN^5^ (https://www.iucn.redlist.org), and are freely available for non-commercial use.

#### Climate suitability modelling

The workflow underlying the climate suitability models is depicted in Supplementary Materials S4A. In short, to estimate the relationship between species occurrences and their climate characteristics we employed the maximum entropy species distribution model (MaxEnt)^92^. We trained the models through 5000 iterations of random sampling from the entire IUCN expert range map of the brown bear^5^, and 19 bioclimatic variables obtained from Chelsa-Climate^93^. In each run, the sampled occurrences were split; 50% were used to train the models and the remaining 50% were used to evaluate the performance of the models with their discriminatory power. We transferred the climate suitability models through time to the LGM using three PMIP3 Generalized Circulation Models; CCSM4, MIROC-ESM, and MPI-ESM-P^93^, as well as to late-Holocene Meghalayan, mid-Holocene Northgrippian, early-Holocene Greenlandian, Younger Dryas Stadial, Bølling-Allerød and Heinrich Stadial, all obtained from PaleoClim^94^. We performed all the geospatial analyses in R version 4.1.1^76^, using raster version 3.4-13^95^, rdgal version 1.5-23^96^ and dismo 1.3-3^97^.

### Forward-in-time genetic simulations

Forward-in-time genetic modelling simulations were performed using the software SLIM2^45^. To assess the effect of gene flow on population structure, we evaluated the dependency of various population differentiation metrics (i.e., Fst, f3, f4) on the duration (*t*) and the strength of continuing gene flow (*m*), or alternatively on the time (*t*) passed since a pulse-admixture event with varying admixture proportions (*a*). To assess the effect of effective population size (*Ne*) on genetic diversity, we evaluated the dependency of inbreeding statistics (i.e., the proportion and length of runs of homozygosity) on *t* and *Ne*, assuming a population size reduction at *t* = 0. Because of the absence of genotype errors, and the moderate size of the populations (Ne < 5000), the He-threshold for ROH-detection was set to 0.0025%, 20 times lower than for the empirical data.

All simulations were run on an autosomal region of 50 Mb in length, assuming a mutation rate (*µ*) of 1.2 × 10^−8^ per site per generation, a recombination rate of 1 × 10^−8^, and a fixed selection coefficient of 0 (i.e., neutral dynamics). Prior to the start of each demographic scenario, the ancestral population *p0* (*Ne* = 5000 or *Ne* = 10000) was allowed to evolve for 25000 generations. We established experimentally that mutation-drift equilibrium (i.e., *He* = 4·*Ne*·*µ*) was reached within this time frame. SLIM2 allows to specify census population size instead of effective population size, and hence our *Ne*-estimates were in fact upper boundaries of the true effective population size. Multi-allelic SNPs were filtered out from the output vcf-files. All subsequent analyses were performed on a sample size of 10 individuals per population.

### Reporting summary

Further information on research design is available in the Nature Portfolio Reporting Summary linked to this article.

## Supplementary information

Supplementary Material

Reporting Summary

## Data Availability

All analysis outcomes needed to evaluate the conclusions in the paper are presented in the paper and/or the Supplementary Materials. Raw sequencing data is available from the NCBI’s SRA repository, bioproject-ID PRJNA913591 (biosample accessions: SAMN32301302 - SAMN32301397, Table S1). SNP and microsatellite datasets, along with instructions to reproduce the main figures, have been uploaded to the Dryad repository (10.5061/dryad.qbzkh18n6), under the title: ‘Range-wide whole-genome resequencing of the brown bear reveals drivers of intraspecies divergence’.

## Code availability

Scripts for mapping and genotype calling are available from: https://github.com/mennodejong1986/Fastq2VCF. Scripts for microsatellite genotyping from short read sequencing data are available from: https://github.com/mennodejong1986/MicrosatelliteGenotypingFromBam. The software Darwindow, used for genome-wide sliding-window analyses (i.e., He, ROH, uncorrected genetic distance, and Dxy analyses), is available from: https://github.com/mennodejong1986/Darwindow. Script used for calculation of genome-wide uncorrected genetic distances for all sample pairs, assuming the data is in VCF-format, is available from: https://github.com/mennodejong1986/VCF_pairwise_distances. Scripts for haploblock detection and subsequent multispecies coalescent based analyses (testing for inequality of alternative quartet topology frequencies) are available from: https://github.com/mennodejong1986/PopMSC. A full detailed report on the discrimination power and accuracy measure of the climate suitability models, along with underlying R scripts, is available from: 10.5281/zenodo.6628939.
