# Supplementary material for: Range-wide whole-genome resequencing of the brown bear reveals drivers of intraspecies divergence
Source: Commun Biol. 2023 Feb 6;6:153. doi: 10.1038/s42003-023-04514-w (PMC9902616; doi:10.1038/s42003-023-04514-w)
Supplement: Supplementary file 1 — Supplementary Material [file 42003_2023_4514_MOESM1_ESM.pdf]

SUPPLEMENTARY MATERIAL

Range-wide whole-genome resequencing of the brown bear reveals drivers of intraspecies divergence

AUTHORS AND AFFILIATIONS

Menno J. de Jong[1*], Aidin Niamir[1], Magnus Wolf[1,2], Andrew C. Kitchener[3,4], Nicolas Lecomte[5], Ivan V. Seryodkin[6], Steve Fain[7], Snorre B. Hagen[8], Urmas Saarma[9], Axel Janke[1,2,10]

* corresponding author. Email to: menno.de-jong@senckenberg.de

1. Senckenberg Biodiversity and Climate Research Institute (SBiK-F), Georg-Voigt-Strasse 14-16, Frankfurt am Main, 60325, Germany.

2. Institute for Ecology, Evolution and Diversity, Goethe University, Max-von-Laue-Strasse. 9, Frankfurt am Main, Germany.

3. Department of Natural Sciences, National Museums Scotland, Chambers Street, Edinburgh EH1 1JF, UK.

4. School of Geosciences, University of Edinburgh, Drummond Street, Edinburgh EH8 9XP, UK

5. Canada Research Chair in Polar and Boreal Ecology, Department of Biology, University of Moncton, Moncton, New Brunswick, E1H1R2, Canada.

6. Pacific Geographical Institute of the Far Eastern Branch of the Russian Academy of Sciences, 7 Radio St., Vladivostok, 690041, Russia.

7. National Fish & Wildlife Forensic Laboratory, Ashland, OR, USA.

8. Norwegian Institute of Bioeconomy Research, Division of Environment and Natural Resources, Svanhovd, N-9925, Svanvik, Norway.

9. Department of Zoology, Institute of Ecology and Earth Sciences, University of Tartu, J. Liivi 2, Tartu 50409, Estonia.

10. LOEWE-Centre for Translational Biodiversity Genomics (TBG), Senckenberg Nature Research Society, Georg-Voigt-Strasse 14-16, Frankfurt am Main, Germany.

TABLE OF CONTENTS

Table S1. Sample list.

Table S2. Data underlying figure 4c.

Table S3. Data underlying figure 4e.

Table S4. Data underlying figure 4f.

Table S5. Data underlying figure 6f.

Fig. S1A. Mean sequencing depth per sample.

Fig. S1B. Distribution of read depth across sites along the nuclear genome.

Fig. S1C. Relatedness between individuals.

Fig. S2A. PCoA-plot showing genetic structure in western Eurasia.

Fig. S2B. OLS dendrogram of Euclidean distances.

Fig. S2C. Discrepancy between path lengths and genetic distance.

Fig. S2D. Microsatellite dataset bioNJ dendrogram.

Fig. S3A. Mitogenome maximum likelihood phylogeny.

Fig. S3B. Mitogenome maximum likelihood phylogeny (clade 3a).

Fig. S4A. Climate suitability modelling (SDM) flowchart.

Fig. S4B. Climate suitability modelling (SDM), present projection.

Fig. S5A. Admixtools f3-statistics.

Fig. S5B. Admixtools f3-statistics: mean vs sd.

Fig. S5C. Treemix maximum likelihood phylogenies with 0 to 8 migration edges.

Fig. S6A. Determination of optimal K using cross-entropy score criterion.

Fig. S6B-D. Admixture plots for X-chromosomal and autosomal datasets.

Fig. S7A. Genome-wide heterozygosity (He); Darwindow versus bcftools.

Fig. S7B. Genome-wide heterozygosity (He) before and exclusion of ROHs.

Fig. S8. Heterozygosity levels within ROHs.

Fig. S9. Sex determination based on levels of missing data at the Y-chromosome.

Fig. S10A-C. Heterozygosity as a function of read depth and bcftools call settings.

Fig. S11. Genome wide heterozygosity as a function of bcftools call 'snpGap' setting.

Fig. S12. Overall depth per site.

Fig. S13A-C. Microsatellite (VNTR) genotyping from short read sequencing data.

Fig. S14A-B. Accuracy of hierarchical clustering methods.

Fig. S15A. Haploblock detection.

Fig. S15B. Number of variable (i.e., polymorphic) sites per haploblock.

Table S1. Sample list. List of brown bears samples sequenced for this study or retrieved from the NCBI SRA repository.

name	population	colour	latitude	longitude	authors	NCBI_ID
ABC1	ABCbc	blue4	56.9508	-134.942	Liu et al. 2014	SAMN02256316
ABC10	ABCa	mediumblue	57.44	-134.2	Cahill et al. 2014	SAMN02045560
ABC11	ABCbc	blue4	57.87083	-135.772	Cahill et al. 2014	SAMN03247209
ABC12	ABCbc	blue4	57.87083	-135.772	Cahill et al. 2014	SAMN03252406
ABC13	ABCa	mediumblue	57.44	-134.2	Miller at al. 2012	SAMN01057688
ABC14	ABCbc	blue4	56.95083	-134.942	Miller et al. 2012	SAMN01057689
ABC15	ABCcoast2	darkorchid2	57.98417	-133.788	this_study	SAMN32301302
ABC16	ABCcoast2	darkorchid2	57.98417	-133.788	this_study	SAMN32301303
ABC2	ABCbc	blue4	56.9508	-134.942	Liu et al. 2014	SAMN02256317
ABC3	ABCbc	blue4	57.87083	-135.772	Liu et al. 2014	SAMN02256318
ABC4	ABCbc	blue4	57.87083	-135.772	Liu et al. 2014	SAMN02256319
ABC5	ABCbc	blue4	57.87083	-135.772	Liu et al. 2014	SAMN02256320
ABC6	ABCa	mediumblue	57.73333	-134.333	Liu et al. 2014	SAMN02256321
ABC7	ABCa	mediumblue	57.73333	-134.333	this_study	SAMN32301304
ABC8	ABCa	mediumblue	57.73333	-134.333	this_study	SAMN32301305
ABC9	ABCa	mediumblue	57.73333	-134.333	this_study	SAMN32301306
Alaska1	Kodiak	lightskyblue3	57.79	-152.407	this_study	SAMN32301307
Alaska11	Alaska	steelblue3	68.143	-151.736	this_study	SAMN32301308
Alaska12	Alaska	steelblue3	68.143	-151.736	this_study	SAMN32301309
Alaska2	Alaska	steelblue3	60.55444	-151.258	Miller et al. 2012	SAMN01057690
Alaska3	Alaska	steelblue3	63.12989	-151.197	Cahill et al. 2013	SAMN02045559
Alaska7	Alaska	steelblue3	68.143	-151.736	this_study	SAMN32301310
Alaska9	Alaska	steelblue3	68.143	-151.736	this_study	SAMN32301311
AlaskaN1	Alaska	steelblue3	70.29	-148.79	this_study	SAMN32301397
AlaskaSW1	Aleutian	deepskyblue	55.328	-160.5	this_study	SAMN32301393
AlaskaSW2	Aleutian	deepskyblue	55.81	-166.66	this_study	SAMN32301394
AlaskaSW3	Aleutian	deepskyblue	55.81	-166.66	this_study	SAMN32301395
AlaskaW1	Alaska	steelblue3	66.94	-160.6	this_study	SAMN32301396
Americanblack1	Black	black	53	-121	Cahill et al. 2013	SAMN02045561
Americanblack2	Black	black	53	-121	Miller et al. 2012	SAMN01057691
Amur1	Amur	darkgreen	52.43055	140.3289	this_study	SAMN32301312
Amur2	Amur	darkgreen	46.0321	136.677	this_study	SAMN32301313
Amur3	Amur	darkgreen	45.4562	137.185	this_study	SAMN32301314
Amur4	Sakhalin	darkcyan	48	142.2532	this_study	SAMN32301315
Amur5	Yakutia	limegreen	57.645	136.23	this_study	SAMN32301316
Canada1	Westcoast	mediumpurple2	49.93	-117.627	this_study	SAMN32301317
Canada10	HudsonBay	darkorchid4	61.117	-94.05	this_study	SAMN32301318
Canada11	HudsonBay	darkorchid4	61.117	-94.05	this_study	SAMN32301319
Canada12	HudsonBay	darkorchid4	61.117	-94.05	this_study	SAMN32301320
Canada2	HudsonBay	darkorchid4	62.2373	-92.5922	this_study	SAMN32301321
Canada3	HudsonBay	darkorchid4	61.117	-94.05	this_study	SAMN32301322

Canada4	HudsonBay	darkorchid4	61.117	-94.05	this_study	SAMN32301323
Canada5	HudsonBay	darkorchid4	64.167	-95.5	this_study	SAMN32301324
Canada6	HudsonBay	darkorchid4	64.167	-95.5	this_study	SAMN32301325
Canada7	HudsonBay	darkorchid4	62.75	-92	this_study	SAMN32301326
Canada8	HudsonBay	darkorchid4	61.117	-94.05	this_study	SAMN32301327
Canada9	HudsonBay	darkorchid4	61.117	-94.05	this_study	SAMN32301328
CentralRussia1	Ural	yellow2	55.5	80.5	this_study	SAMN32301329
CentralRussia2	CentreRus2	bisque3	55.5	91.4	this_study	SAMN32301330
CentralRussia3	CentreRus	greenyellow	55.4	91.4	this_study	SAMN32301331
CentralRussia4	CentreRus	greenyellow	60.3	94.3	this_study	SAMN32301332
CentralRussia5	Ural	yellow2	57.6	57.6	this_study	SAMN32301333
Estonia1	Baltic	gold3	59.008	26.226	this_study	SAMN32301334
Estonia2	Baltic	gold3	59.2672	27.552	this_study	SAMN32301335
FarEast1	Magadan	aquamarine2	59.5638	150.8035	this_study	SAMN32301336
FarEast10	Magadan	aquamarine2	59.6	153.15	this_study	SAMN32301344
FarEast11	Magadan	aquamarine2	59.6	153.15	this_study	SAMN32301345
FarEast2	Magadan	aquamarine2	59.5638	150.8035	this_study	SAMN32301337
FarEast3	Magadan	aquamarine2	59.5638	150.8035	this_study	SAMN32301338
FarEast4	Magadan	aquamarine2	59.5638	150.8035	this_study	SAMN32301339
FarEast6	Yakutia	limegreen	56.65	124.7	this_study	SAMN32301340
FarEast7	Yakutia	limegreen	64.31348	133.5363	this_study	SAMN32301341
FarEast8	Yakutia	limegreen	60.02505	123.3926	this_study	SAMN32301342
FarEast9	Yakutia	limegreen	64.16806	116.4672	this_study	SAMN32301343
Finland	Baltic	gold3	61.28333	28.83333	Liu et al. 2014	SAMN02256315
Finland2	Baltic	gold3	61.6934	29.729	this_study	SAMN32301346
Finland3	Baltic	gold3	61.91971	27.81635	this_study	SAMN32301347
Georgia	MiddleEast	grey50	43.02592	43.04349	Barlow et al. 2018	SAMEA4762871
Greece1	Europe	darkred	39.07421	21.82431	Benazo et al. 2018	SAMN07422268
Himalaya1*	Himalaya	grey20	38.11	70.41	this_study	SAMN32301348
Hokkaido1	Hokkaido	darkcyan	42.9239	143.1961	this_study	SAMN32301349
HokkaidoCentral1	Hokkaido	darkcyan	43.317	141.967	Endo et al. 2021	DRR276776
HokkaidoCentral2	Hokkaido	darkcyan	45.092	141.831	Endo et al. 2021	DRR276777
HokkaidoEast1	Hokkaido	darkcyan	43.728	144.587	Endo et al. 2021	DRR276778
HokkaidoEast2	Hokkaido	darkcyan	43.987	144.952	Endo et al. 2021	DRR276779
HokkaidoSouth1	Hokkaido	darkcyan	41.729	140.4298	Endo et al. 2021	DRR276774
HokkaidoSouth2	Hokkaido	darkcyan	42.942	140.617	Endo et al. 2021	DRR276775
IranGudrun*	MiddleEast	grey50	38	46	this_study	SAMN32301350
Italy1	Europe	darkred	42.03908	13.43847	Benazo et al. 2017	SAMN07422262
Kamtschatka3	Kamtchatka	cyan2	56.21095	159.3467	this_study	SAMN32301351
Kamtschatka4	Kamtchatka	cyan2	56.21095	159.3467	this_study	SAMN32301352
Kirkenes	NorthScand	orange	69.72706	30.04578	this_study	SAMN32301362
Kodiak1	Kodiak	lightskyblue3	57.79	-152.407	this_study	SAMN32301353
Kodiak2	Kodiak	lightskyblue3	57.79	-152.407	this_study	SAMN32301354
Kodiak3	Kodiak	lightskyblue3	57.79	-152.407	this_study	SAMN32301355
Kodiak4	Kodiak	lightskyblue3	57.79	-152.407	this_study	SAMN32301356
Kodiak5	Kodiak	lightskyblue3	57.79	-152.407	this_study	SAMN32301357

Kodiak6	Kodiak	lightskyblue3	57.79	-152.407	this_study	SAMN32301392
Montana	Westcoast	mediumpurple2	46.96526	-109.534	Liu et al. 2014	SAMN02256322
NorthSweden	MidScand	indianred1	66.60665	19.82324	Liu et al. 2014	SAMN02256314
Norway1***	NorthScand	orange	69.96887	23.27165	this_study	SAMN32301358
Norway2***	NorthScand	orange	69.96887	23.27165	this_study	SAMN32301359
Norway3	NorthScand	orange	69.4439	25.80482	this_study	SAMN32301360
Norway4	NorthScand	orange	69.4439	25.80482	this_study	SAMN32301361
Norway6	NorthScand	orange	70.483	26.0135	this_study	SAMN32301363
Norway7	MidScand	orange	70.483	26.0135	this_study	SAMN32301364
Norway8	NorthScand	orange	70.483	26.0135	this_study	SAMN32301365
polar1	polar	blue	70.78	-22.91	Liu et al. 2014	SAMN02231736
polar4	polar	blue	70.78	-22.91	Liu et al. 2014	SAMN02231733
polarSvalbard1	polar	blue	79	17.6	Miller et al. 2012	SAMN01057666
polarSvalbard2	polar	blue	79	17.6	Miller et al. 2012	SAMN01057636
Rumania1	Europe	darkred	44.43996	26.09631	this_study	SAMN32301366
Rumania2	Europe	darkred	44.43996	26.09631	this_study	SAMN32301367
Rumania3	Europe	darkred	44.43996	26.09631	this_study	SAMN32301368
Rumania4	Europe	darkred	44.43996	26.09631	this_study	SAMN32301369
Rumania5	Europe	darkred	44.43996	26.09631	this_study	SAMN32301370
Russia	CentreRus	greenyellow	55.23	91.37	Barlow et al. 2018	SAMEA4762872
Russia_Kola1	NorthScand	orange	68.979	33.09	this_study	SAMN32301371
Russia_Kola3	NorthScand	orange	68.979	33.09	this_study	SAMN32301372
Slovakia1	Europe	darkred	48.14816	17.10674	Benazo et al. 2017	SAMN07422269
Slovenia	Europe	darkred	46.445	14.01778	Barlow et al. 2018	SAMEA4762870
Slovenia1	Europe	darkred	46.15124	14.99546	Benazo et al. 2017	SAMN07422272
SouthNorway1	SouthScand	orangered3	60.83333	11.6666	this_study	SAMN32301373
SouthNorway2	SouthScand	orangered3	60.83333	11.6666	this_study	SAMN32301374
SouthNorway3	SouthScand	orangered3	60.83333	11.6666	this_study	SAMN32301375
SouthNorway4	MidScand	indianred1	63.16666	10.3333	this_study	SAMN32301376
SouthNorway5	SouthScand	orangered3	63.16666	10.3333	this_study	SAMN32301377
SouthNorway6	MidScand	indianred1	63.16666	10.3333	this_study	SAMN32301378
SouthNorway7	MidScand	indianred1	63.16666	10.3333	this_study	SAMN32301379
SouthSweden	SouthScand	orangered3	62.73334	17.58332	Liu et al. 2014	SAMN02256313
Spain1	Europe	darkred	43.46278	-3.805	Benazo et al. 2017	SAMN07422271
Sweden3	SouthScand	orangered3	61.00487	14.53714	Cahill et al. 2014	SAMN03252407
TorontoZoo**	ABCcoast1	darkorchid2	58.747	-134.878	Taylor et al. 2018	SAMN09907428
TurkeyMartin*	MiddleEast	grey50	39.9	41.3	this_study	SAMN32301380
Ural1	Ural	yellow2	58	56.31667	this_study	SAMN32301381
Ural2	Ural	yellow2	58	56.31667	this_study	SAMN32301382
Ural3	Ural	yellow2	58	56.31667	this_study	SAMN32301383
Ural4	Ural	yellow2	58	56.31667	this_study	SAMN32301384
Ural5	Ural	yellow2	60.3262	56.423	this_study	SAMN32301385
Ural6	Ural	yellow2	63.3162	42.533	this_study	SAMN32301386
US1	Westcoast	mediumpurple2	44.0682	-114.742	this_study	SAMN32301387
US2	Westcoast	mediumpurple2	48.1978	-114.316	this_study	SAMN32301388
US3	Westcoast	mediumpurple2	44.03971	-109.541	this_study	SAMN32301389

US6	Westcoast	mediumpurple2	48.8	-117.255	this_study	SAMN32301390
US8	Westcoast	mediumpurple2	44.56	-111.444	this_study	SAMN32301391

*born in zoo; **orphaned in the wild, relocated to zoo; ***duplex sequenced sample

Table S2. Data underlying figure 4c. Data underlying figure 4c, showing population pairwise Fst-values, calculated with the software vcftools, for an in-silico meta-population experiencing a change in population connectivity, simulated with the software SLIM2. See main text for more details.

population_pairs	population_pairs_short	fst	fst_corrected	n_generations	m	Ne
NorthWest_SouthWest	p12_p11	-0.0016644	-0.0008173	0	0.001	10000
NorthEast_NorthWest	p22_p12	0.029999	0.048961	0	0.001	10000
SouthEast_SouthWest	p12_p11	0.028862	0.046916	0	0.001	10000
SouthEast_NorthEast	p12_p22	-0.001793	-0.00060982	0	0.001	10000
NorthWest_SouthEast	p12_p12	0.029791	0.048799	0	0.001	10000
NorthEast_SouthWest	p22_p11	0.029125	0.047257	0	0.001	10000
NorthWest_SouthWest	p12_p11	0.01507	0.026538	1000	0.001	10000
NorthEast_NorthWest	p22_p12	0.0062002	0.011513	1000	0.001	10000
SouthEast_SouthWest	p12_p11	0.0049871	0.0092058	1000	0.001	10000
SouthEast_NorthEast	p12_p22	0.0049691	0.0089826	1000	0.001	10000
NorthWest_SouthEast	p12_p12	0.011516	0.02058	1000	0.001	10000
NorthEast_SouthWest	p22_p11	0.01058	0.017879	1000	0.001	10000
NorthWest_SouthWest	p12_p11	0.002202	0.0047807	100	0.001	10000
NorthEast_NorthWest	p22_p12	0.018268	0.030495	100	0.001	10000
SouthEast_SouthWest	p12_p11	0.021742	0.036826	100	0.001	10000
SouthEast_NorthEast	p12_p22	0.0011341	0.0035982	100	0.001	10000
NorthWest_SouthEast	p12_p12	0.02213	0.037243	100	0.001	10000
NorthEast_SouthWest	p22_p11	0.018247	0.030421	100	0.001	10000
NorthWest_SouthWest	p12_p11	0.019845	0.033569	1500	0.001	10000
NorthEast_NorthWest	p22_p12	0.0064325	0.011659	1500	0.001	10000
SouthEast_SouthWest	p12_p11	0.0060963	0.011017	1500	0.001	10000
SouthEast_NorthEast	p12_p22	0.0050276	0.0095231	1500	0.001	10000
NorthWest_SouthEast	p12_p12	0.012754	0.021837	1500	0.001	10000
NorthEast_SouthWest	p22_p11	0.013364	0.023313	1500	0.001	10000
NorthWest_SouthWest	p12_p11	0.019043	0.033201	2000	0.001	10000
NorthEast_NorthWest	p22_p12	0.0074276	0.013559	2000	0.001	10000
SouthEast_SouthWest	p12_p11	0.0060433	0.011254	2000	0.001	10000
SouthEast_NorthEast	p12_p22	0.006488	0.012245	2000	0.001	10000
NorthWest_SouthEast	p12_p12	0.013117	0.02335	2000	0.001	10000
NorthEast_SouthWest	p22_p11	0.013541	0.024474	2000	0.001	10000
NorthWest_SouthWest	p12_p11	0.0035093	0.0073145	200	0.001	10000
NorthEast_NorthWest	p22_p12	0.011658	0.020644	200	0.001	10000
SouthEast_SouthWest	p12_p11	0.012721	0.02195	200	0.001	10000
SouthEast_NorthEast	p12_p22	0.00088776	0.0031812	200	0.001	10000
NorthWest_SouthEast	p12_p12	0.013713	0.023457	200	0.001	10000
NorthEast_SouthWest	p22_p11	0.012933	0.022197	200	0.001	10000
NorthWest_SouthWest	p12_p11	-0.0005961	0.00096306	20	0.001	10000
NorthEast_NorthWest	p22_p12	0.027795	0.0458	20	0.001	10000
SouthEast_SouthWest	p12_p11	0.027381	0.046823	20	0.001	10000
SouthEast_NorthEast	p12_p22	-0.0028602	-0.0015891	20	0.001	10000
NorthWest_SouthEast	p12_p12	0.026455	0.044987	20	0.001	10000
NorthEast_SouthWest	p22_p11	0.029095	0.048523	20	0.001	10000
NorthWest_SouthWest	p12_p11	0.0077611	0.0136	300	0.001	10000
NorthEast_NorthWest	p22_p12	0.015705	0.026662	300	0.001	10000
SouthEast_SouthWest	p12_p11	0.010954	0.018555	300	0.001	10000
SouthEast_NorthEast	p12_p22	0.0050161	0.0087127	300	0.001	10000
NorthWest_SouthEast	p12_p12	0.013357	0.022802	300	0.001	10000
NorthEast_SouthWest	p22_p11	0.017273	0.028885	300	0.001	10000
NorthWest_SouthWest	p12_p11	0.0071991	0.012589	400	0.001	10000
NorthEast_NorthWest	p22_p12	0.010453	0.017996	400	0.001	10000
SouthEast_SouthWest	p12_p11	0.0084148	0.015419	400	0.001	10000
SouthEast_NorthEast	p12_p22	0.0058376	0.010823	400	0.001	10000
NorthWest_SouthEast	p12_p12	0.011401	0.01919	400	0.001	10000
NorthEast_SouthWest	p22_p11	0.012234	0.021661	400	0.001	10000
NorthWest_SouthWest	p12_p11	-0.0004584	0.0019153	50	0.001	10000
NorthEast_NorthWest	p22_p12	0.026455	0.045255	50	0.001	10000
SouthEast_SouthWest	p12_p11	0.024897	0.042048	50	0.001	10000
SouthEast_NorthEast	p12_p22	0.00017428	0.0022829	50	0.001	10000
NorthWest_SouthEast	p12_p12	0.026922	0.045849	50	0.001	10000

NorthEast_SouthWest	p22_p11	0.024634	0.042315	50	0.001	10000
NorthWest_SouthWest	p12_p11	0.01221	0.020687	600	0.001	10000
NorthEast_NorthWest	p22_p12	0.0083325	0.015382	600	0.001	10000
SouthEast_SouthWest	p12_p11	0.009134	0.01615	600	0.001	10000
SouthEast_NorthEast	p12_p22	0.006187	0.011148	600	0.001	10000
NorthWest_SouthEast	p12_p12	0.011604	0.020378	600	0.001	10000
NorthEast_SouthWest	p22_p11	0.012565	0.021939	600	0.001	10000
NorthWest_SouthWest	p12_p11	0.013253	0.022443	800	0.001	10000
NorthEast_NorthWest	p22_p12	0.007449	0.013929	800	0.001	10000
SouthEast_SouthWest	p12_p11	0.0048254	0.0091897	800	0.001	10000
SouthEast_NorthEast	p12_p22	0.004275	0.0082423	800	0.001	10000
NorthWest_SouthEast	p12_p12	0.0096692	0.016848	800	0.001	10000
NorthEast_SouthWest	p22_p11	0.011015	0.018793	800	0.001	10000
NorthWest_SouthWest	p12_p11	0.0013957	0.0044363	0	0.01	10000
NorthEast_NorthWest	p22_p12	0.029619	0.048977	0	0.01	10000
SouthEast_SouthWest	p12_p11	0.02937	0.048575	0	0.01	10000
SouthEast_NorthEast	p12_p22	0.00023757	0.0024598	0	0.01	10000
NorthWest_SouthEast	p12_p12	0.028719	0.046839	0	0.01	10000
NorthEast_SouthWest	p22_p11	0.030191	0.050079	0	0.01	10000
NorthWest_SouthWest	p12_p11	0.00057653	0.0025494	1000	0.01	10000
NorthEast_NorthWest	p22_p12	-0.0017011	-0.0010384	1000	0.01	10000
SouthEast_SouthWest	p12_p11	-0.0010633	0.00011376	1000	0.01	10000
SouthEast_NorthEast	p12_p22	-0.0016206	-0.0011041	1000	0.01	10000
NorthWest_SouthEast	p12_p12	0.00040848	0.0022437	1000	0.01	10000
NorthEast_SouthWest	p22_p11	-0.0006624	0.0002275	1000	0.01	10000
NorthWest_SouthWest	p12_p11	0.0002325	0.0021496	100	0.01	10000
NorthEast_NorthWest	p22_p12	-4.51E-05	0.0014435	100	0.01	10000
SouthEast_SouthWest	p12_p11	0.001553	0.0038886	100	0.01	10000
SouthEast_NorthEast	p12_p22	-0.0004668	0.00102	100	0.01	10000
NorthWest_SouthEast	p12_p12	0.000885	0.0026248	100	0.01	10000
NorthEast_SouthWest	p22_p11	0.0012427	0.0035396	100	0.01	10000
NorthWest_SouthWest	p12_p11	-3.11E-05	0.0015828	1500	0.01	10000
NorthEast_NorthWest	p22_p12	0.00052334	0.003069	1500	0.01	10000
SouthEast_SouthWest	p12_p11	-0.0018978	-0.0012037	1500	0.01	10000
SouthEast_NorthEast	p12_p22	-2.22E-05	0.0022964	1500	0.01	10000
NorthWest_SouthEast	p12_p12	0.0001769	0.0020727	1500	0.01	10000
NorthEast_SouthWest	p22_p11	5.97E-05	0.002088	1500	0.01	10000
NorthWest_SouthWest	p12_p11	0.0023675	0.004793	2000	0.01	10000
NorthEast_NorthWest	p22_p12	-0.0008681	0.00057816	2000	0.01	10000
SouthEast_SouthWest	p12_p11	0.00034741	0.0019184	2000	0.01	10000
SouthEast_NorthEast	p12_p22	0.00071127	0.0025843	2000	0.01	10000
NorthWest_SouthEast	p12_p12	-2.91E-05	0.0012685	2000	0.01	10000
NorthEast_SouthWest	p22_p11	0.0021083	0.0048286	2000	0.01	10000
NorthWest_SouthWest	p12_p11	0.00079274	0.0033716	200	0.01	10000
NorthEast_NorthWest	p22_p12	-0.0009949	0.0002151	200	0.01	10000
SouthEast_SouthWest	p12_p11	-0.0009886	0.00070781	200	0.01	10000
SouthEast_NorthEast	p12_p22	-0.0002781	0.0014571	200	0.01	10000
NorthWest_SouthEast	p12_p12	-0.001086	9.29E-05	200	0.01	10000
NorthEast_SouthWest	p22_p11	0.00036658	0.0028135	200	0.01	10000
NorthWest_SouthWest	p12_p11	-0.0019592	-0.0016907	20	0.01	10000
NorthEast_NorthWest	p22_p12	0.012074	0.020113	20	0.01	10000
SouthEast_SouthWest	p12_p11	0.01193	0.019335	20	0.01	10000
SouthEast_NorthEast	p12_p22	0.00095022	0.0027496	20	0.01	10000
NorthWest_SouthEast	p12_p12	0.0090834	0.015517	20	0.01	10000
NorthEast_SouthWest	p22_p11	0.014811	0.023801	20	0.01	10000
NorthWest_SouthWest	p12_p11	0.00050635	0.0023167	300	0.01	10000
NorthEast_NorthWest	p22_p12	-0.0013465	-0.00024724	300	0.01	10000
SouthEast_SouthWest	p12_p11	-0.0020219	-0.0014258	300	0.01	10000
SouthEast_NorthEast	p12_p22	-0.0029416	-0.0023168	300	0.01	10000
NorthWest_SouthEast	p12_p12	-0.000931	-0.00011508	300	0.01	10000
NorthEast_SouthWest	p22_p11	-6.82E-05	0.0021087	300	0.01	10000
NorthWest_SouthWest	p12_p11	0.0010859	0.0033629	400	0.01	10000
NorthEast_NorthWest	p22_p12	-0.0015867	-0.00076629	400	0.01	10000
SouthEast_SouthWest	p12_p11	-0.0005163	0.0015396	400	0.01	10000
SouthEast_NorthEast	p12_p22	-0.0002643	0.0017003	400	0.01	10000
NorthWest_SouthEast	p12_p12	3.47E-05	0.0018928	400	0.01	10000
NorthEast_SouthWest	p22_p11	0.00020902	0.0019565	400	0.01	10000
NorthWest_SouthWest	p12_p11	0.00062478	0.002468	50	0.01	10000
NorthEast_NorthWest	p22_p12	0.0048614	0.009107	50	0.01	10000
SouthEast_SouthWest	p12_p11	0.005961	0.011625	50	0.01	10000
SouthEast_NorthEast	p12_p22	-0.0005366	0.0011799	50	0.01	10000

NorthWest_SouthEast	p12_p12	0.0042014	0.0073787	50	0.01	10000
NorthEast_SouthWest	p22_p11	0.0063389	0.011788	50	0.01	10000
NorthWest_SouthWest	p12_p11	0.0018117	0.004439	600	0.01	10000
NorthEast_NorthWest	p22_p12	0.00034797	0.0021728	600	0.01	10000
SouthEast_SouthWest	p12_p11	-0.0002872	0.0016415	600	0.01	10000
SouthEast_NorthEast	p12_p22	-0.0007988	0.00023739	600	0.01	10000
NorthWest_SouthEast	p12_p12	0.000342	0.0017299	600	0.01	10000
NorthEast_SouthWest	p22_p11	0.00022503	0.0021391	600	0.01	10000
NorthWest_SouthWest	p12_p11	0.0025582	0.0052721	800	0.01	10000
NorthEast_NorthWest	p22_p12	0.00063412	0.0028843	800	0.01	10000
SouthEast_SouthWest	p12_p11	0.001163	0.0037795	800	0.01	10000
SouthEast_NorthEast	p12_p22	0.0007615	0.0032794	800	0.01	10000
NorthWest_SouthEast	p12_p12	0.00079553	0.0026129	800	0.01	10000
NorthEast_SouthWest	p22_p11	0.001432	0.003658	800	0.01	10000

Table S3. Data underlying figure 4e. Data underlying figure 4e, showing f3-scores for population triplets, calculated with the software admixtools, for an in-silico stepping-stone meta-population, simulated with the software SLIM2. See main text for more details.

source1	source2	target	f3	std_err	Z	n_snps	n_generations	Ne	admix_prop
p1	p2	p3	0.048315	0.001911	25.288	77958	0	5000	0.001
p1	p2	p4	0.055982	0.002116	26.455	77758	0	5000	0.001
p1	p2	p5	0.043554	0.001899	22.931	77938	0	5000	0.001
p2	p3	p1	0.046452	0.002002	23.198	77958	0	5000	0.001
p2	p3	p4	0.054757	0.002071	26.441	77862	0	5000	0.001
p2	p3	p5	0.045012	0.001813	24.831	78091	0	5000	0.001
p1	p4	p2	0.043544	0.001936	22.492	77758	0	5000	0.001
p1	p4	p3	0.047107	0.001956	24.088	78307	0	5000	0.001
p1	p5	p2	0.043677	0.001884	23.18	77938	0	5000	0.001
p1	p5	p3	0.04976	0.001951	25.506	78554	0	5000	0.001
p1	p5	p4	0.055043	0.002078	26.489	78272	0	5000	0.001
p2	p3	p1	0.046452	0.002002	23.198	77958	0	5000	0.001
p2	p3	p4	0.054757	0.002071	26.441	77862	0	5000	0.001
p2	p3	p5	0.045012	0.001813	24.831	78091	0	5000	0.001
p2	p4	p1	0.043308	0.002076	20.856	77758	0	5000	0.001
p2	p4	p3	0.043988	0.001923	22.876	77862	0	5000	0.001
p2	p4	p5	0.042621	0.001859	22.926	77821	0	5000	0.001
p2	p5	p1	0.043175	0.00191	22.608	77938	0	5000	0.001
p2	p5	p3	0.046508	0.001957	23.766	78091	0	5000	0.001
p2	p5	p4	0.054908	0.002027	27.087	77821	0	5000	0.001
p3	p4	p1	0.047669	0.002089	22.818	78307	0	5000	0.001
p3	p4	p2	0.044751	0.001979	22.607	77862	0	5000	0.001
p3	p4	p5	0.045163	0.001851	24.395	77737	0	5000	0.001
p3	p5	p1	0.044995	0.002016	22.324	78554	0	5000	0.001
p3	p5	p2	0.042233	0.00195	21.66	78091	0	5000	0.001
p3	p5	p4	0.052351	0.002072	25.267	77737	0	5000	0.001
p4	p5	p1	0.044241	0.002001	22.115	78272	0	5000	0.001
p4	p5	p2	0.044601	0.001924	23.177	77821	0	5000	0.001
p4	p5	p3	0.046359	0.001977	23.453	77737	0	5000	0.001
p1	p2	p3	0.028106	0.001237	22.714	77468	1000	5000	0.001
p1	p2	p4	0.050597	0.001733	29.198	80748	1000	5000	0.001
p1	p2	p5	0.067868	0.002039	33.285	80667	1000	5000	0.001
p2	p3	p1	0.02099	0.001158	18.123	77468	1000	5000	0.001
p2	p3	p4	0.027788	0.001282	21.677	80561	1000	5000	0.001
p2	p3	p5	0.04966	0.001687	29.44	81045	1000	5000	0.001
p1	p4	p2	0.000989	0.000991	0.998	80748	1000	5000	0.001
p1	p4	p3	0.005342	0.001146	4.661	81650	1000	5000	0.001
p1	p5	p2	0.003248	0.001044	3.113	80667	1000	5000	0.001
p1	p5	p3	0.010428	0.001222	8.533	82078	1000	5000	0.001
p1	p5	p4	0.005654	0.001185	4.774	81007	1000	5000	0.001
p2	p3	p1	0.02099	0.001158	18.123	77468	1000	5000	0.001
p2	p3	p4	0.027788	0.001282	21.677	80561	1000	5000	0.001
p2	p3	p5	0.04966	0.001687	29.44	81045	1000	5000	0.001
p2	p4	p1	0.02014	0.001254	16.058	80748	1000	5000	0.001
p2	p4	p3	0.004514	0.001023	4.412	80561	1000	5000	0.001
p2	p4	p5	0.021669	0.001281	16.921	80510	1000	5000	0.001
p2	p5	p1	0.017844	0.001259	14.179	80667	1000	5000	0.001
p2	p5	p3	0.00736	0.001075	6.844	81045	1000	5000	0.001
p2	p5	p4	0.003411	0.001097	3.109	80510	1000	5000	0.001
p3	p4	p1	0.044334	0.001689	26.243	81650	1000	5000	0.001
p3	p4	p2	0.023951	0.00122	19.628	80561	1000	5000	0.001
p3	p4	p5	0.024602	0.001296	18.988	78060	1000	5000	0.001
p3	p5	p1	0.039118	0.001597	24.502	82078	1000	5000	0.001
p3	p5	p2	0.02108	0.001296	16.259	81045	1000	5000	0.001
p3	p5	p4	0.000558	0.00098	0.57	78060	1000	5000	0.001
p4	p5	p1	0.066137	0.002051	32.242	81007	1000	5000	0.001
p4	p5	p2	0.048491	0.001688	28.729	80510	1000	5000	0.001
p4	p5	p3	0.031689	0.001399	22.654	78060	1000	5000	0.001
p1	p2	p3	0.034421	0.001731	19.89	77951	100	5000	0.001

p1	p2	p4	0.043816	0.001829	23.95	79654	100	5000	0.001
p1	p2	p5	0.053531	0.002198	24.356	78741	100	5000	0.001
p2	p3	p1	0.054876	0.002253	24.352	77951	100	5000	0.001
p2	p3	p4	0.039377	0.001738	22.657	78867	100	5000	0.001
p2	p3	p5	0.057019	0.002145	26.579	79461	100	5000	0.001
p1	p4	p2	0.039407	0.001953	20.178	79654	100	5000	0.001
p1	p4	p3	0.029956	0.001716	17.459	79968	100	5000	0.001
p1	p5	p2	0.039716	0.001944	20.43	78741	100	5000	0.001
p1	p5	p3	0.037856	0.00188	20.139	80546	100	5000	0.001
p1	p5	p4	0.03095	0.001812	17.082	78985	100	5000	0.001
p2	p3	p1	0.054876	0.002253	24.352	77951	100	5000	0.001
p2	p3	p4	0.039377	0.001738	22.657	78867	100	5000	0.001
p2	p3	p5	0.057019	0.002145	26.579	79461	100	5000	0.001
p2	p4	p1	0.048554	0.002219	21.878	79654	100	5000	0.001
p2	p4	p3	0.023777	0.001538	15.462	78867	100	5000	0.001
p2	p4	p5	0.040399	0.0018	22.448	79203	100	5000	0.001
p2	p5	p1	0.04824	0.002322	20.779	78741	100	5000	0.001
p2	p5	p3	0.03137	0.001728	18.156	79461	100	5000	0.001
p2	p5	p4	0.030646	0.001719	17.828	79203	100	5000	0.001
p3	p4	p1	0.059444	0.002285	26.012	79968	100	5000	0.001
p3	p4	p2	0.043907	0.001847	23.777	78867	100	5000	0.001
p3	p4	p5	0.048106	0.001833	26.251	78837	100	5000	0.001
p3	p5	p1	0.051361	0.002305	22.287	80546	100	5000	0.001
p3	p5	p2	0.036252	0.001872	19.371	79461	100	5000	0.001
p3	p5	p4	0.023096	0.00163	14.167	78837	100	5000	0.001
p4	p5	p1	0.061791	0.002399	25.756	78985	100	5000	0.001
p4	p5	p2	0.052757	0.002106	25.049	79203	100	5000	0.001
p4	p5	p3	0.040151	0.001802	22.284	78837	100	5000	0.001
p1	p2	p3	0.02104	0.001183	17.787	78204	2000	5000	0.001
p1	p2	p4	0.042416	0.001345	31.526	80920	2000	5000	0.001
p1	p2	p5	0.06171	0.001903	32.423	81452	2000	5000	0.001
p2	p3	p1	0.026626	0.001275	20.886	78204	2000	5000	0.001
p2	p3	p4	0.022765	0.00103	22.109	80950	2000	5000	0.001
p2	p3	p5	0.042041	0.001568	26.814	82107	2000	5000	0.001
p1	p4	p2	-0.00292	0.000953	-3.067	80920	2000	5000	0.001
p1	p4	p3	0.001433	0.001018	1.407	81309	2000	5000	0.001
p1	p5	p2	-0.00198	0.000982	-2.014	81452	2000	5000	0.001
p1	p5	p3	0.00167	0.001096	1.524	82229	2000	5000	0.001
p1	p5	p4	0.00159	0.001023	1.555	80618	2000	5000	0.001
p2	p3	p1	0.026626	0.001275	20.886	78204	2000	5000	0.001
p2	p3	p4	0.022765	0.00103	22.109	80950	2000	5000	0.001
p2	p3	p5	0.042041	0.001568	26.814	82107	2000	5000	0.001
p2	p4	p1	0.027635	0.001372	20.142	80920	2000	5000	0.001
p2	p4	p3	0.00241	0.000881	2.735	80950	2000	5000	0.001
p2	p4	p5	0.020347	0.001213	16.78	81261	2000	5000	0.001
p2	p5	p1	0.026656	0.001454	18.329	81452	2000	5000	0.001
p2	p5	p3	0.001699	0.000963	1.764	82107	2000	5000	0.001
p2	p5	p4	0.00064	0.000932	0.687	81261	2000	5000	0.001
p3	p4	p1	0.046882	0.001656	28.309	81309	2000	5000	0.001
p3	p4	p2	0.016656	0.001055	15.784	80950	2000	5000	0.001
p3	p4	p5	0.019625	0.001185	16.563	78465	2000	5000	0.001
p3	p5	p1	0.046637	0.001751	26.63	82229	2000	5000	0.001
p3	p5	p2	0.017365	0.001136	15.288	82107	2000	5000	0.001
p3	p5	p4	0.001353	0.000925	1.462	78465	2000	5000	0.001
p4	p5	p1	0.06972	0.001944	35.872	80618	2000	5000	0.001
p4	p5	p2	0.0387	0.001362	28.419	81261	2000	5000	0.001
p4	p5	p3	0.023775	0.001126	21.118	78465	2000	5000	0.001
p1	p2	p3	0.049736	0.00197	25.251	77979	20	5000	0.001
p1	p2	p4	0.050978	0.001947	26.186	78485	20	5000	0.001
p1	p2	p5	0.052709	0.002069	25.476	78251	20	5000	0.001
p2	p3	p1	0.051424	0.00209	24.608	77979	20	5000	0.001
p2	p3	p4	0.051499	0.002049	25.131	78059	20	5000	0.001
p2	p3	p5	0.055712	0.002137	26.065	78080	20	5000	0.001
p1	p4	p2	0.049972	0.002082	23.999	78485	20	5000	0.001
p1	p4	p3	0.050254	0.002127	23.622	78162	20	5000	0.001
p1	p5	p2	0.047317	0.002016	23.474	78251	20	5000	0.001
p1	p5	p3	0.052704	0.002131	24.737	78450	20	5000	0.001
p1	p5	p4	0.050701	0.002053	24.694	78162	20	5000	0.001
p2	p3	p1	0.051424	0.00209	24.608	77979	20	5000	0.001
p2	p3	p4	0.051499	0.002049	25.131	78059	20	5000	0.001
p2	p3	p5	0.055712	0.002137	26.065	78080	20	5000	0.001

p2	p4	p1	0.047899	0.001978	24.214	78485	20	5000	0.001
p2	p4	p3	0.046717	0.001977	23.631	78059	20	5000	0.001
p2	p4	p5	0.052431	0.002061	25.445	78288	20	5000	0.001
p2	p5	p1	0.05057	0.002196	23.023	78251	20	5000	0.001
p2	p5	p3	0.051847	0.002121	24.444	78080	20	5000	0.001
p2	p5	p4	0.053394	0.00202	26.428	78288	20	5000	0.001
p3	p4	p1	0.050909	0.001897	26.831	78162	20	5000	0.001
p3	p4	p2	0.049459	0.002036	24.296	78059	20	5000	0.001
p3	p4	p5	0.057621	0.002192	26.292	77860	20	5000	0.001
p3	p5	p1	0.048466	0.002047	23.672	78450	20	5000	0.001
p3	p5	p2	0.044378	0.001963	22.612	78080	20	5000	0.001
p3	p5	p4	0.04824	0.002055	23.475	77860	20	5000	0.001
p4	p5	p1	0.048174	0.00197	24.452	78162	20	5000	0.001
p4	p5	p2	0.04759	0.002063	23.064	78288	20	5000	0.001
p4	p5	p3	0.049959	0.002239	22.315	77860	20	5000	0.001
p1	p2	p3	0.030056	0.001295	23.213	78355	4000	5000	0.001
p1	p2	p4	0.046173	0.001503	30.729	81882	4000	5000	0.001
p1	p2	p5	0.067703	0.002002	33.81	81614	4000	5000	0.001
p2	p3	p1	0.025802	0.001217	21.199	78355	4000	5000	0.001
p2	p3	p4	0.020015	0.001123	17.83	81493	4000	5000	0.001
p2	p3	p5	0.04211	0.001569	26.837	81954	4000	5000	0.001
p1	p4	p2	-0.00215	0.000904	-2.374	81882	4000	5000	0.001
p1	p4	p3	0.003712	0.000983	3.777	82024	4000	5000	0.001
p1	p5	p2	-0.00203	0.000947	-2.139	81614	4000	5000	0.001
p1	p5	p3	0.00488	0.001049	4.654	82529	4000	5000	0.001
p1	p5	p4	0.000767	0.001056	0.727	82382	4000	5000	0.001
p2	p3	p1	0.025802	0.001217	21.199	78355	4000	5000	0.001
p2	p3	p4	0.020015	0.001123	17.83	81493	4000	5000	0.001
p2	p3	p5	0.04211	0.001569	26.837	81954	4000	5000	0.001
p2	p4	p1	0.026388	0.001284	20.548	81882	4000	5000	0.001
p2	p4	p3	0.004284	0.000916	4.675	81493	4000	5000	0.001
p2	p4	p5	0.021218	0.00132	16.072	82239	4000	5000	0.001
p2	p5	p1	0.026264	0.00132	19.894	81614	4000	5000	0.001
p2	p5	p3	0.005331	0.000989	5.389	81954	4000	5000	0.001
p2	p5	p4	0.000648	0.000925	0.7	82239	4000	5000	0.001
p3	p4	p1	0.052836	0.001657	31.884	82024	4000	5000	0.001
p3	p4	p2	0.024056	0.001111	21.654	81493	4000	5000	0.001
p3	p4	p5	0.022283	0.001254	17.763	78523	4000	5000	0.001
p3	p5	p1	0.051638	0.00178	29.007	82529	4000	5000	0.001
p3	p5	p2	0.023015	0.001207	19.071	81954	4000	5000	0.001
p3	p5	p4	-0.00039	0.000899	-0.436	78523	4000	5000	0.001
p4	p5	p1	0.073315	0.002001	36.64	82382	4000	5000	0.001
p4	p5	p2	0.043455	0.001403	30.967	82239	4000	5000	0.001
p4	p5	p3	0.024835	0.001101	22.556	78523	4000	5000	0.001
p1	p2	p3	0.037705	0.00169	22.313	78177	50	5000	0.001
p1	p2	p4	0.048419	0.001862	26.011	78347	50	5000	0.001
p1	p2	p5	0.053415	0.002098	25.458	78420	50	5000	0.001
p2	p3	p1	0.054316	0.00209	25.986	78177	50	5000	0.001
p2	p3	p4	0.045742	0.001861	24.582	78081	50	5000	0.001
p2	p3	p5	0.053955	0.002108	25.597	79281	50	5000	0.001
p1	p4	p2	0.044137	0.001873	23.562	78347	50	5000	0.001
p1	p4	p3	0.035061	0.001727	20.303	79376	50	5000	0.001
p1	p5	p2	0.042539	0.001814	23.45	78420	50	5000	0.001
p1	p5	p3	0.038238	0.001803	21.212	80492	50	5000	0.001
p1	p5	p4	0.038831	0.001738	22.348	78769	50	5000	0.001
p2	p3	p1	0.054316	0.00209	25.986	78177	50	5000	0.001
p2	p3	p4	0.045742	0.001861	24.582	78081	50	5000	0.001
p2	p3	p5	0.053955	0.002108	25.597	79281	50	5000	0.001
p2	p4	p1	0.044596	0.001929	23.123	78347	50	5000	0.001
p2	p4	p3	0.025573	0.001597	16.012	78081	50	5000	0.001
p2	p4	p5	0.043818	0.001959	22.363	78598	50	5000	0.001
p2	p5	p1	0.0462	0.001878	24.595	78420	50	5000	0.001
p2	p5	p3	0.030316	0.001633	18.56	79281	50	5000	0.001
p2	p5	p4	0.040414	0.00177	22.827	78598	50	5000	0.001
p3	p4	p1	0.057025	0.002109	27.035	79376	50	5000	0.001
p3	p4	p2	0.046839	0.001729	27.093	78081	50	5000	0.001
p3	p4	p5	0.048622	0.001954	24.887	78676	50	5000	0.001
p3	p5	p1	0.05377	0.002011	26.735	80492	50	5000	0.001
p3	p5	p2	0.041995	0.001681	24.983	79281	50	5000	0.001
p3	p5	p4	0.035614	0.001759	20.253	78676	50	5000	0.001
p4	p5	p1	0.054303	0.002113	25.701	78769	50	5000	0.001

p4	p5	p2	0.052215	0.00203	25.717	78598	50	5000	0.001
p4	p5	p3	0.035581	0.001747	20.366	78676	50	5000	0.001
p1	p2	p3	0.04238	0.001879	22.553	78937	0	5000	0.01
p1	p2	p4	0.050589	0.002044	24.75	78318	0	5000	0.01
p1	p2	p5	0.052067	0.002113	24.647	77730	0	5000	0.01
p2	p3	p1	0.048243	0.002007	24.041	78937	0	5000	0.01
p2	p3	p4	0.046216	0.001927	23.98	78522	0	5000	0.01
p2	p3	p5	0.050637	0.002107	24.031	78617	0	5000	0.01
p1	p4	p2	0.0506	0.002175	23.264	78318	0	5000	0.01
p1	p4	p3	0.038028	0.001835	20.729	78155	0	5000	0.01
p1	p5	p2	0.049144	0.002167	22.674	77730	0	5000	0.01
p1	p5	p3	0.040966	0.001762	23.244	78173	0	5000	0.01
p1	p5	p4	0.051988	0.002213	23.489	77701	0	5000	0.01
p2	p3	p1	0.048243	0.002007	24.041	78937	0	5000	0.01
p2	p3	p4	0.046216	0.001927	23.98	78522	0	5000	0.01
p2	p3	p5	0.050637	0.002107	24.031	78617	0	5000	0.01
p2	p4	p1	0.050148	0.00215	23.321	78318	0	5000	0.01
p2	p4	p3	0.039912	0.001852	21.554	78522	0	5000	0.01
p2	p4	p5	0.053476	0.002062	25.931	77915	0	5000	0.01
p2	p5	p1	0.051609	0.002017	25.583	77730	0	5000	0.01
p2	p5	p3	0.044295	0.001922	23.042	78617	0	5000	0.01
p2	p5	p4	0.05344	0.002216	24.111	77915	0	5000	0.01
p3	p4	p1	0.052645	0.002069	25.443	78155	0	5000	0.01
p3	p4	p2	0.054991	0.002052	26.8	78522	0	5000	0.01
p3	p4	p5	0.057911	0.00223	25.965	77790	0	5000	0.01
p3	p5	p1	0.049672	0.002141	23.198	78173	0	5000	0.01
p3	p5	p2	0.050569	0.002096	24.129	78617	0	5000	0.01
p3	p5	p4	0.049035	0.002155	22.755	77790	0	5000	0.01
p4	p5	p1	0.048739	0.002018	24.156	77701	0	5000	0.01
p4	p5	p2	0.047739	0.002162	22.078	77915	0	5000	0.01
p4	p5	p3	0.037106	0.001806	20.548	77790	0	5000	0.01
p1	p2	p3	0.001033	0.000664	1.556	82590	1000	5000	0.01
p1	p2	p4	0.004448	0.000732	6.076	83193	1000	5000	0.01
p1	p2	p5	0.005574	0.000692	8.057	83444	1000	5000	0.01
p2	p3	p1	0.003171	0.000671	4.725	82590	1000	5000	0.01
p2	p3	p4	0.002806	0.00073	3.845	83354	1000	5000	0.01
p2	p3	p5	0.005468	0.000687	7.958	83626	1000	5000	0.01
p1	p4	p2	-0.00132	0.000668	-1.981	83193	1000	5000	0.01
p1	p4	p3	-0.00062	0.000659	-0.935	83551	1000	5000	0.01
p1	p5	p2	-0.00028	0.000668	-0.418	83444	1000	5000	0.01
p1	p5	p3	0.000926	0.000715	1.297	83655	1000	5000	0.01
p1	p5	p4	-0.0003	0.000661	-0.46	83074	1000	5000	0.01
p2	p3	p1	0.003171	0.000671	4.725	82590	1000	5000	0.01
p2	p3	p4	0.002806	0.00073	3.845	83354	1000	5000	0.01
p2	p3	p5	0.005468	0.000687	7.958	83626	1000	5000	0.01
p2	p4	p1	0.003035	0.000662	4.585	83193	1000	5000	0.01
p2	p4	p3	-0.00075	0.000641	-1.174	83354	1000	5000	0.01
p2	p4	p5	0.000844	0.000648	1.301	83007	1000	5000	0.01
p2	p5	p1	0.001989	0.000635	3.133	83444	1000	5000	0.01
p2	p5	p3	-0.00026	0.000659	-0.393	83626	1000	5000	0.01
p2	p5	p4	-0.00135	0.000639	-2.109	83007	1000	5000	0.01
p3	p4	p1	0.004815	0.000722	6.668	83551	1000	5000	0.01
p3	p4	p2	0.000319	0.000655	0.487	83354	1000	5000	0.01
p3	p4	p5	0.001333	0.000727	1.834	82729	1000	5000	0.01
p3	p5	p1	0.003277	0.000664	4.933	83655	1000	5000	0.01
p3	p5	p2	-0.00017	0.000644	-0.268	83626	1000	5000	0.01
p3	p5	p4	-0.00184	0.000605	-3.039	82729	1000	5000	0.01
p4	p5	p1	0.007793	0.000712	10.944	83074	1000	5000	0.01
p4	p5	p2	0.004476	0.000736	6.078	83007	1000	5000	0.01
p4	p5	p3	0.003916	0.000706	5.546	82729	1000	5000	0.01
p1	p2	p3	0.008827	0.000835	10.573	82455	100	5000	0.01
p1	p2	p4	0.017872	0.001017	17.575	83115	100	5000	0.01
p1	p2	p5	0.031274	0.00126	24.831	83487	100	5000	0.01
p2	p3	p1	0.00825	0.000807	10.219	82455	100	5000	0.01
p2	p3	p4	0.005881	0.000812	7.241	83942	100	5000	0.01
p2	p3	p5	0.017915	0.001052	17.03	84051	100	5000	0.01
p1	p4	p2	-0.00468	0.000749	-6.252	83115	100	5000	0.01
p1	p4	p3	-0.00305	0.000731	-4.18	83946	100	5000	0.01
p1	p5	p2	-0.00366	0.00082	-4.467	83487	100	5000	0.01
p1	p5	p3	-0.00415	0.000742	-5.601	84022	100	5000	0.01
p1	p5	p4	-0.00299	0.000906	-3.297	83305	100	5000	0.01

p2	p3	p1	0.00825	0.000807	10.219	82455	100	5000	0.01
p2	p3	p4	0.005881	0.000812	7.241	83942	100	5000	0.01
p2	p3	p5	0.017915	0.001052	17.03	84051	100	5000	0.01
p2	p4	p1	0.009285	0.000846	10.979	83115	100	5000	0.01
p2	p4	p3	-0.00204	0.000685	-2.982	83942	100	5000	0.01
p2	p4	p5	0.010006	0.000886	11.292	83296	100	5000	0.01
p2	p5	p1	0.008251	0.00085	9.703	83487	100	5000	0.01
p2	p5	p3	-0.00415	0.000704	-5.896	84051	100	5000	0.01
p2	p5	p4	-0.00401	0.000772	-5.188	83296	100	5000	0.01
p3	p4	p1	0.020422	0.001014	20.134	83946	100	5000	0.01
p3	p4	p2	0.00731	0.000885	8.256	83942	100	5000	0.01
p3	p4	p5	0.007834	0.00086	9.105	81718	100	5000	0.01
p3	p5	p1	0.02155	0.00104	20.725	84022	100	5000	0.01
p3	p5	p2	0.00944	0.000946	9.98	84051	100	5000	0.01
p3	p5	p4	-0.00188	0.000774	-2.425	81718	100	5000	0.01
p4	p5	p1	0.030459	0.001188	25.633	83305	100	5000	0.01
p4	p5	p2	0.017199	0.000995	17.293	83296	100	5000	0.01
p4	p5	p3	0.005643	0.000807	6.99	81718	100	5000	0.01
p1	p2	p3	0.002292	0.000664	3.45	83180	2000	5000	0.01
p1	p2	p4	0.005493	0.000683	8.037	83337	2000	5000	0.01
p1	p2	p5	0.013361	0.000852	15.684	83121	2000	5000	0.01
p2	p3	p1	0.001744	0.000693	2.517	83180	2000	5000	0.01
p2	p3	p4	0.002844	0.000657	4.329	84271	2000	5000	0.01
p2	p3	p5	0.010668	0.000856	12.463	84068	2000	5000	0.01
p1	p4	p2	-0.00172	0.000652	-2.643	83337	2000	5000	0.01
p1	p4	p3	-0.00036	0.000647	-0.555	84244	2000	5000	0.01
p1	p5	p2	-0.00214	0.000646	-3.317	83121	2000	5000	0.01
p1	p5	p3	-0.00036	0.000719	-0.496	83837	2000	5000	0.01
p1	p5	p4	-0.0021	0.000674	-3.106	82885	2000	5000	0.01
p2	p3	p1	0.001744	0.000693	2.517	83180	2000	5000	0.01
p2	p3	p4	0.002844	0.000657	4.329	84271	2000	5000	0.01
p2	p3	p5	0.010668	0.000856	12.463	84068	2000	5000	0.01
p2	p4	p1	0.000987	0.000674	1.465	83337	2000	5000	0.01
p2	p4	p3	-0.00111	0.000609	-1.823	84271	2000	5000	0.01
p2	p4	p5	0.00564	0.000776	7.264	82850	2000	5000	0.01
p2	p5	p1	0.001406	0.000695	2.024	83121	2000	5000	0.01
p2	p5	p3	-0.00069	0.000641	-1.082	84068	2000	5000	0.01
p2	p5	p4	-0.00168	0.000667	-2.519	82850	2000	5000	0.01
p3	p4	p1	0.004414	0.000736	5.995	84244	2000	5000	0.01
p3	p4	p2	0.00093	0.000655	1.421	84271	2000	5000	0.01
p3	p4	p5	0.006065	0.000766	7.924	82554	2000	5000	0.01
p3	p5	p1	0.004411	0.000757	5.827	83837	2000	5000	0.01
p3	p5	p2	0.000512	0.000699	0.732	84068	2000	5000	0.01
p3	p5	p4	-0.0021	0.000683	-3.071	82554	2000	5000	0.01
p4	p5	p1	0.008634	0.000767	11.254	82885	2000	5000	0.01
p4	p5	p2	0.005465	0.000742	7.361	82850	2000	5000	0.01
p4	p5	p3	0.003835	0.000703	5.456	82554	2000	5000	0.01
p1	p2	p3	0.023656	0.001402	16.868	79588	20	5000	0.01
p1	p2	p4	0.022467	0.001317	17.063	82691	20	5000	0.01
p1	p2	p5	0.051795	0.001879	27.567	80828	20	5000	0.01
p2	p3	p1	0.040958	0.001755	23.334	79588	20	5000	0.01
p2	p3	p4	0.014993	0.00113	13.27	80978	20	5000	0.01
p2	p3	p5	0.054222	0.001783	30.417	81076	20	5000	0.01
p1	p4	p2	0.017934	0.001284	13.965	82691	20	5000	0.01
p1	p4	p3	0.015974	0.001324	12.062	82245	20	5000	0.01
p1	p5	p2	0.021727	0.001457	14.913	80828	20	5000	0.01
p1	p5	p3	0.026039	0.001633	15.944	82196	20	5000	0.01
p1	p5	p4	0.007717	0.001151	6.706	81587	20	5000	0.01
p2	p3	p1	0.040958	0.001755	23.334	79588	20	5000	0.01
p2	p3	p4	0.014993	0.00113	13.27	80978	20	5000	0.01
p2	p3	p5	0.054222	0.001783	30.417	81076	20	5000	0.01
p2	p4	p1	0.033393	0.001568	21.294	82691	20	5000	0.01
p2	p4	p3	0.008561	0.001123	7.626	80978	20	5000	0.01
p2	p4	p5	0.036358	0.001574	23.098	81782	20	5000	0.01
p2	p5	p1	0.029544	0.001587	18.619	80828	20	5000	0.01
p2	p5	p3	0.014852	0.001367	10.864	81076	20	5000	0.01
p2	p5	p4	0.004046	0.001087	3.723	81782	20	5000	0.01
p3	p4	p1	0.048795	0.001794	27.2	82245	20	5000	0.01
p3	p4	p2	0.025656	0.001378	18.616	80978	20	5000	0.01
p3	p4	p5	0.042765	0.00161	26.566	79529	20	5000	0.01
p3	p5	p1	0.038526	0.001754	21.967	82196	20	5000	0.01

p3	p5	p2	0.019331	0.001487	12.999	81076	20	5000	0.01
p3	p5	p4	-0.00208	0.001019	-2.038	79529	20	5000	0.01
p4	p5	p1	0.04886	0.001714	28.499	81587	20	5000	0.01
p4	p5	p2	0.036964	0.001524	24.255	81782	20	5000	0.01
p4	p5	p3	0.026102	0.001359	19.203	79529	20	5000	0.01
p1	p2	p3	0.002608	0.000684	3.814	83379	4000	5000	0.01
p1	p2	p4	0.005386	0.000709	7.596	83854	4000	5000	0.01
p1	p2	p5	0.010582	0.00076	13.927	84390	4000	5000	0.01
p2	p3	p1	0.000799	0.000595	1.342	83379	4000	5000	0.01
p2	p3	p4	0.002168	0.000654	3.313	83574	4000	5000	0.01
p2	p3	p5	0.005901	0.000694	8.508	83775	4000	5000	0.01
p1	p4	p2	-0.00014	0.000569	-0.249	83854	4000	5000	0.01
p1	p4	p3	-0.00062	0.000628	-0.982	83774	4000	5000	0.01
p1	p5	p2	-0.00032	0.000624	-0.506	84390	4000	5000	0.01
p1	p5	p3	-0.00208	0.00061	-3.413	84190	4000	5000	0.01
p1	p5	p4	-0.00104	0.000637	-1.629	83558	4000	5000	0.01
p2	p3	p1	0.000799	0.000595	1.342	83379	4000	5000	0.01
p2	p3	p4	0.002168	0.000654	3.313	83574	4000	5000	0.01
p2	p3	p5	0.005901	0.000694	8.508	83775	4000	5000	0.01
p2	p4	p1	0.001363	0.000662	2.059	83854	4000	5000	0.01
p2	p4	p3	-5.1E-05	0.00064	-0.079	83574	4000	5000	0.01
p2	p4	p5	0.004157	0.000686	6.061	83443	4000	5000	0.01
p2	p5	p1	0.001537	0.000699	2.199	84390	4000	5000	0.01
p2	p5	p3	-0.00134	0.00059	-2.271	83775	4000	5000	0.01
p2	p5	p4	-0.00086	0.000594	-1.452	83443	4000	5000	0.01
p3	p4	p1	0.00401	0.000685	5.857	83774	4000	5000	0.01
p3	p4	p2	0.003057	0.000636	4.81	83574	4000	5000	0.01
p3	p4	p5	0.00287	0.000643	4.466	82487	4000	5000	0.01
p3	p5	p1	0.005468	0.00073	7.485	84190	4000	5000	0.01
p3	p5	p2	0.004335	0.000707	6.136	83775	4000	5000	0.01
p3	p5	p4	0.000424	0.000636	0.667	82487	4000	5000	0.01
p4	p5	p1	0.007771	0.000767	10.128	83558	4000	5000	0.01
p4	p5	p2	0.006068	0.000675	8.986	83443	4000	5000	0.01
p4	p5	p3	0.001697	0.000639	2.657	82487	4000	5000	0.01
p1	p2	p3	0.013778	0.000955	14.422	81109	50	5000	0.01
p1	p2	p4	0.030635	0.00128	23.937	82560	50	5000	0.01
p1	p2	p5	0.044198	0.001512	29.231	82577	50	5000	0.01
p2	p3	p1	0.016432	0.000945	17.396	81109	50	5000	0.01
p2	p3	p4	0.011304	0.000935	12.084	83208	50	5000	0.01
p2	p3	p5	0.030696	0.001219	25.19	84322	50	5000	0.01
p1	p4	p2	-0.00632	0.000807	-7.825	82560	50	5000	0.01
p1	p4	p3	-0.00534	0.000787	-6.777	82996	50	5000	0.01
p1	p5	p2	-0.00451	0.000867	-5.198	82577	50	5000	0.01
p1	p5	p3	0.000688	0.000928	0.741	83672	50	5000	0.01
p1	p5	p4	0.005496	0.001102	4.989	82507	50	5000	0.01
p2	p3	p1	0.016432	0.000945	17.396	81109	50	5000	0.01
p2	p3	p4	0.011304	0.000935	12.084	83208	50	5000	0.01
p2	p3	p5	0.030696	0.001219	25.19	84322	50	5000	0.01
p2	p4	p1	0.017806	0.001047	17.006	82560	50	5000	0.01
p2	p4	p3	-0.00401	0.000697	-5.756	83208	50	5000	0.01
p2	p4	p5	0.01856	0.001067	17.395	83272	50	5000	0.01
p2	p5	p1	0.015954	0.001065	14.985	82577	50	5000	0.01
p2	p5	p3	0.000226	0.000808	0.28	84322	50	5000	0.01
p2	p5	p4	0.003687	0.000933	3.953	83272	50	5000	0.01
p3	p4	p1	0.036228	0.001358	26.674	82996	50	5000	0.01
p3	p4	p2	0.012996	0.000887	14.649	83208	50	5000	0.01
p3	p4	p5	0.022929	0.001114	20.582	80880	50	5000	0.01
p3	p5	p1	0.029988	0.001369	21.91	83672	50	5000	0.01
p3	p5	p2	0.008716	0.0009	9.681	84322	50	5000	0.01
p3	p5	p4	-0.0006	0.000831	-0.719	80880	50	5000	0.01
p4	p5	p1	0.043549	0.001497	29.095	82507	50	5000	0.01
p4	p5	p2	0.020604	0.001078	19.108	83272	50	5000	0.01
p4	p5	p3	0.007757	0.000869	8.928	80880	50	5000	0.01

Table S4. Data underlying figure 4f. Data underlying main figure 4f, showing f3-scores for population triplets, calculated with the software admixtools, for an in-silico meta-population, simulated with the software SLIM2, which experiences a pulse admixture event at t=0. See main text for more details.

source1	source2	target	f3	std_err	Z	n_snps	n_generations	Ne	admix_prop
p11	p12	p22	0.045134	0.001385	32.588	99899	0	5000	0.1
p11	p22	p12	-0.00462	0.000839	-5.511	99899	0	5000	0.1
p12	p22	p11	0.007532	0.000973	7.741	99899	0	5000	0.1
p11	p12	p22	0.018104	0.000923	19.616	99909	0	5000	0.5
p11	p22	p12	-0.01202	0.000685	-17.543	99909	0	5000	0.5
p12	p22	p11	0.027579	0.001194	23.099	99909	0	5000	0.5
p11	p12	p22	0.099499	0.002654	37.496	99874	1000	5000	0.1
p11	p22	p12	0.05059	0.002191	23.085	99874	1000	5000	0.1
p12	p22	p11	0.057703	0.002312	24.962	99874	1000	5000	0.1
p11	p12	p22	0.076317	0.002284	33.419	99898	1000	5000	0.5
p11	p22	p12	0.038285	0.002036	18.807	99898	1000	5000	0.5
p12	p22	p11	0.080227	0.002605	30.801	99898	1000	5000	0.5
p11	p12	p22	0.052752	0.001533	34.413	99912	100	5000	0.1
p11	p22	p12	0.005117	0.001042	4.913	99912	100	5000	0.1
p12	p22	p11	0.00964	0.001065	9.052	99912	100	5000	0.1
p11	p12	p22	0.03009	0.001125	26.747	99865	100	5000	0.5
p11	p22	p12	-0.00641	0.000806	-7.952	99865	100	5000	0.5
p12	p22	p11	0.027314	0.001162	23.504	99865	100	5000	0.5
p11	p12	p22	0.092385	0.002837	32.56	99907	1500	5000	0.5
p11	p22	p12	0.066945	0.002472	27.086	99907	1500	5000	0.5
p12	p22	p11	0.105233	0.002932	35.89	99907	1500	5000	0.5
p11	p12	p22	0.139611	0.003678	37.963	99894	2000	5000	0.1
p11	p22	p12	0.095668	0.003349	28.564	99894	2000	5000	0.1
p12	p22	p11	0.111281	0.003558	31.279	99894	2000	5000	0.1
p11	p12	p22	0.126908	0.003615	35.101	99906	2000	5000	0.5
p11	p22	p12	0.08539	0.003125	27.325	99906	2000	5000	0.5
p12	p22	p11	0.128442	0.003704	34.676	99906	2000	5000	0.5
p11	p12	p22	0.057613	0.001713	33.641	99882	200	5000	0.1
p11	p22	p12	0.003529	0.001012	3.486	99882	200	5000	0.1
p12	p22	p11	0.011828	0.001061	11.15	99882	200	5000	0.1
p11	p12	p22	0.03385	0.001285	26.334	99905	200	5000	0.5
p11	p22	p12	-0.00463	0.00085	-5.451	99905	200	5000	0.5
p12	p22	p11	0.035835	0.001392	25.745	99905	200	5000	0.5
p11	p12	p22	0.04951	0.001497	33.082	99891	20	5000	0.1
p11	p22	p12	-0.00475	0.000834	-5.688	99891	20	5000	0.1
p12	p22	p11	0.004968	0.000874	5.687	99891	20	5000	0.1
p11	p12	p22	0.023817	0.001068	22.299	99854	20	5000	0.5
p11	p22	p12	-0.01037	0.000714	-14.525	99854	20	5000	0.5
p12	p22	p11	0.024908	0.001102	22.602	99854	20	5000	0.5
p11	p12	p22	0.28244	0.006897	40.949	99891	4000	5000	0.1
p11	p22	p12	0.179646	0.005119	35.094	99891	4000	5000	0.1
p12	p22	p11	0.215134	0.005837	36.859	99891	4000	5000	0.1
p11	p12	p22	0.228427	0.005949	38.395	99900	4000	5000	0.5
p11	p22	p12	0.195271	0.005787	33.744	99900	4000	5000	0.5
p12	p22	p11	0.261976	0.006775	38.669	99900	4000	5000	0.5
p11	p12	p22	0.065659	0.001942	33.815	99892	400	5000	0.1
p11	p22	p12	0.014488	0.001298	11.159	99892	400	5000	0.1
p12	p22	p11	0.026666	0.00136	19.603	99892	400	5000	0.1
p11	p12	p22	0.044175	0.001623	27.217	99904	400	5000	0.5
p11	p22	p12	0.00746	0.001238	6.025	99904	400	5000	0.5
p12	p22	p11	0.046705	0.001566	29.822	99904	400	5000	0.5
p11	p12	p22	0.044242	0.001326	33.361	99930	50	5000	0.1
p11	p22	p12	0.000727	0.000948	0.767	99930	50	5000	0.1
p12	p22	p11	0.005957	0.00091	6.544	99930	50	5000	0.1
p11	p12	p22	0.029183	0.001136	25.695	99890	50	5000	0.5
p11	p22	p12	-0.00956	0.00073	-13.093	99890	50	5000	0.5
p12	p22	p11	0.026841	0.001166	23.014	99890	50	5000	0.5
p11	p12	p22	0.057012	0.001842	30.954	99903	600	5000	0.5
p12	p22	p11	0.055573	0.001766	31.475	99903	600	5000	0.5

p11	p12	p22	0.067947	0.002028	33.513	99899	800	5000	0.5
p11	p22	p12	0.030189	0.001802	16.755	99899	800	5000	0.5
p12	p22	p11	0.065893	0.002191	30.075	99899	800	5000	0.5
p11	p12	p22	0.035587	0.001191	29.876	99892	0	10000	0.1
p11	p22	p12	-0.00533	0.0008	-6.66	99892	0	10000	0.1
p12	p22	p11	0.008644	0.000911	9.489	99892	0	10000	0.1
p11	p12	p22	0.035057	0.001195	29.343	99907	0	10000	0.5
p11	p22	p12	-0.01109	0.000647	-17.134	99907	0	10000	0.5
p12	p22	p11	0.01378	0.000909	15.156	99907	0	10000	0.5
p11	p12	p22	0.072297	0.001967	36.761	99908	1000	10000	0.1
p11	p22	p12	0.016511	0.001309	12.614	99908	1000	10000	0.1
p12	p22	p11	0.029028	0.001419	20.452	99908	1000	10000	0.1
p11	p12	p22	0.050944	0.001817	28.03	99891	1000	10000	0.5
p11	p22	p12	0.012748	0.001269	10.046	99891	1000	10000	0.5
p12	p22	p11	0.050667	0.001711	29.606	99891	1000	10000	0.5
p11	p12	p22	0.048481	0.001316	36.836	99880	100	10000	0.1
p11	p22	p12	-0.00164	0.000802	-2.046	99880	100	10000	0.1
p12	p22	p11	0.004818	0.000914	5.273	99880	100	10000	0.1
p11	p12	p22	0.032551	0.001266	25.711	99890	100	10000	0.5
p11	p22	p12	-0.0076	0.000789	-9.629	99890	100	10000	0.5
p12	p22	p11	0.024314	0.001131	21.494	99890	100	10000	0.5
p11	p12	p22	0.059261	0.001934	30.642	99935	1500	10000	0.5
p11	p22	p12	0.026509	0.001628	16.284	99935	1500	10000	0.5
p12	p22	p11	0.062307	0.002046	30.46	99935	1500	10000	0.5
p11	p12	p22	0.092591	0.002483	37.288	99900	2000	10000	0.1
p11	p22	p12	0.04759	0.001901	25.037	99900	2000	10000	0.1
p12	p22	p11	0.053443	0.002177	24.545	99900	2000	10000	0.1
p11	p12	p22	0.07855	0.002371	33.129	99916	2000	10000	0.5
p11	p22	p12	0.03793	0.001774	21.386	99916	2000	10000	0.5
p12	p22	p11	0.074415	0.002507	29.68	99916	2000	10000	0.5
p11	p12	p22	0.049647	0.001465	33.882	99889	200	10000	0.1
p11	p22	p12	-0.00176	0.000889	-1.974	99889	200	10000	0.1
p12	p22	p11	0.011252	0.001005	11.194	99889	200	10000	0.1
p11	p12	p22	0.030919	0.001217	25.406	99896	200	10000	0.5
p11	p22	p12	-0.00833	0.000789	-10.563	99896	200	10000	0.5
p12	p22	p11	0.029959	0.001219	24.578	99896	200	10000	0.5
p11	p12	p22	0.043921	0.001403	31.296	99876	20	10000	0.1
p11	p22	p12	-0.00138	0.000786	-1.752	99876	20	10000	0.1
p12	p22	p11	0.003757	0.000871	4.315	99876	20	10000	0.1
p11	p12	p22	0.0265	0.00113	23.447	99876	20	10000	0.5
p11	p22	p12	-0.01021	0.000747	-13.676	99876	20	10000	0.5
p12	p22	p11	0.025515	0.001108	23.03	99876	20	10000	0.5
p11	p12	p22	0.031371	0.001243	25.246	99902	300	10000	0.5
p11	p22	p12	-0.00239	0.000959	-2.495	99902	300	10000	0.5
p12	p22	p11	0.035788	0.001405	25.466	99902	300	10000	0.5
p11	p12	p22	0.143337	0.00369	38.849	99915	4000	10000	0.1
p11	p22	p12	0.094093	0.002925	32.168	99915	4000	10000	0.1
p12	p22	p11	0.097219	0.002922	33.273	99915	4000	10000	0.1
p11	p12	p22	0.116101	0.003349	34.666	99928	4000	10000	0.5
p11	p22	p12	0.089398	0.002838	31.503	99928	4000	10000	0.5
p12	p22	p11	0.116472	0.00343	33.953	99928	4000	10000	0.5
p11	p12	p22	0.051512	0.001513	34.041	99917	400	10000	0.1
p11	p22	p12	0.002789	0.000943	2.956	99917	400	10000	0.1
p12	p22	p11	0.017653	0.001218	14.495	99917	400	10000	0.1
p11	p12	p22	0.03642	0.001433	25.419	99895	400	10000	0.5
p11	p22	p12	-0.00245	0.000907	-2.696	99895	400	10000	0.5
p12	p22	p11	0.032105	0.001222	26.28	99895	400	10000	0.5
p11	p12	p22	0.046766	0.00137	34.129	99902	50	10000	0.1
p11	p22	p12	-0.00022	0.000862	-0.255	99902	50	10000	0.1
p12	p22	p11	0.00421	0.000894	4.708	99902	50	10000	0.1
p11	p12	p22	0.02358	0.00113	20.872	99862	50	10000	0.5
p11	p22	p12	-0.01097	0.000727	-15.089	99862	50	10000	0.5
p12	p22	p11	0.025282	0.001039	24.324	99862	50	10000	0.5
p11	p12	p22	0.040283	0.001483	27.162	99905	600	10000	0.5
p11	p22	p12	0.002717	0.00101	2.69	99905	600	10000	0.5
p12	p22	p11	0.044755	0.001527	29.301	99905	600	10000	0.5
p11	p12	p22	0.044156	0.001647	26.808	99920	800	10000	0.5
p11	p22	p12	0.011301	0.001248	9.056	99920	800	10000	0.5
p12	p22	p11	0.047187	0.001688	27.952	99920	800	10000	0.5

Table S5. Data underlying figure 6f. Data underlying main figure 6f, showing ROH-statistics (froh and nroh), calculated with the software Darwindow, for in-silico populations, simulated with the software SLIM2, which a t=0 experience a population size decrease. See main text for more details.

ngen	froh	nroh	ne
0	0.034	9	50
20	0.179	10.8	50
50	0.446	17.4	50
100	0.597	23.4	50
200	0.84	18.2	50
500	0.991	4.4	50
1000	0.998	3.8	50
1500	0.994	4.8	50
2000	1	3.2	50
4000	0.995	4.8	50
8000	1	2	50
12000	1	2.6	50
0	0.031	8.8	100
20	0.077	10	100
50	0.223	13.2	100
100	0.39	19.6	100
200	0.582	35.8	100
500	0.916	20.2	100
1000	0.943	23.2	100
1500	0.956	21.4	100
2000	0.973	14	100
4000	0.969	17	100
8000	0.967	16.8	100
12000	0.967	16	100
0	0.029	8.4	200
20	0.157	10.2	200
50	0.155	12.6	200
100	0.155	12.6	200
200	0.279	32.2	200
500	0.605	56.2	200
1000	0.807	45.2	200
1500	0.867	49.2	200
2000	0.886	37.4	200
4000	0.878	45.2	200
8000	0.87	47	200
12000	0.898	40.4	200
0	0.055	10.2	500
20	0.044	9.2	500
50	0.042	9.4	500
100	0.115	14.4	500
200	0.165	23	500
500	0.273	47.2	500
1000	0.417	71.6	500
1500	0.491	74.4	500
2000	0.51	80.8	500
4000	0.595	86.4	500
8000	0.548	90	500
12000	0.512	94.4	500
0	0.022	7.8	1000
20	0.035	8.6	1000
50	0.043	11.6	1000
100	0.058	12.4	1000
200	0.103	16.4	1000
500	0.114	24.6	1000
1000	0.153	43.2	1000
1500	0.216	52.6	1000
2000	0.21	55.2	1000
4000	0.243	63.2	1000
8000	0.29	68.6	1000

12000	0.252	68	1000
0	0.03	8.2	5000
20	0.021	7.6	5000
50	0.02	7.8	5000
100	0.025	8.8	5000
200	0.044	5	5000
500	0.024	6.8	5000
1000	0.029	8.4	5000
1500	0.029	8.4	5000
2000	0.037	11	5000
4000	0.039	8.8	5000
8000	0.039	8.8	5000
12000	0.039	8.8	5000

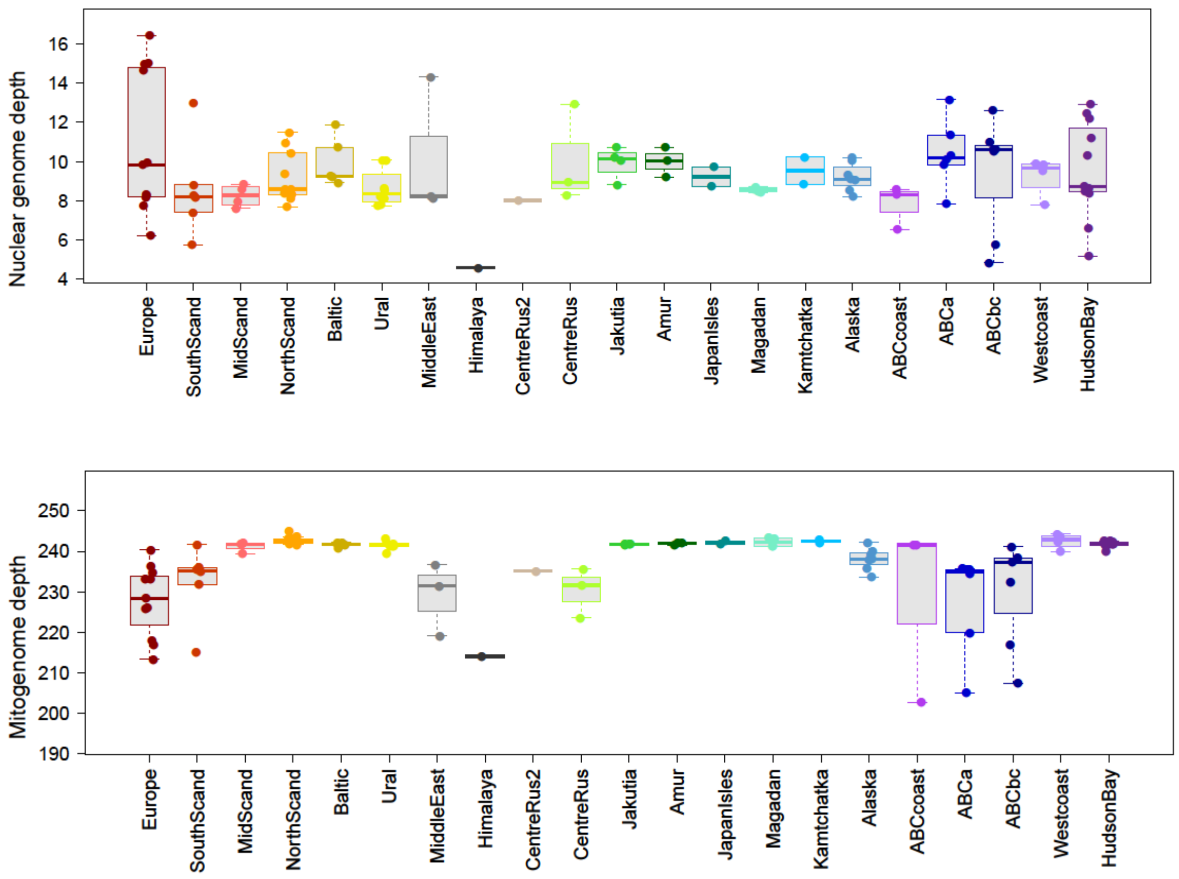

Fig. S1A. Mean sequencing depth per sample. Above: Mean depth per sample for the nuclear genome. **Below**: Mean depth per sample for the mitogenome. (Not shown are polar bear and black bear samples, and samples from the populations 'Aleutian' and 'Kodiak'. Samples from the populations 'Sakhalin' and 'Hokkaido' (1x) are grouped within the population 'JapanIsles', and the populations 'ABCcoast1' and 'ABCcoast' are grouped together.) Note that the maximum depth for genotype calling was restricted to 250 reads (default of bcftools mpileup), which explains the observed average mitogenome depth.

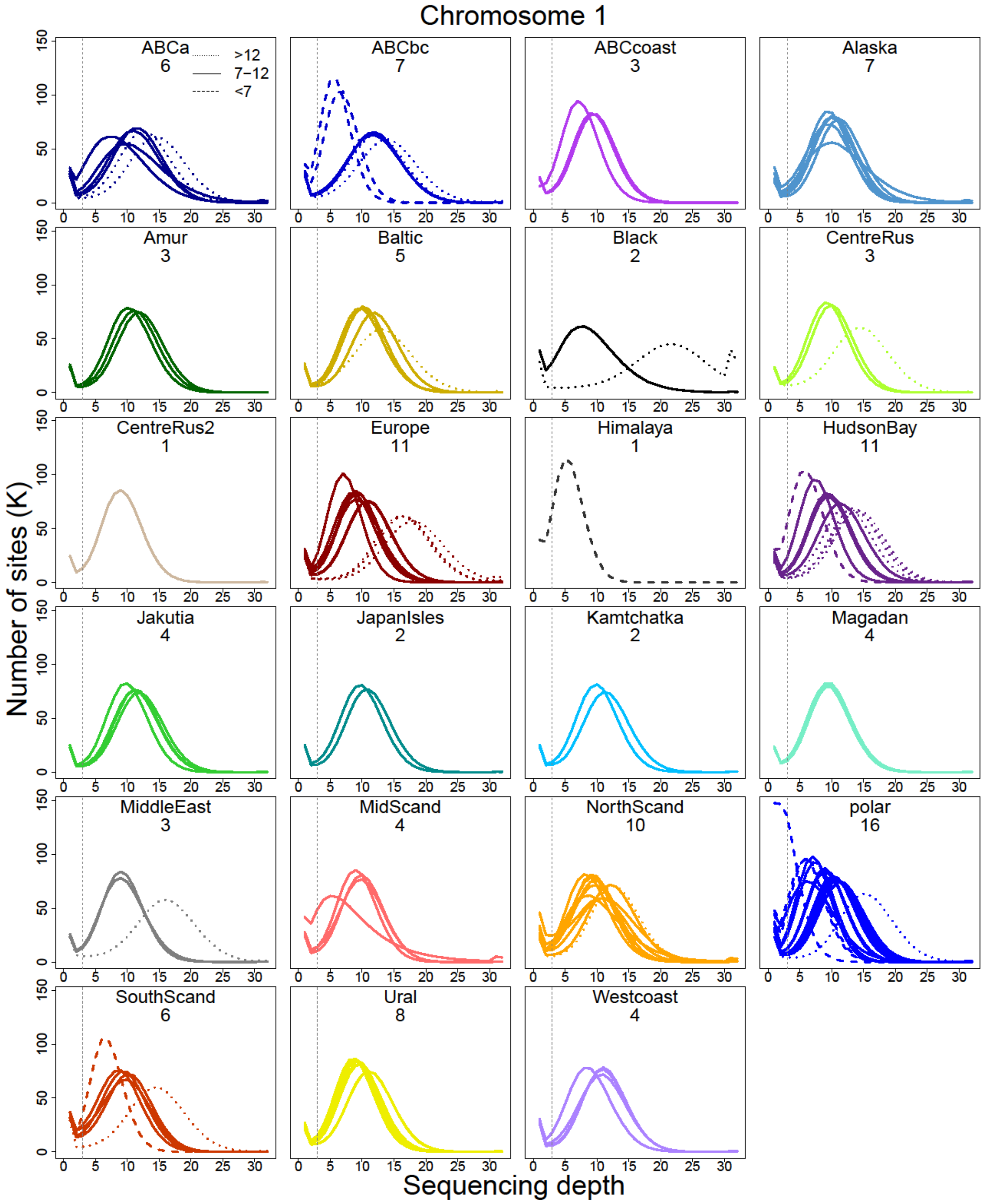

Fig. S1B. Distribution of read depth across sites along the nuclear genome. Shown is the distribution of read depth per site per individual for a randomly thinned dataset. Numbers represent the number of individuals per populations. Dashed, solid and dotted lines indicate genomes with a mean depth of respectively <7, 7-12, and >12. (Not shown are samples from the populations 'Aleutian' and 'Kodiak'. Samples from 'Sakhalin' and 'Hokkaido' (1x) are grouped within the population 'JapanIsles', and the populations 'ABCcoast1' and 'ABCcoast' are grouped together.)

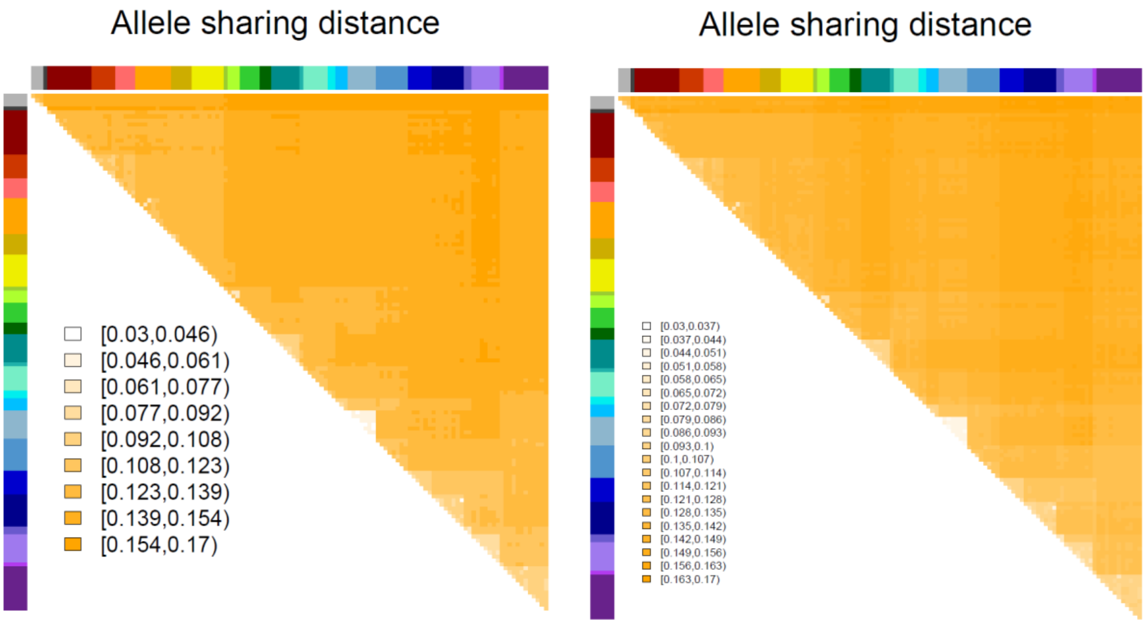

Fig. S1C. Distance matrix underlying dendrogram. Allele sharing distances between individuals, underlying bioNJ dendrogram for autosomal SNP dataset (presented in main Figure 1f), with distances divided in either 9 or 20 bins. Not shown are polar bear and black bear individuals.

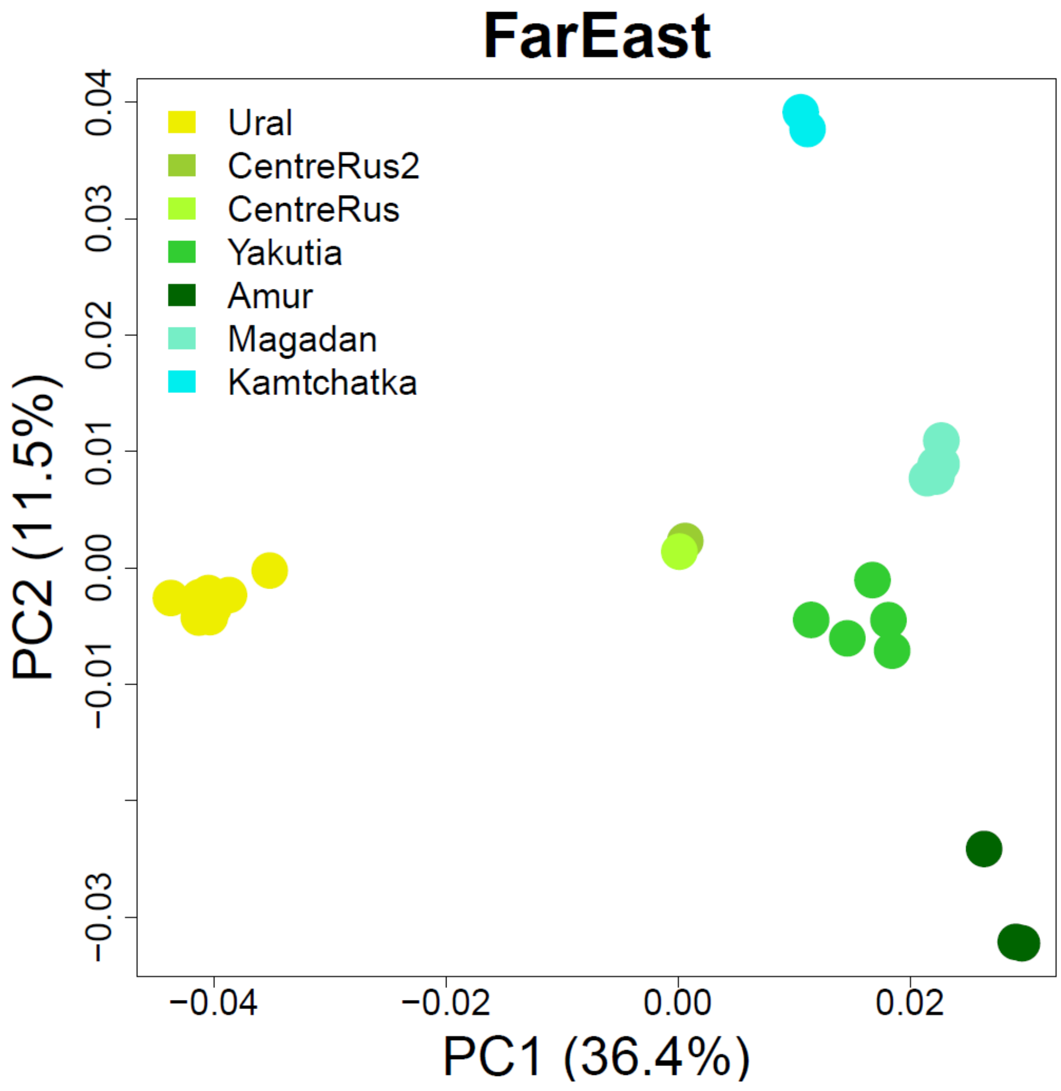

Fig. S1D. PCoA-plot showing genetic structure in central and eastern Eurasia. Biplot showing the first two of principal coordinate analyses (PCoA) based on allele sharing distances for the subset of central and eastern Eurasian samples, except for Hokkaido. From the three related 'CentreRus' individuals, only one is included in the analysis.

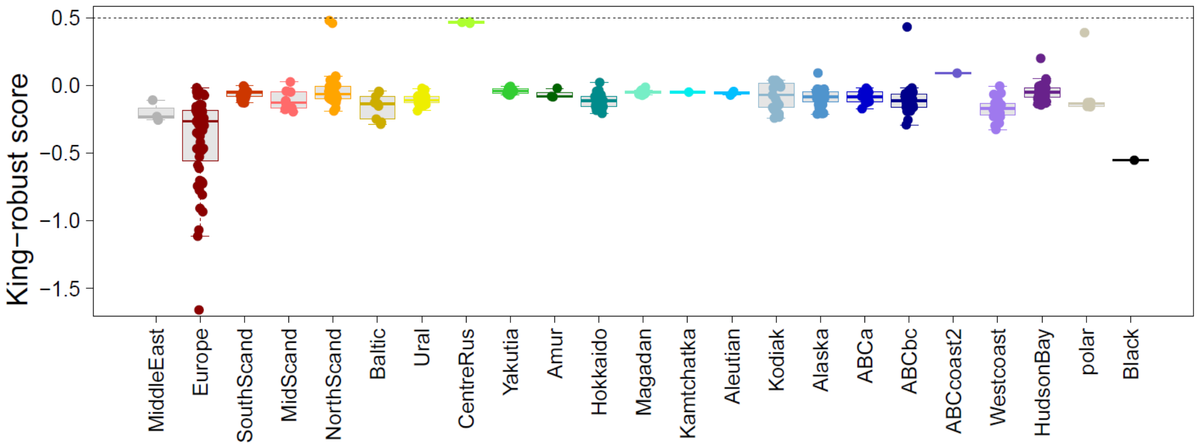

Fig. S1E. Relatedness between individuals. Boxplot showing relatedness between individuals, measured with the king-robust score, for all possible sample comparisons within populations. A score close to 0.5 is indicative of kinship. The populations 'NorthScand', 'ABCbc' and 'polar' contain a few related individuals, of which one resulting from duplex sequencing, All three 'CentreRus' individuals (one sequenced for another study and obtained from NCBI SRA) are related.

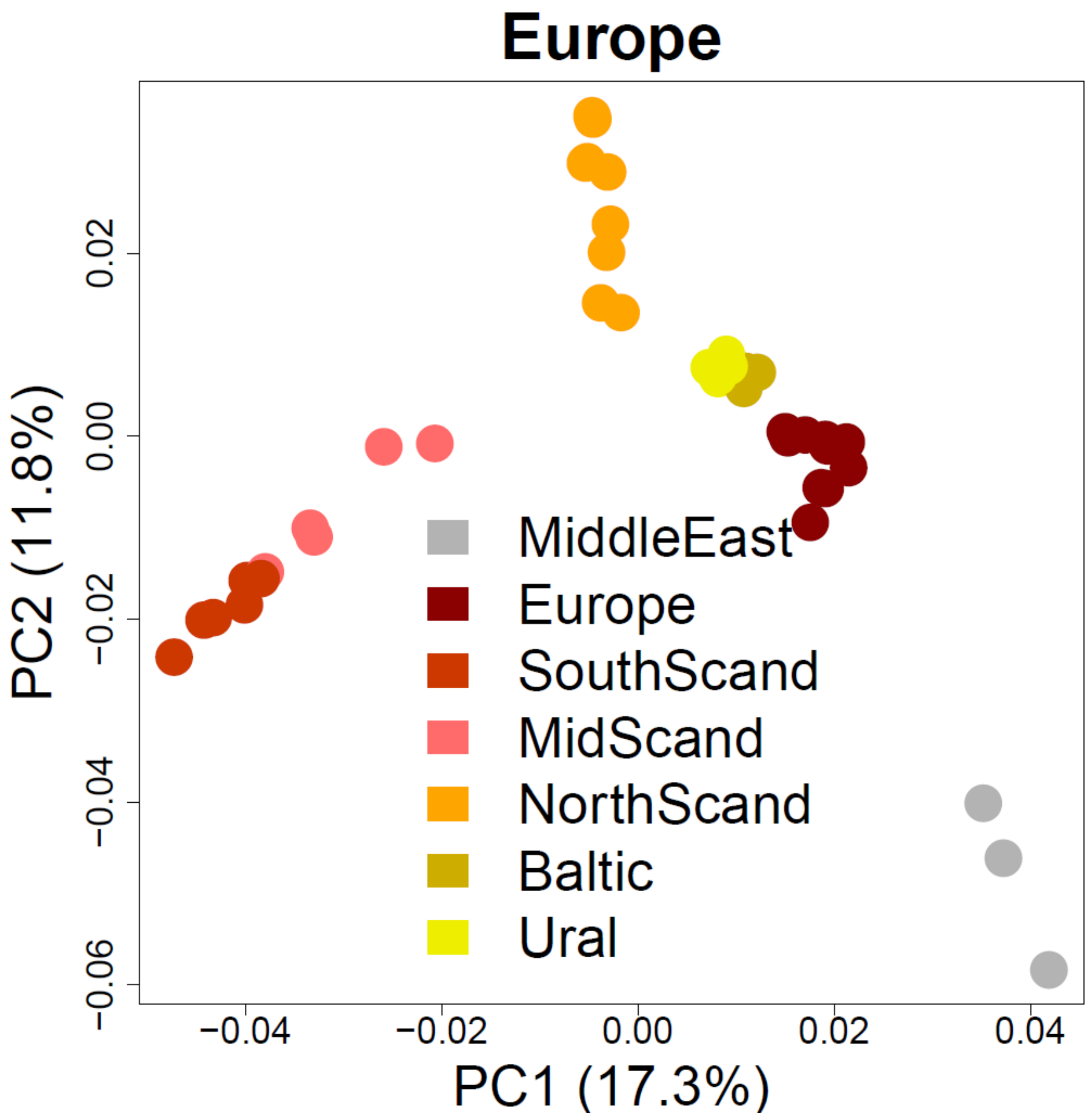

Fig. S2A. PCoA-plot showing genetic structure in western Eurasia. Biplot showing the first two of principal coordinate analyses (PCoA) based on allele sharing distances for the subset of western Eurasian samples.

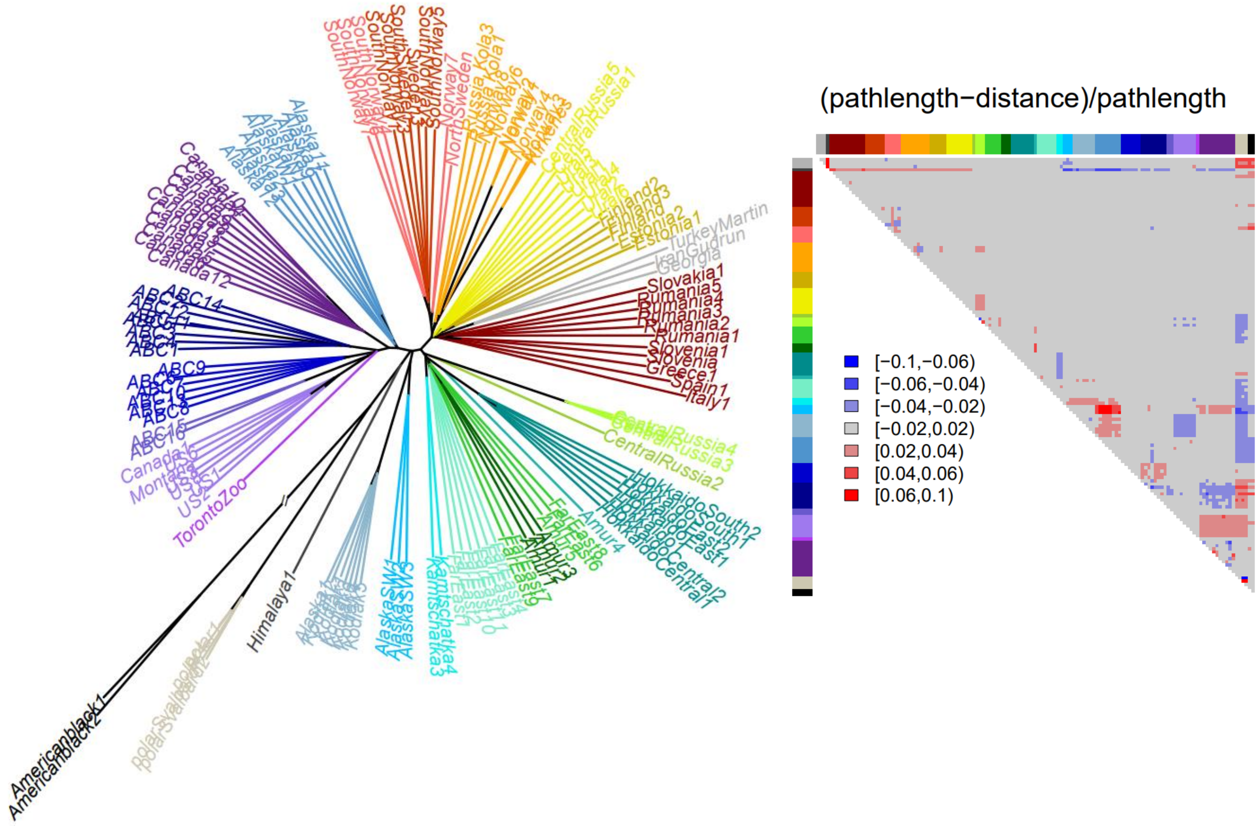

Fig. S2B. Hierarchical clustering (OLS algorithm) of Euclidean distances for autosomal dataset, and residual heatmap showing discrepancy between path lengths in dendrogram (fitted model) and actual genetic distance in underlying distance matrix (observations). Samples from the Middle East ('TurkeyMartin', 'IranGudrun' and 'Georgia') are clustered together with samples from Europe, and as a result they appear more distant from the Himalaya sample (and polar bears and black bears) then they actually are (as indicated by the heatmap).

The advantage of decoupling the Middle East samples from the Himalaya sample, is that indirectly allows polar bears to cluster more closely with North American brown bears. This greatly reduces the observed discrepancy for these population pairs between true genetic distances and path lengths in the bioNJ dendrogram (see main figure 1E-F). The reason that the discrepancy is not fully eliminated is that, relative to other eastern Eurasian bears, Amur and Hokkaido bears prefer to cluster closer to polar bears, pulling polar bears away from North American bears.

The clustering of bears Kodiak and Aleutian bears also causes unsolvable tension. Due to their insular isolation, Kodiak Island bears have not exchanged migrants with other North American populations. However, the sister population of coastal bears in southwestern Alaska ('Aleutian') was involved in gene flow events, causing them wanting to cluster one the hand with Kodiak bears, but on the other hand with other North American populations. This also explains why Kamchatkan bears, which likely derive from the same Beringian population, are attracted towards Kodiak and Aleutian bears.

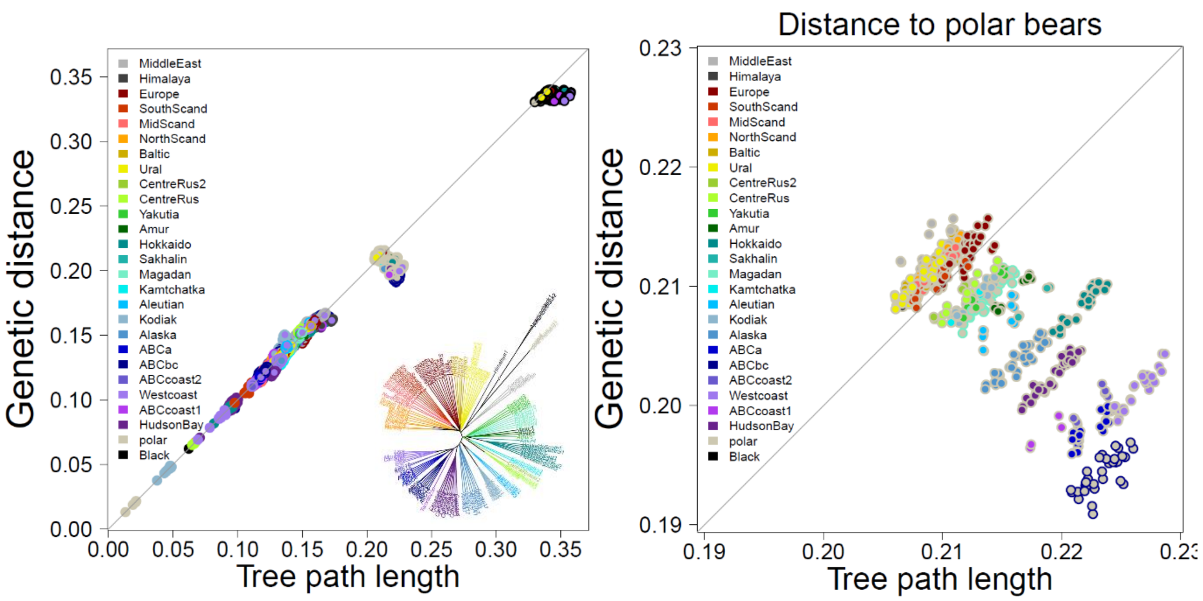

Fig. S2C. Hierarchical clustering residuals: discrepancy between path lengths in hierarchical structuring dendrograms (model) and actual genetic distance in underlying distance matrix (observations) for polar bear and brown bear comparisons. When polar bears are clustered with brown bears from Himalaya and the Middle East (inset, main figure 1F), the path lengths suggested by the dendrogram are overestimates of the actual genetic distances to eastern Eurasian and especially North American and Hokkaido brown bears.

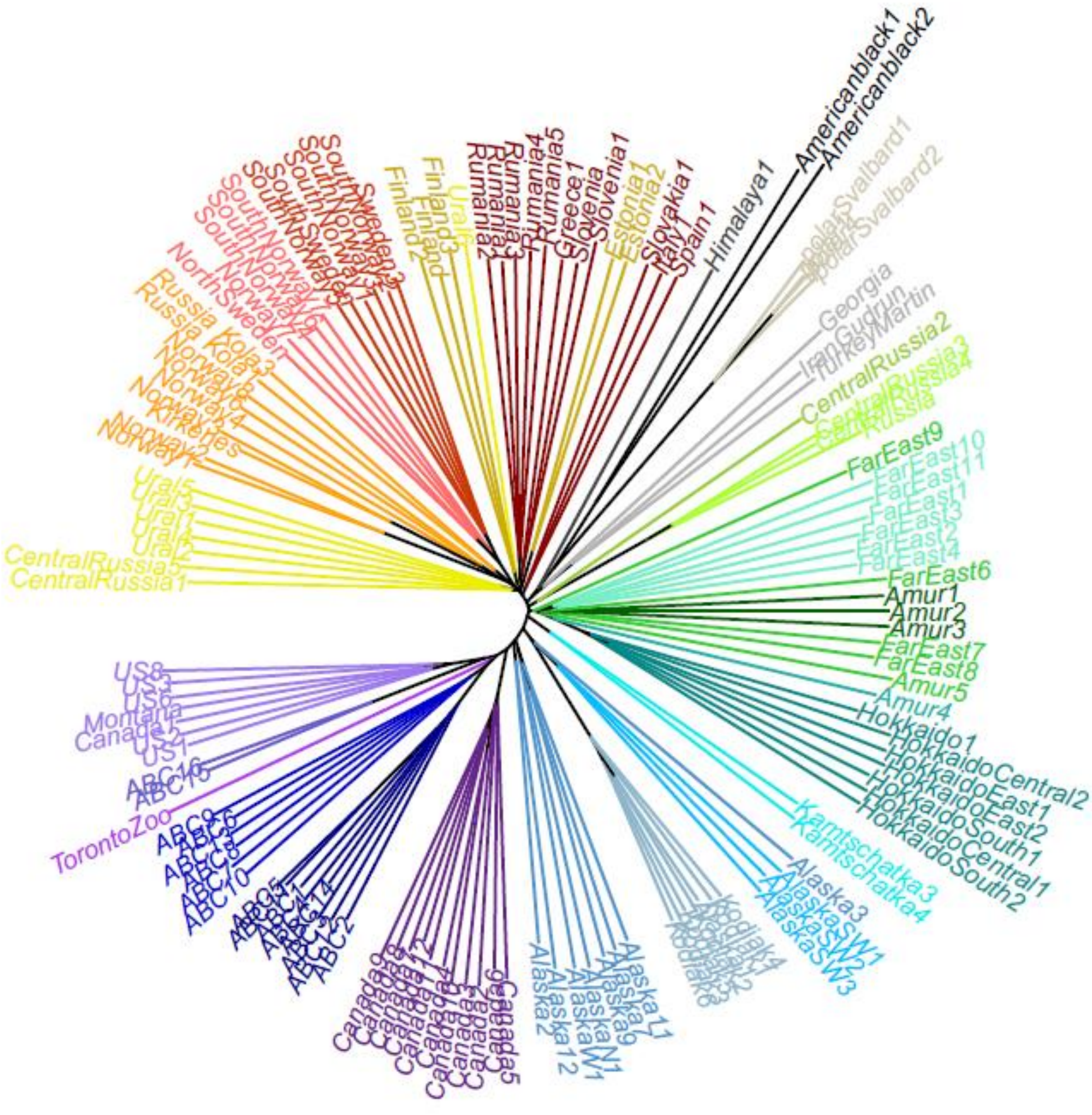

Fig. S2D. Microsatellite dataset bioNJ tree. Biological neighbour joining tree based on allele sharing distance for a dataset of >3000 tetranucleotide microsatellites. Not included are individuals with more than 70% missing data. The observed dendrogram is roughly similar to the SNP-based dendrogram (Fig. 1F).

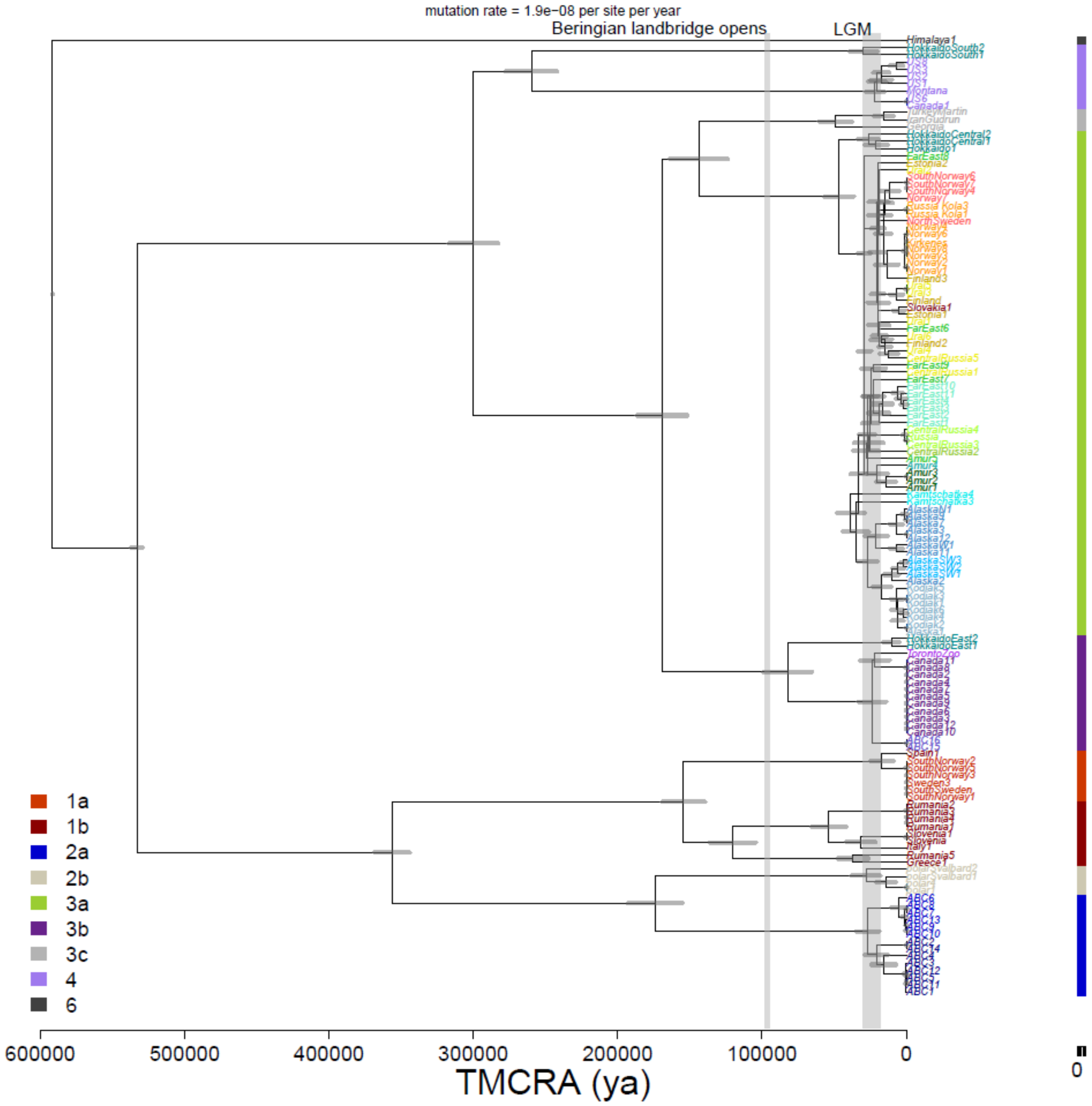

Fig. S3A. Mitogenome maximum likelihood phylogeny. Maximum likelihood phylogeny, generated with the software IQtree, using as input a whole mitogenome alignment (15194 bp) of 135 brown bear individuals. The phylogeny has been linearised using the chronoMPL function of the R package 'ape'. Genetic distances are converted into TMCRA estimates assuming a mutation rate of $1.9*10^{-8}$ per site per year. The clustering agrees with previously published results, and separates out mt-DNA haplotype clusters 1b (Europe), 1a (Iberia and southern Scandinavia), 2a (ABC bears), 2b (polar bears), 3a (Eurasia, Alaska and central Hokkaido), 3b (Canada and southern Hokkaido) and 4 (North American westcoast and eastern Hokkaido). The TMCRA estimates indicate deep divergence times between all major MT haplotype clades, predating the Last Glacial Period, implying that incomplete lineage sorting must have contributed to the current geographical distribution of these haplotypes.

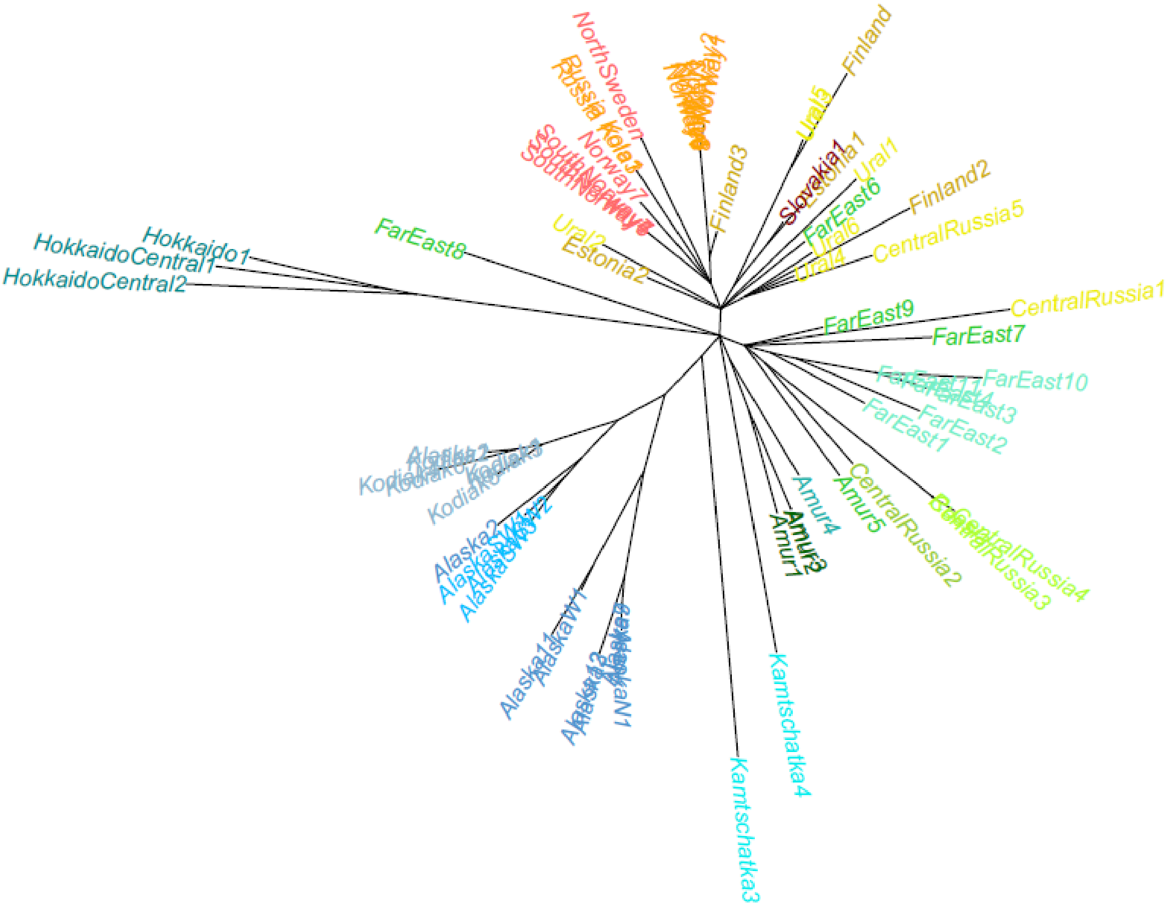

Fig. S3B. Mitogenome maximum likelihood phylogeny (mtDNA-clade 3a). Unrooted maximum likelihood phylogeny, generated with the software IQtree, using as input a whole mitogenome alignment (15194 bp) of 135 brown bear individuals. Shown is a subset of individuals carrying haplotype 3a.

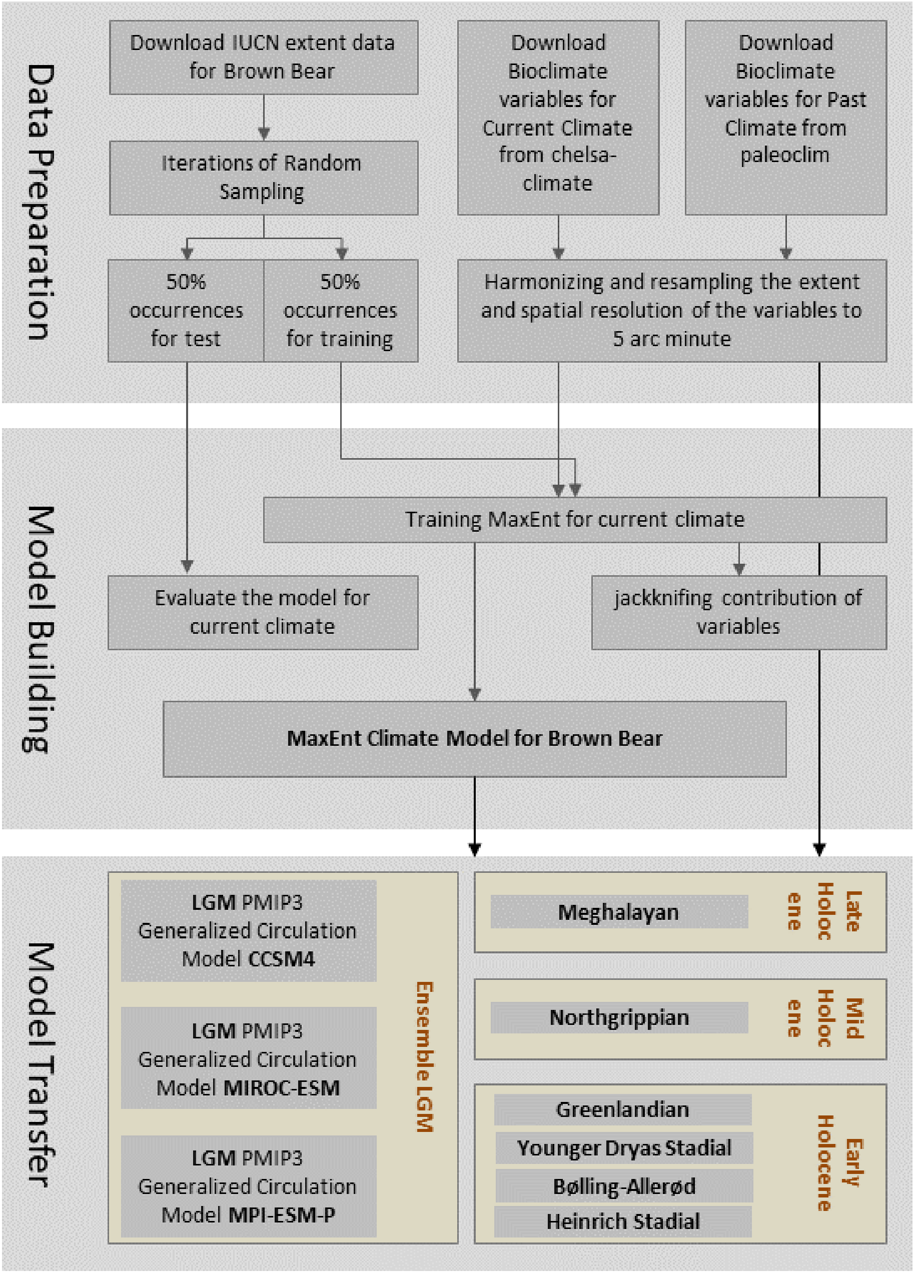

A flow chart describing the key steps of the species distribution models.

Fig. S4A. Climate suitability modelling workflow.

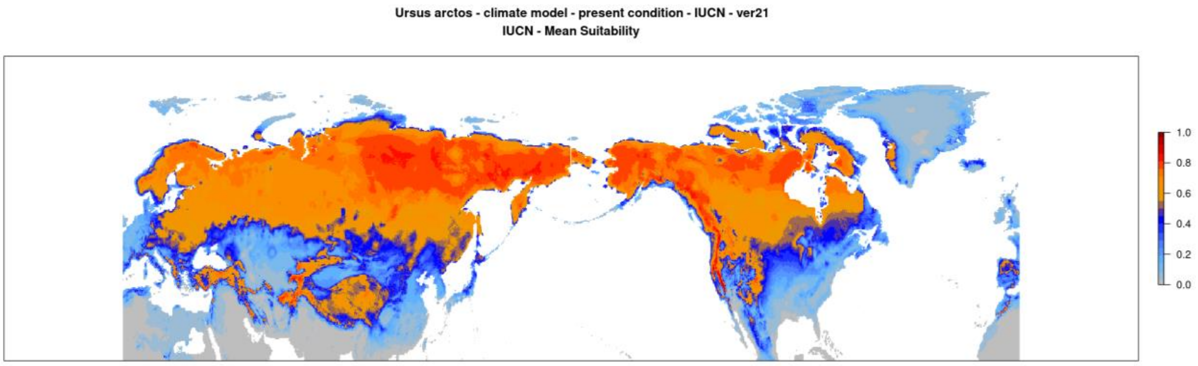

Fig. S4B. Climate suitability modelling. Present-day brown bear climatic suitability inferred by climate suitability modelling.

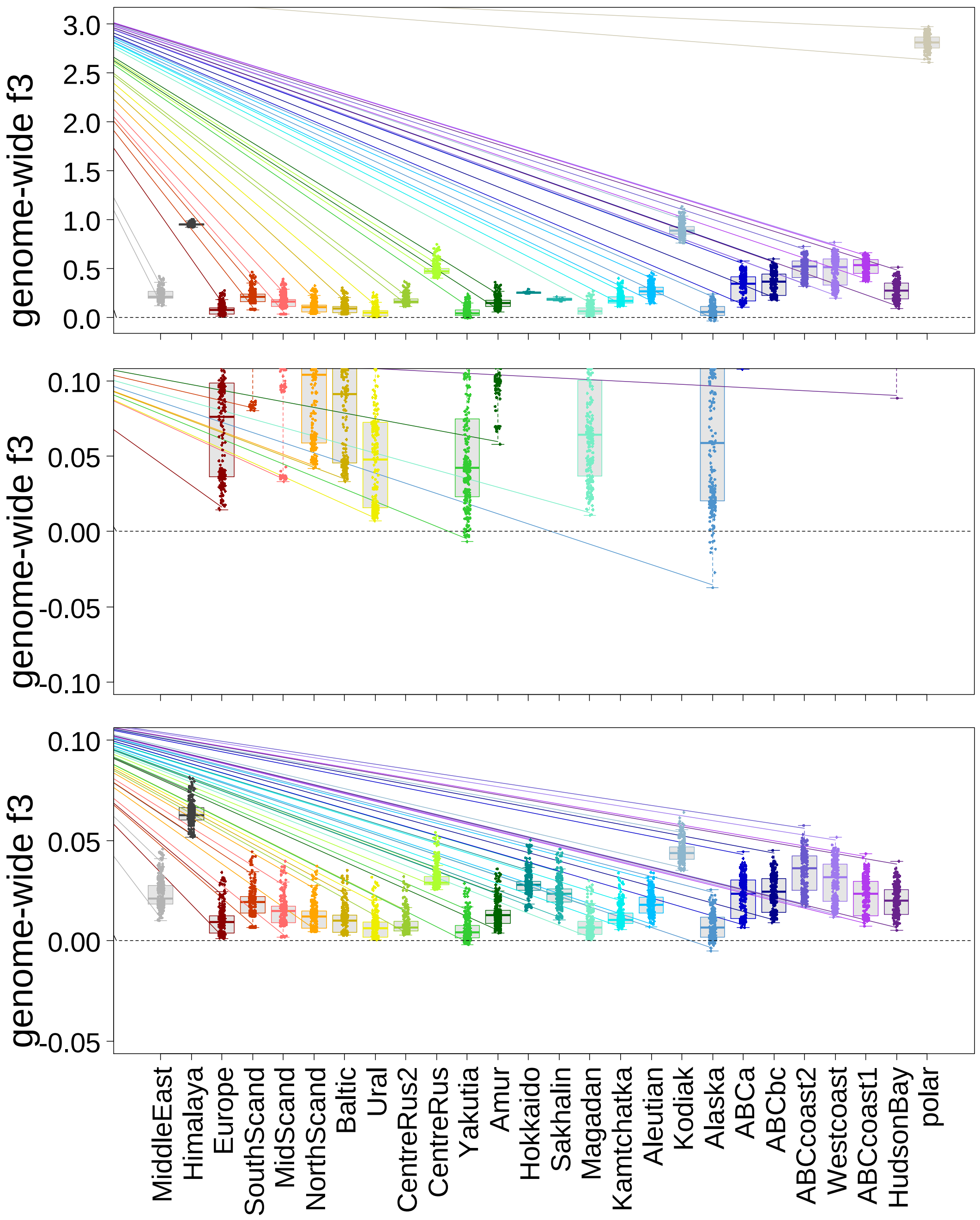

Fig. S5A. Admixtools f3-statistics. Genome-wide mean f3-score a.) calculated on a sliding window-basis per 50kb window and summarized as the overall mean of all windows combined (top/middle; note different scales of y-axis) and b.) calculated over a subset of 40K SNPs (bottom). Shown are all possible population triplets (X;Y,Z). (Note that from these boxplots it cannot be inferred which population pairs are the putative ancestral populations.) Negative f3-scores indicate that allele frequencies in population X are intermediate of allele frequencies in population Y and Z, which is suggestive of ongoing or past admixture. Both datasets indicate that negative f3-scores are found only for population triplets in which Yakutia and Alaska represent the putatively admixed population X. The window-based mean was calculated over all windows excluding windows with a f3-score below -5 or above 5.

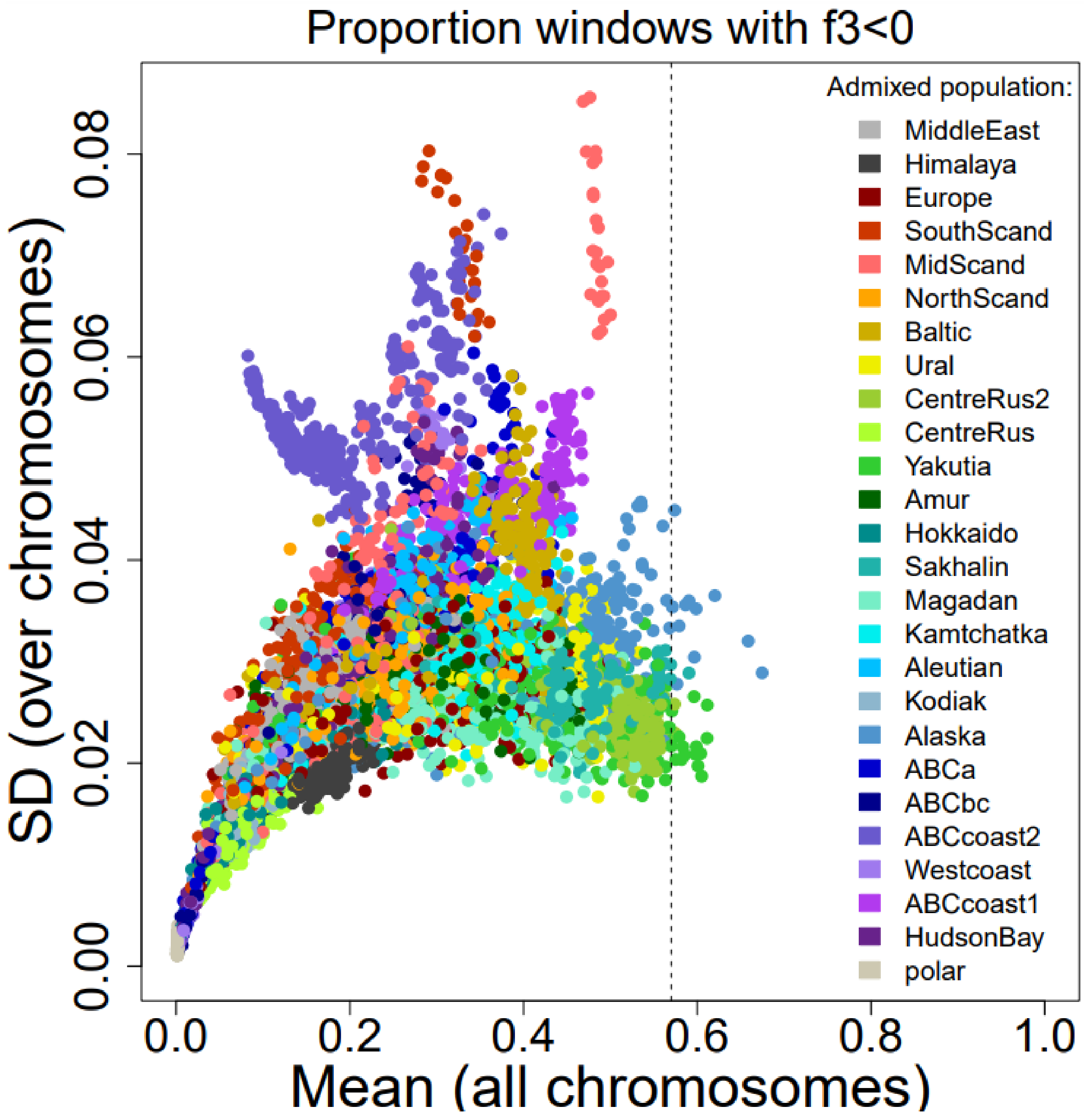

Fig. S5B. Admixtools f3-statistics. Genome-wide mean f3-score for each population triplet (X;Y,Z), calculated on a sliding window-basis per 50kb window, with on the x-axis the proportion with a f3-score below 0, and on the y-axis the standard deviation of this proportion across chromosomes. Colours denote population X. Approximately 50% of the windows of all population triplets (MidScand; SouthScand, Z) have negative f3-values, with, for unknown reasons, relatively high variation of these proportion across chromosomes. The dashed vertical line represents the arbitrary cut-off value used when generating main Fig. 4B.

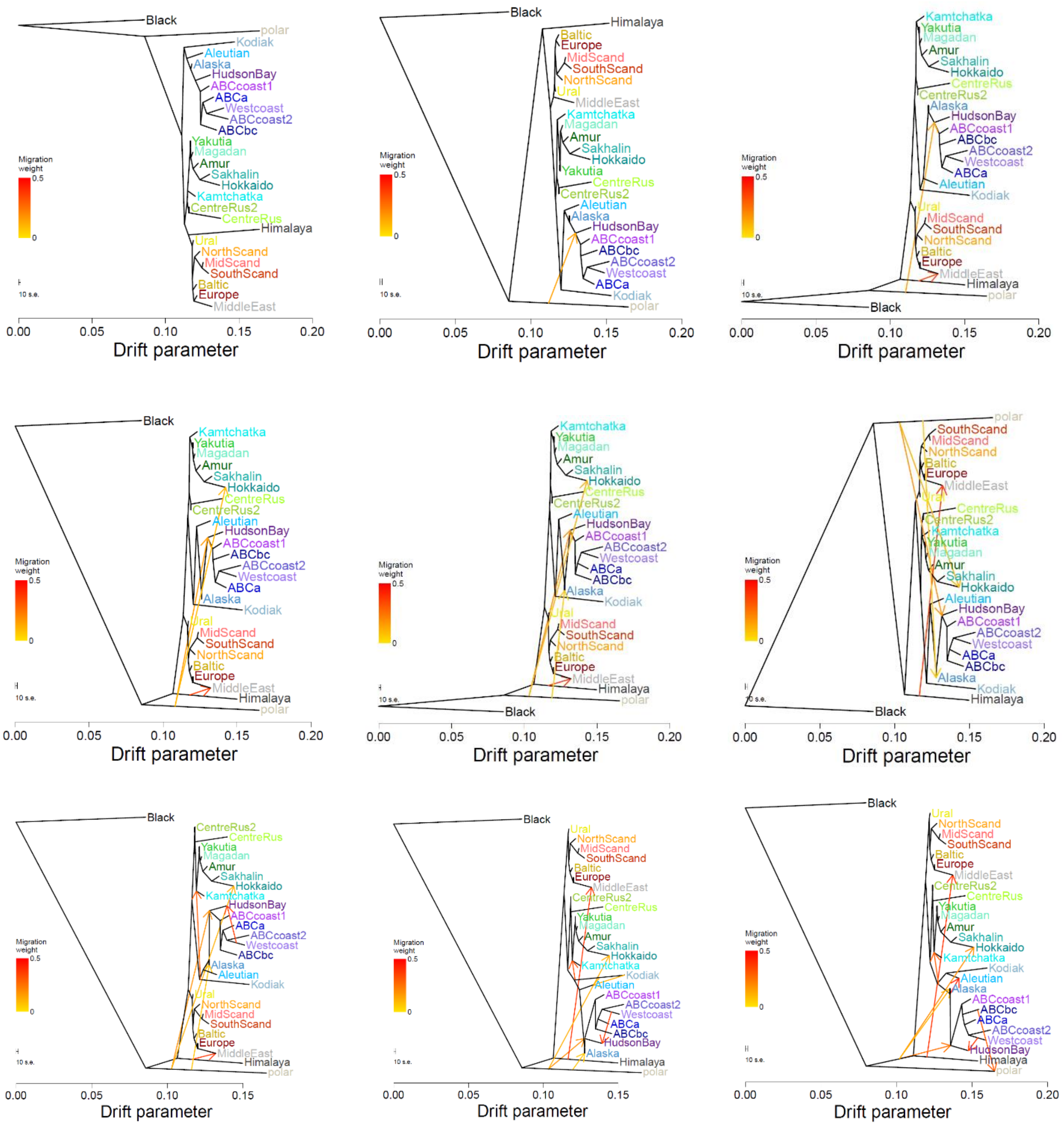

Fig. S5C. Treemix maximum likelihood phylogenies, with varying number (0-8) of migration edges. Treemix admixture graphs for 80K SNP dataset, with varying number of migration edges.

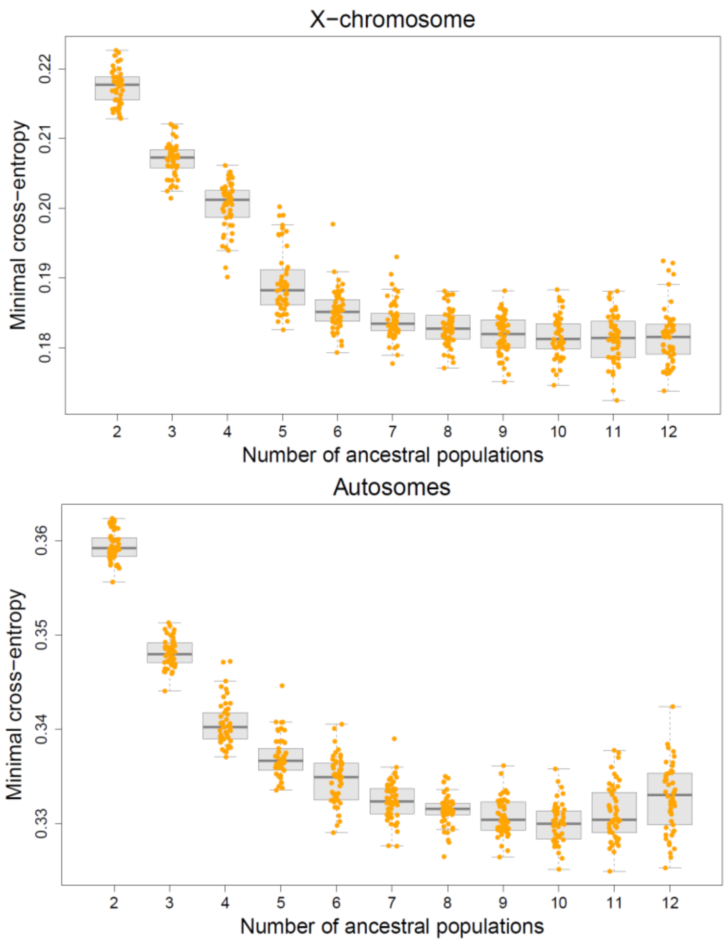

Fig. S6A. Determination of optimal K using cross-entropy score criterion. Shown are minimal cross-entropy scores, generated with the function 'snmf' of the R package LEA, obtained for 50 independent runs. Based on the 'elbow method', according to which the optimal K is the start point of a plateau, the optimal K-value is 6 or 7, both for the X-chromosomal and autosomal dataset.

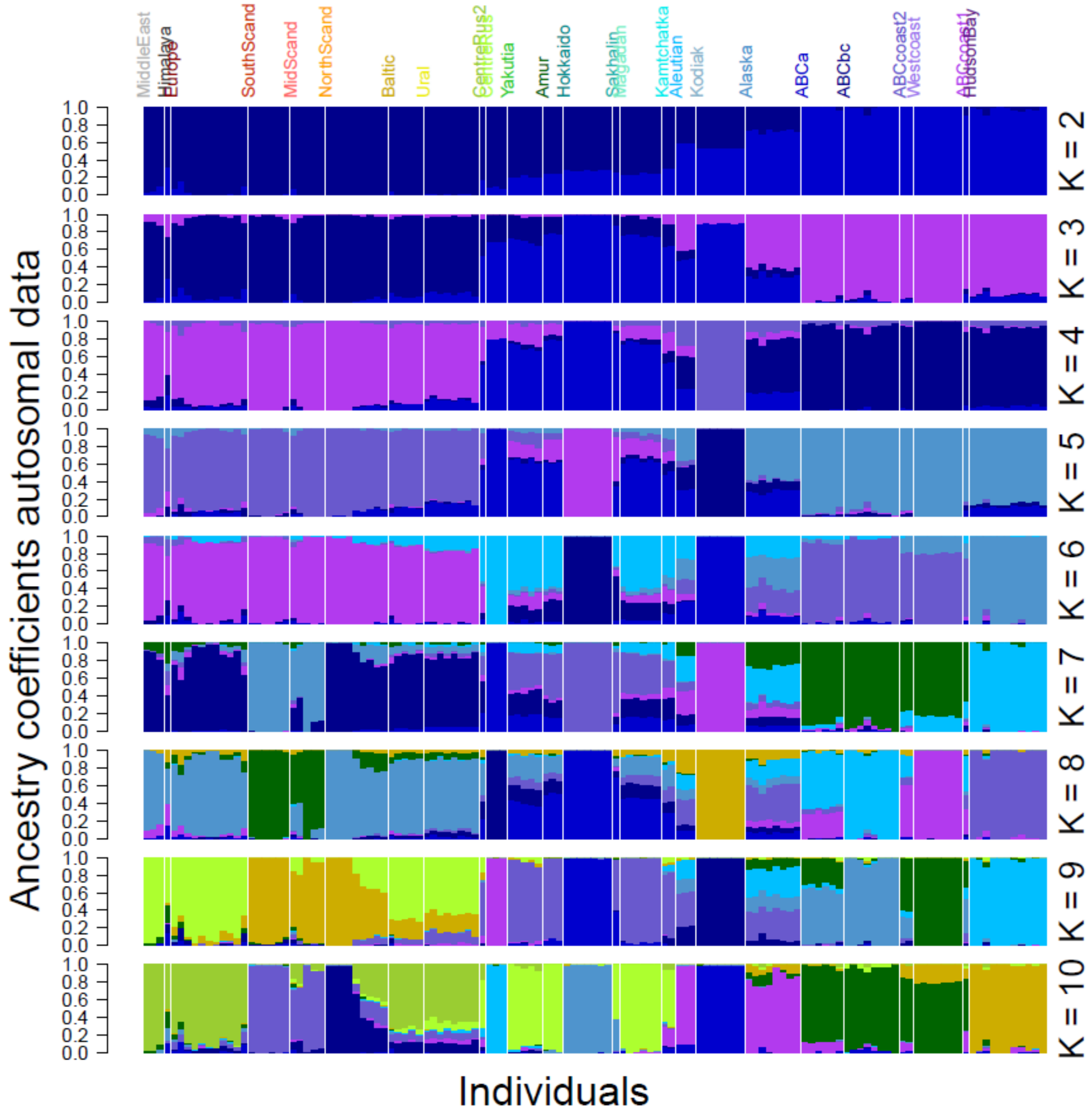

Fig. S6B. Admixture plots, generated with R package LEA, for autosomal dataset.

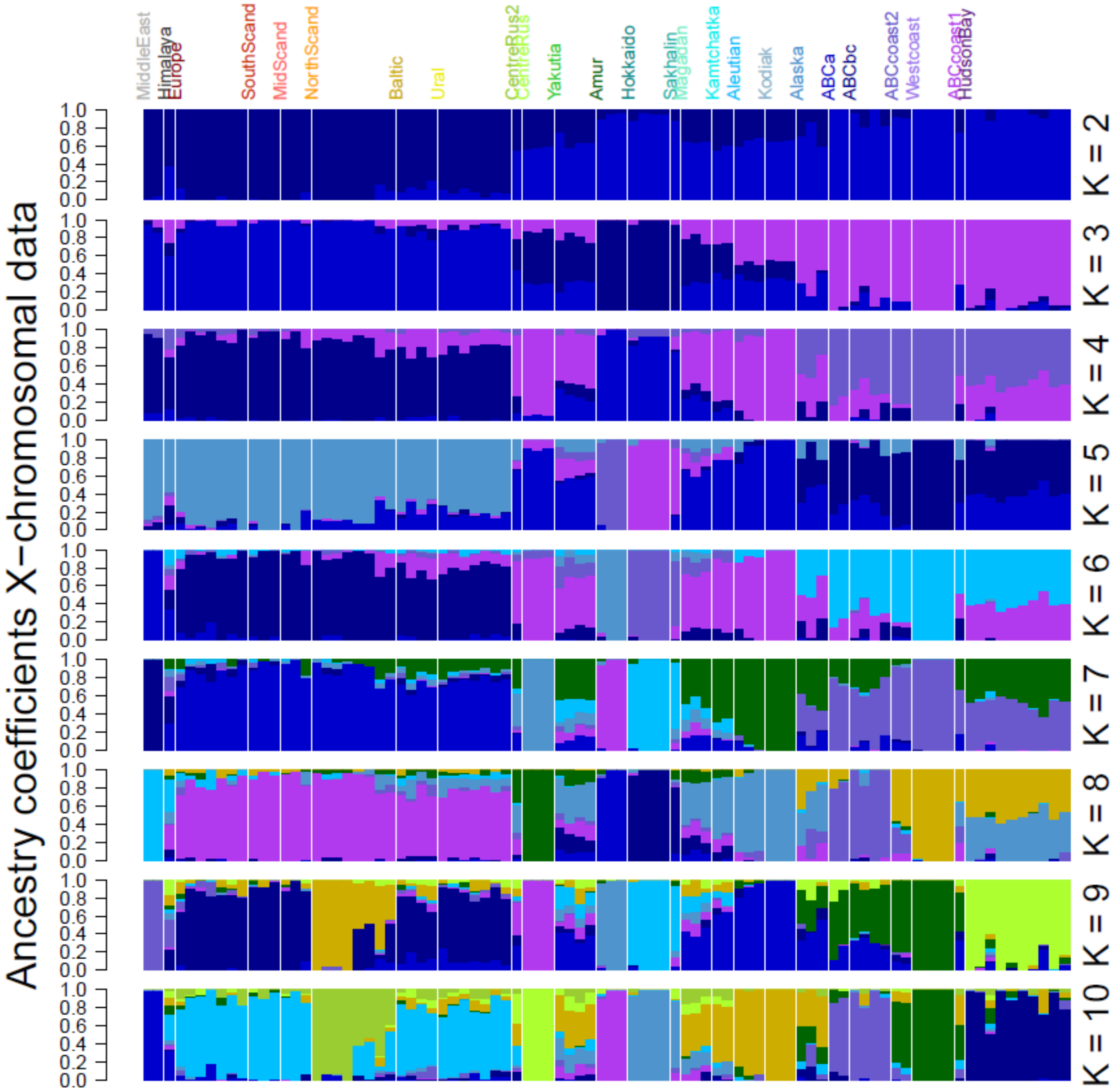

Fig. S6C. Admixture plots, generated with R package LEA, for X-chromosomal dataset (males only).

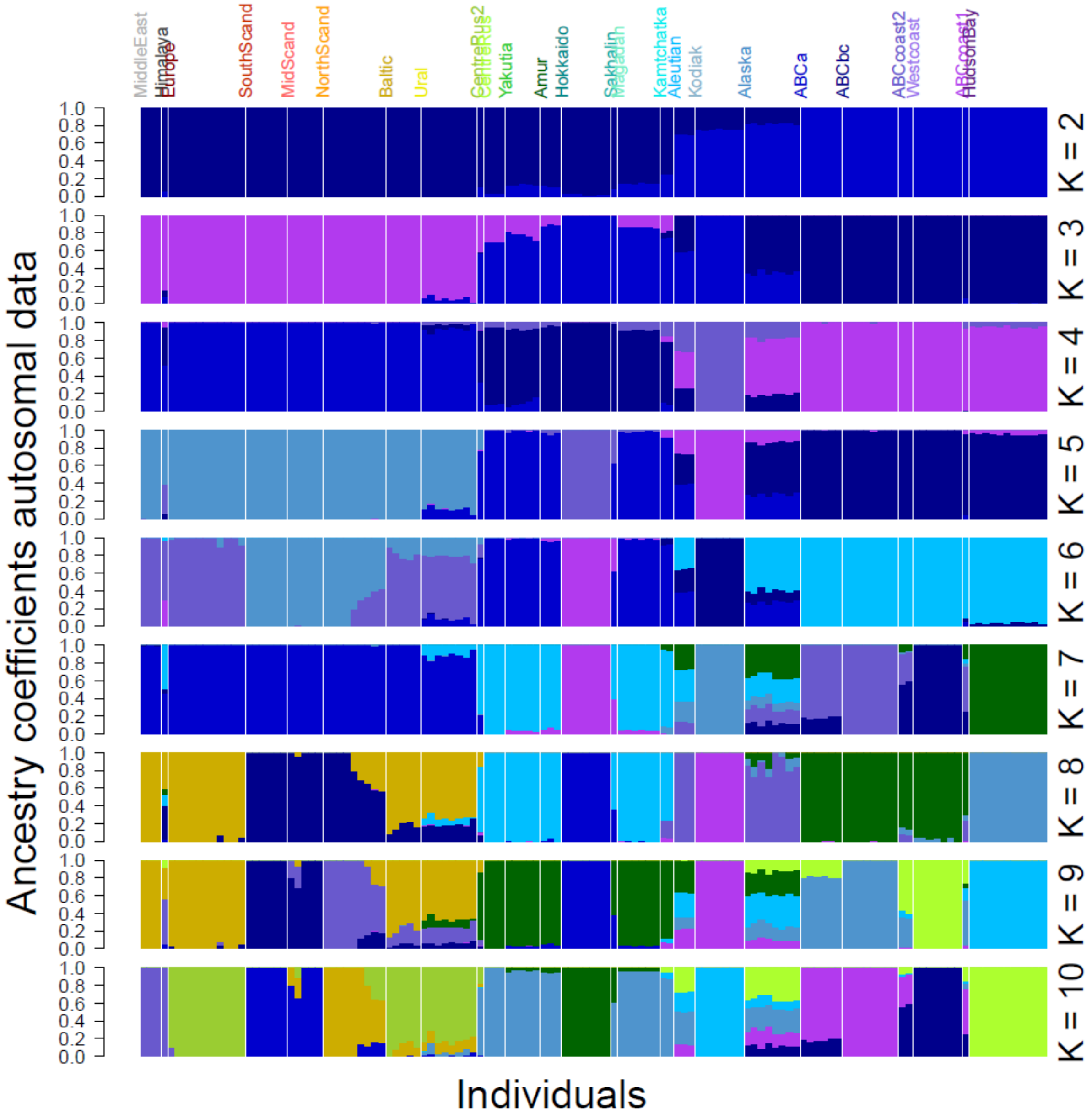

Fig. S6D. Admixture plots, generated with Admixture software, for autosomal dataset.

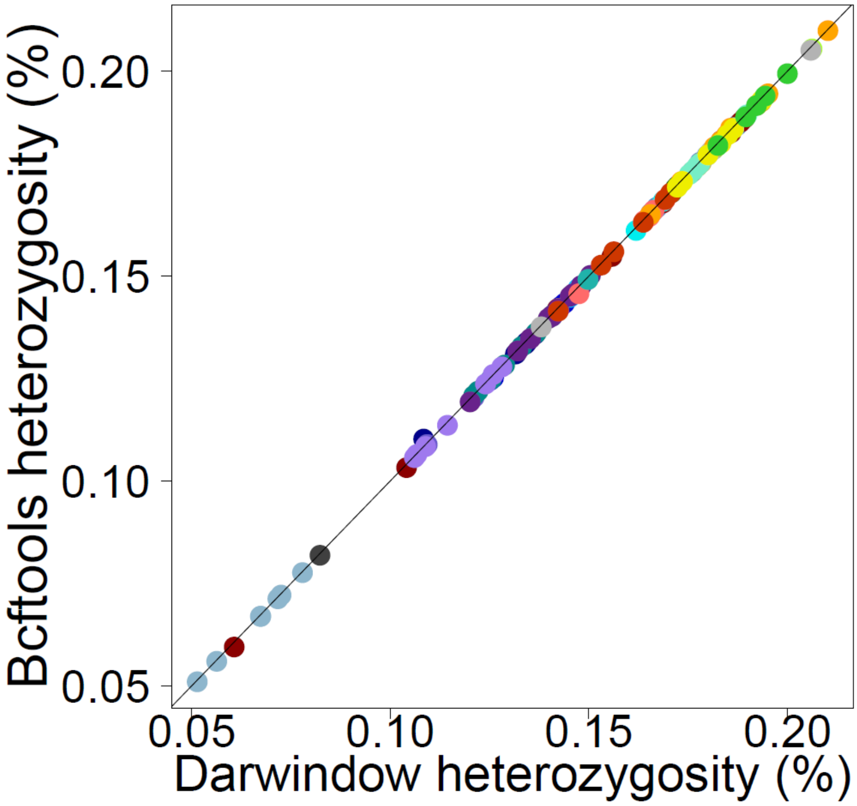

Fig S7A. Genome-wide heterozygosity (He): Darwindow vs bcftools. Comparison between genome-wide heterozygosity values estimated with Darwindow (x-axis) and with bcftools stats -s – (y-axis). Each dot represents a sample. The diagonal indicates x=y. Prior to the heterozygosity calculations, indels have been removed using the command 'zgrep -v 'INDEL' data.vcf.gz | gzip > data.noindels.vcf.gz'.

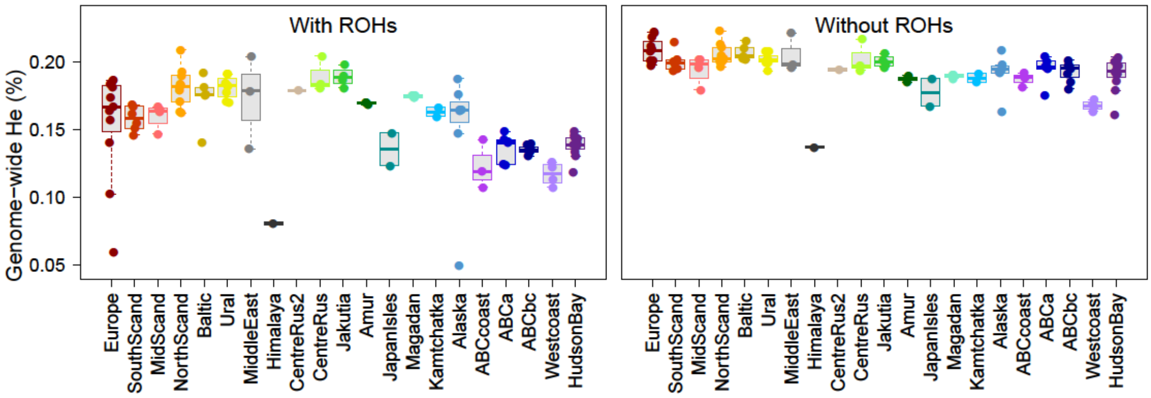

Fig S7B. Genome-wide heterozygosity (He) before and after exclusion of ROHs. Genome-wide sample specific heterozygosity estimates (proportion of heterozygous sites) measured over all sites (left) and measured over sites which do not overlap with regions in the genome marked as run of homozygosity (right).

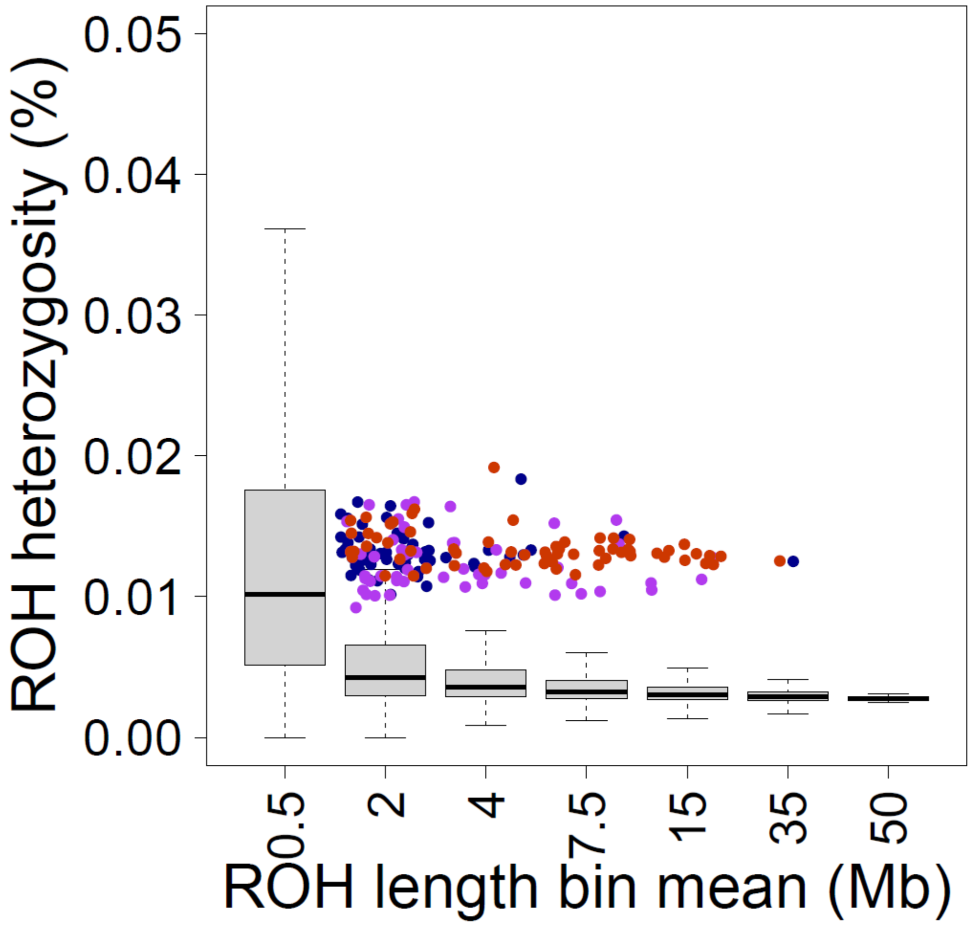

Fig S8. Heterozygosity (He) levels within ROHs. Consistent with expectations, short ROHs have slightly higher heterozygosity levels (because shorter ROHs derive from a more distant-in-time inbreeding event, and therefore had more time to accumulate mutations). Mean He-levels are above expected levels (2ut), suggesting genotype errors. Boxplots shows the distribution of values for all samples combined. The dots show the values observed for three erratic samples (unknown cause).

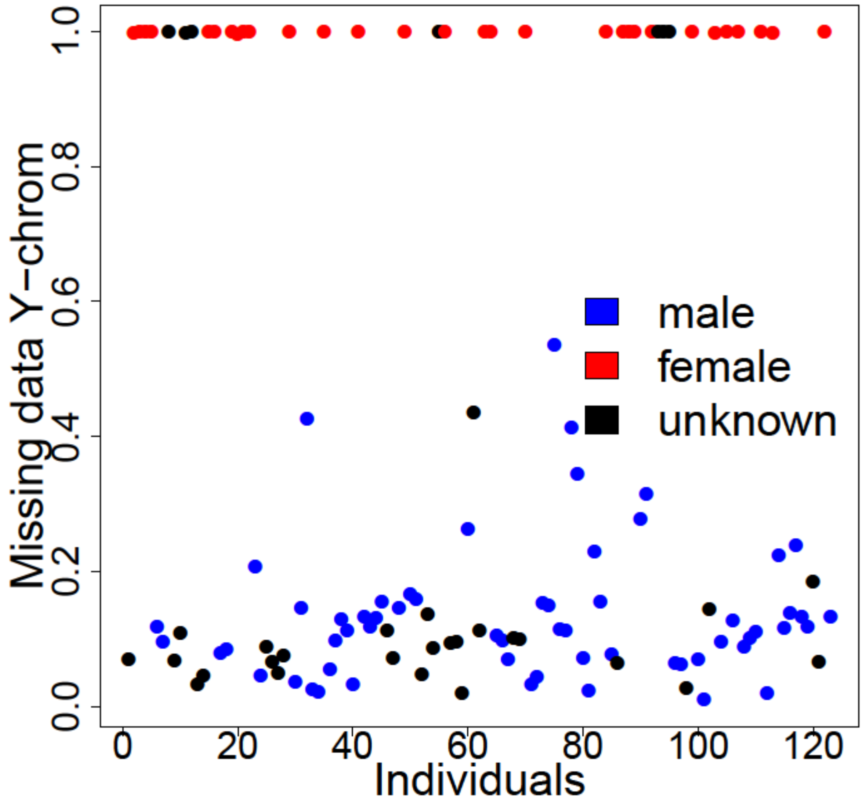

Fig. S9. Sex determination based on levels of missing data at the Y-chromosome. For the subset of samples without gender information, the gender was determined from levels of missing data observed at the Y-chromosome. These values were derived by mapping the raw reads of all 123 samples against HiC_scaffold_38 of the DNAzoo brown bear reference genome, assuming for each sample a ploidy of 1 (i.e., bcftools call –ploidy 1). We masked genotypes with a depth below 3, and subsequently discarded sites with a global depth below 400 or above 1000, as well as sites with information for less than 70 (out of the total 123) genotypes.

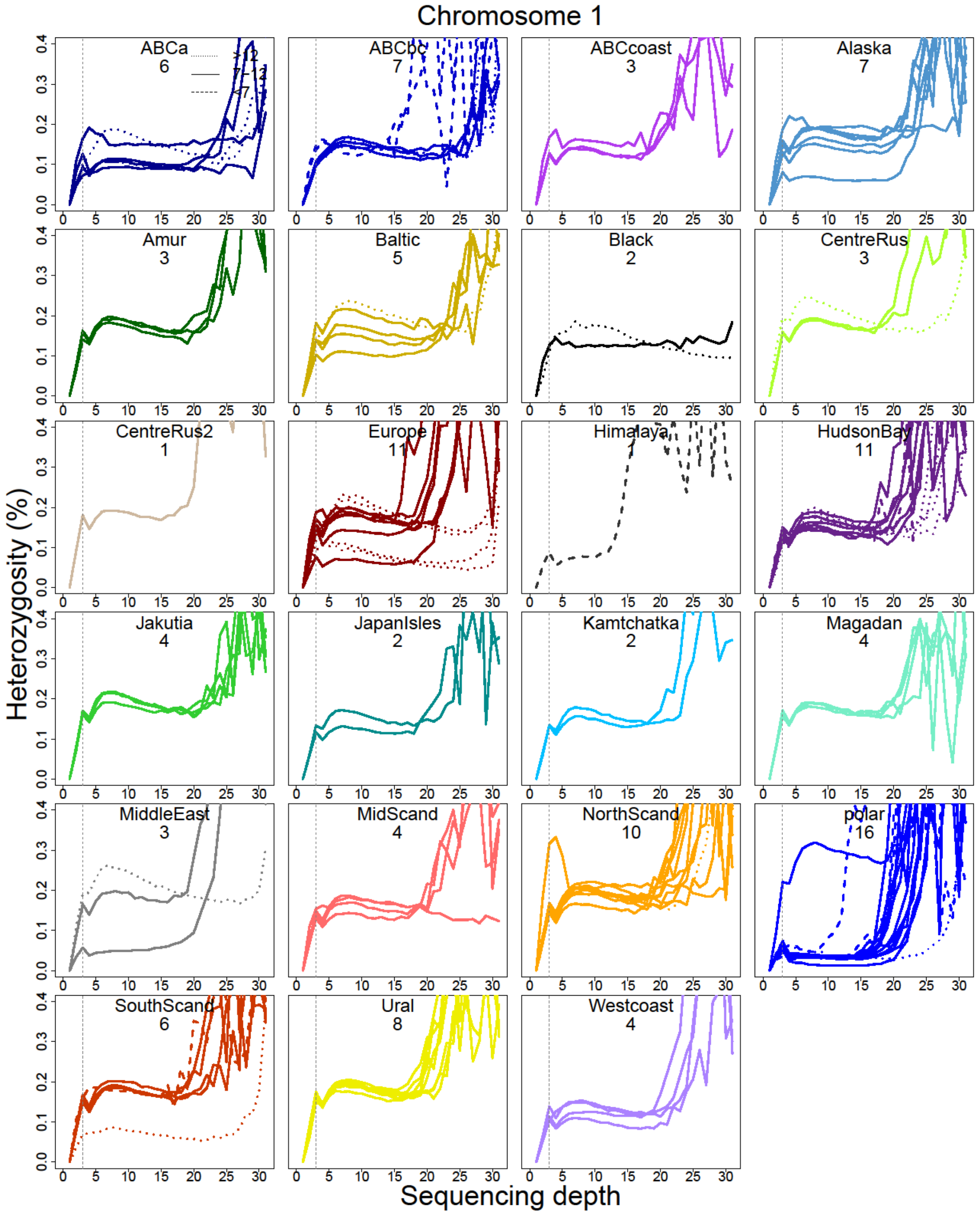

Fig. S10A. Heterozygosity as a function of read depth, when inferring sample genotypes <u>NOT</u> considering information from other samples.

Shown are heterozygosity values for sites binned based on sequencing depth. Dashed, solid and dotted curves indicate genomes with a mean depth of respectively <7, 7-12, and >12. Dashed vertical lines represent the masking cut-off: sites below a read depth of 3 have been setting missing for the respective sample. The numbers below the population names represent the number of individuals per populations. The high heterozygosity value at high read depths (depth > 15) are due to paralogous loci. (Not shown are samples from the populations 'Aleutian' and 'Kodiak'. Samples from 'Sakhalin' and 'Hokkaido' (1x) are grouped within the population 'JapanIsles', and the populations 'ABCcoast1' and 'ABCcoast' are grouped together.)

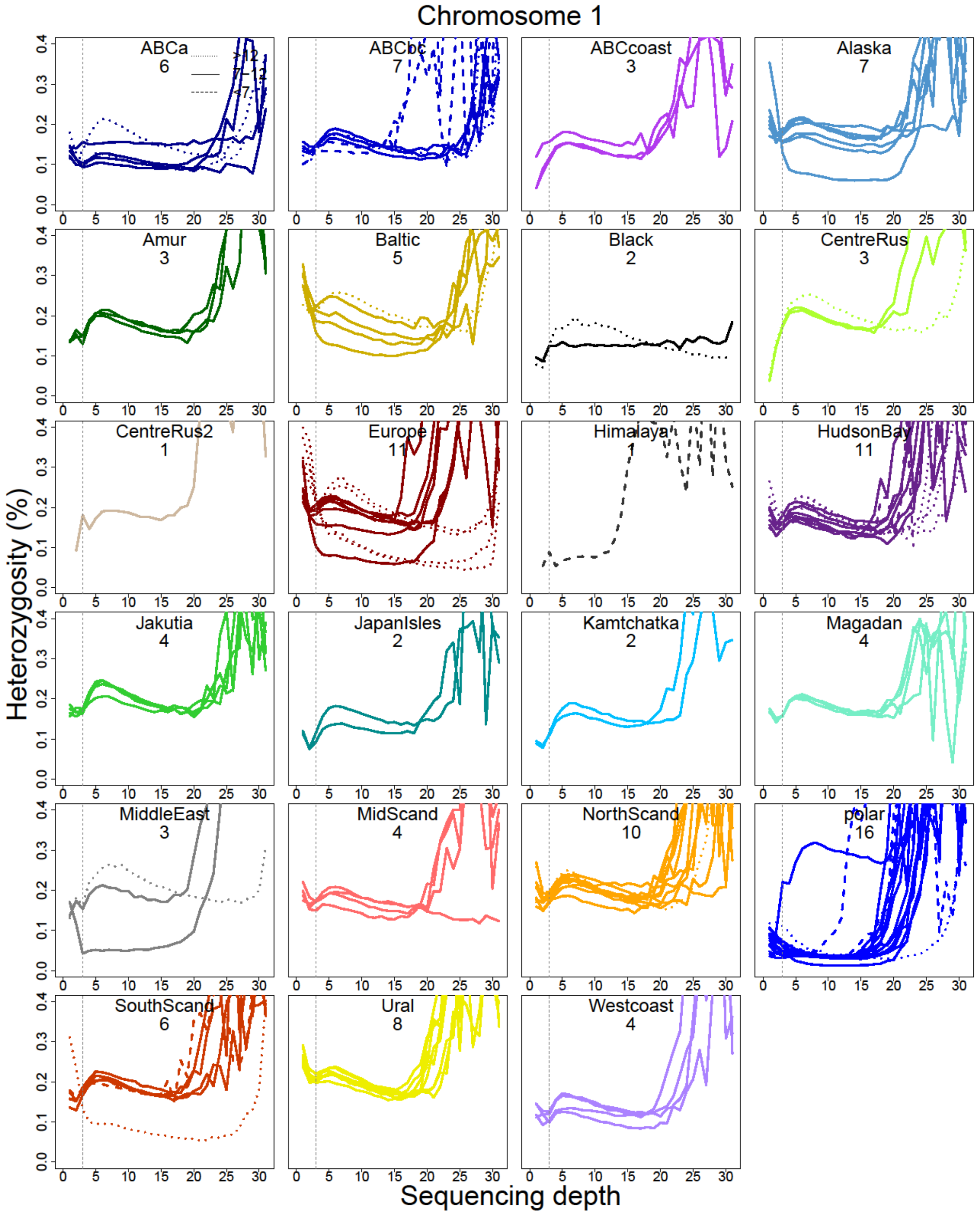

Fig. S10B. Heterozygosity as a function of read depth, when inferring sample genotypes considering information from other samples in <u>same population</u>.

Idem as S2A.

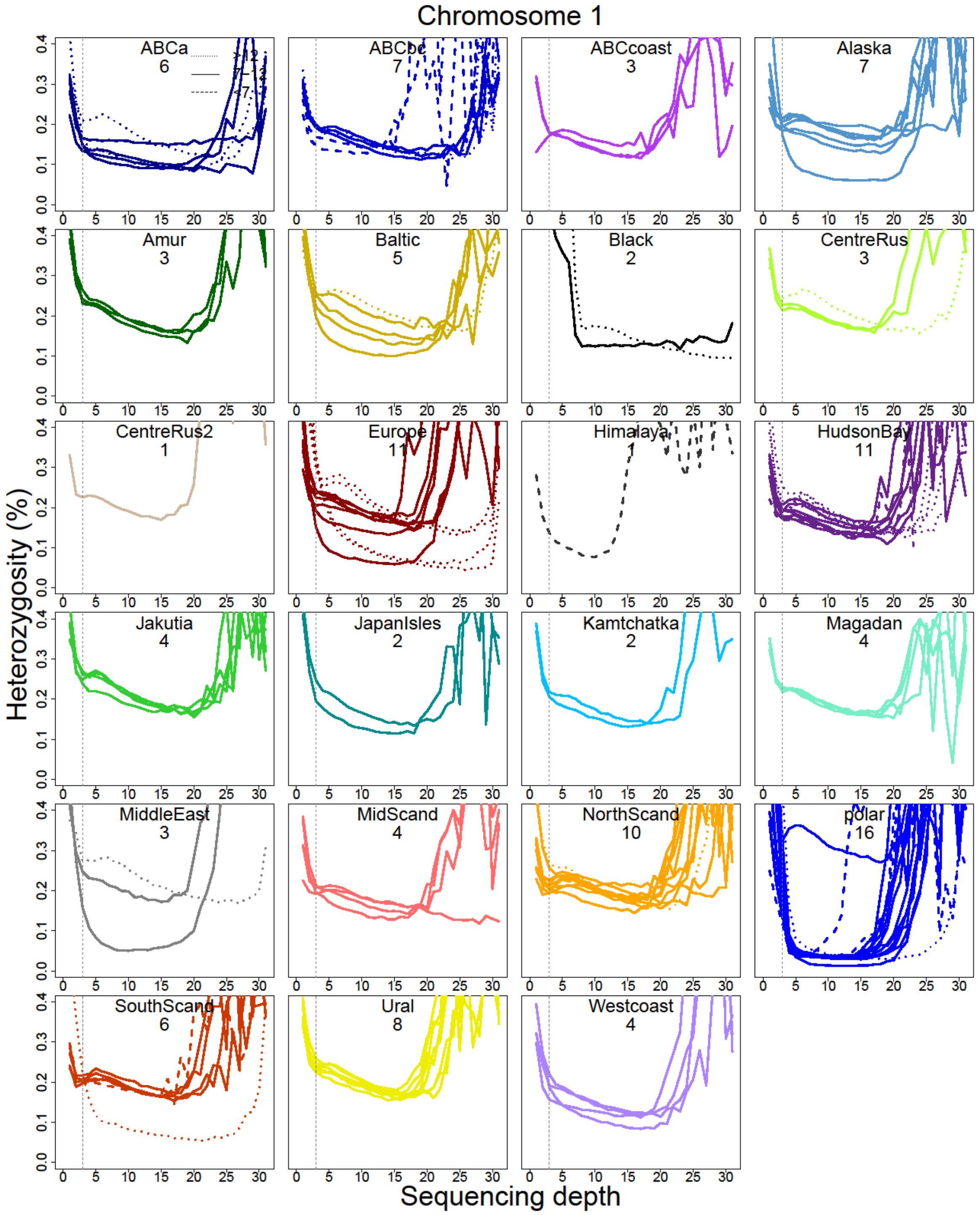

Fig. S10C. Heterozygosity as a function of read depth, when inferring sample genotypes considering information from <u>all</u> other samples in dataset.

Idem as S2A.

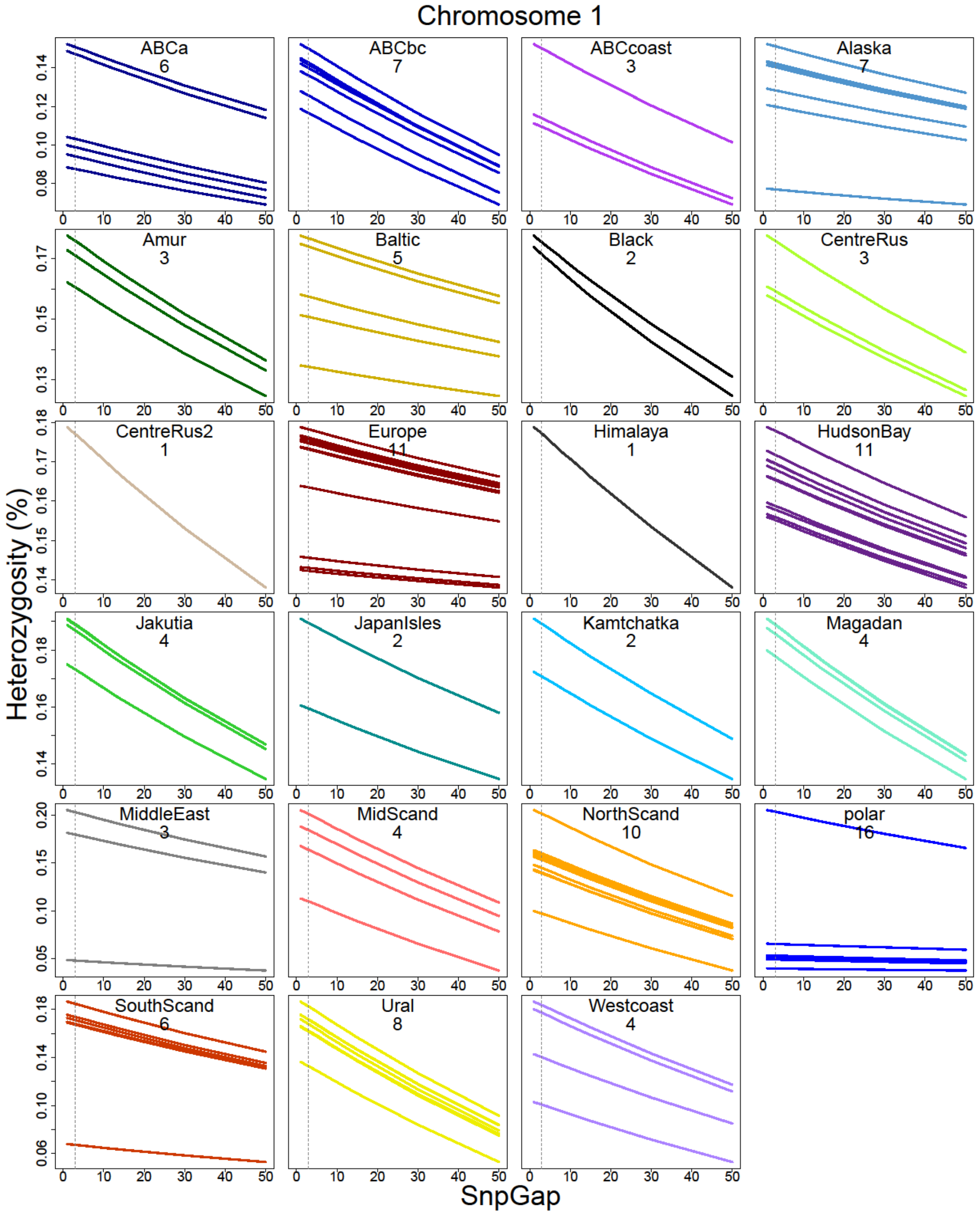

Fig. S11. Genome wide heterozygosity as a function of SnpGap setting. SnpGap, a setting of the bcftools call function, indicates the distance (in bp) between indels and heterozygous sites which are masked. Each line represents a single sample. If heterozygous sites occur in above average frequency close to indels, then we would not expect the near-linear relationships observed in the data. Therefore, we decided to set SnpGap to 0. (Not shown are samples from the populations 'Aleutian' and 'Kodiak'. Samples from 'Sakhalin' and 'Hokkaido' (1x) are grouped within the population 'JapanIsles', and the populations 'ABCcoast1' and 'ABCcoast' are grouped together.)

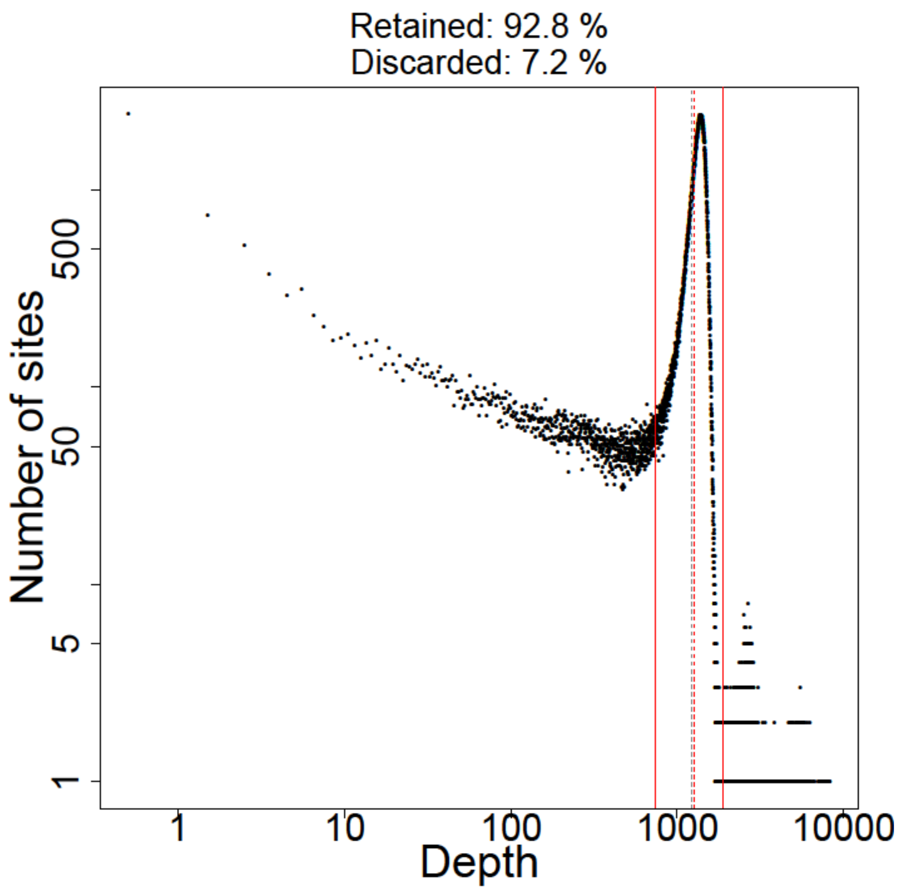

Fig. S12A. Overall depth per site. Distribution of overall depth per site (summed over samples) for a randomly thinned dataset. The red solid lines indicate the filter settings (min and max read depth per site).

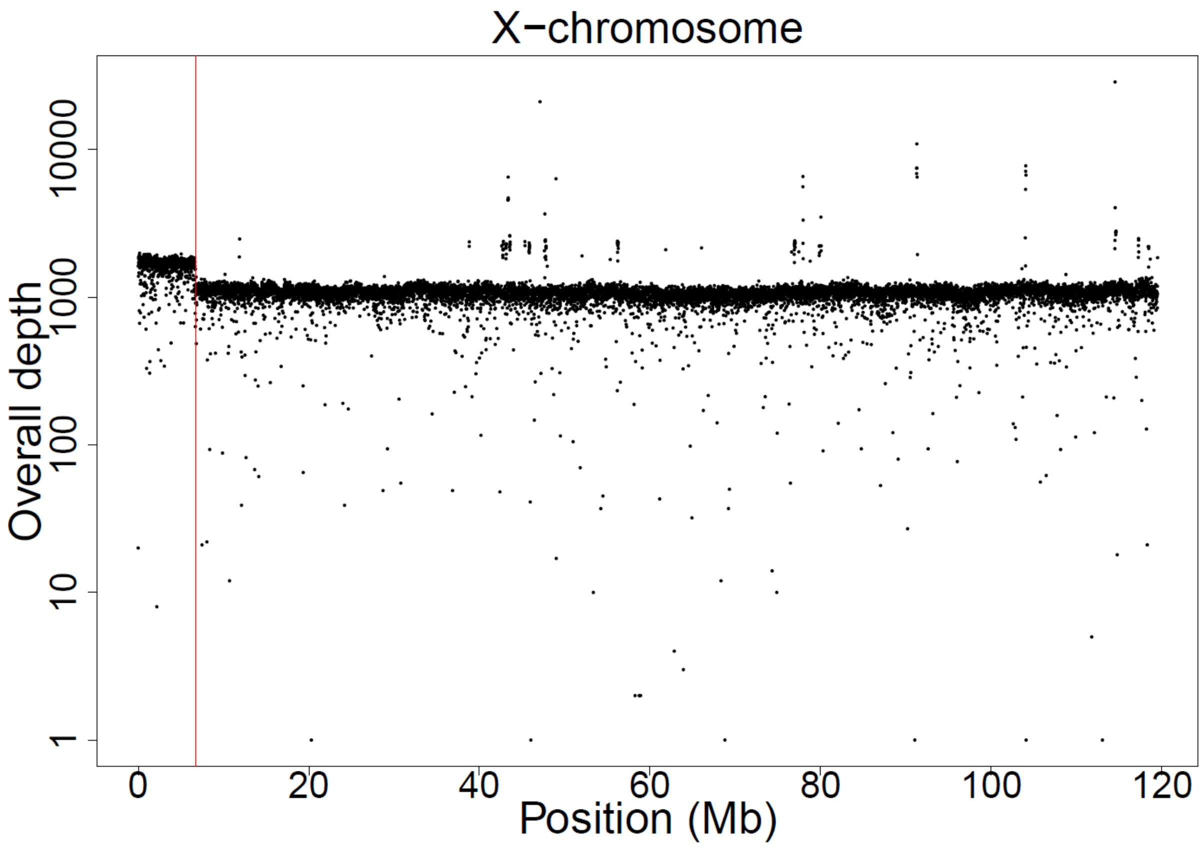

Fig. S12B. Identification of pseudo-autosomal region (PAR). Scatterplot depicting overall sequencing depth (all samples combined) across the X chromosome. The pseudo-autosomal (PAR) region (on lefthand side of red line) was identified based on sequencing depth, and removed from subsequent analyses. Assuming a mean depth of 10x, and given a dataset of 94 males and 41 females, the expected overall site depth is (135*10 =) 1350 for (pseudo-)autosomal regions, and (94*5 + 41*10 =) 880 for the majority of the X-chromosome.

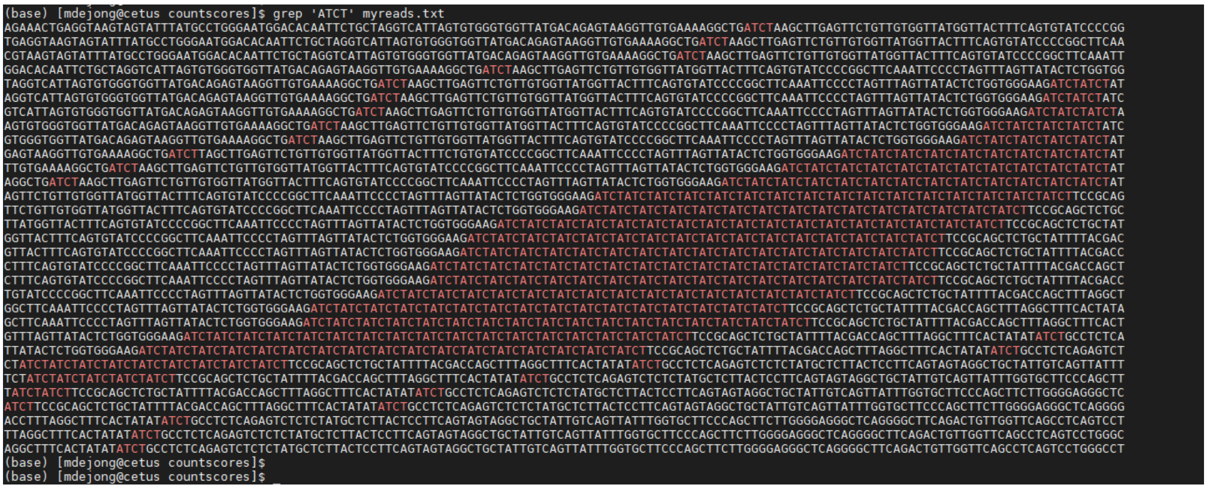

Fig. S13A. Microsatellite (VNTR) genotyping from short read sequencing data. Screen shot showing raw sequencing reads in a region contain the tetranucleotide repeat ‘ATCT’. Highlighted in red are all occurrences of ‘ATCT’.

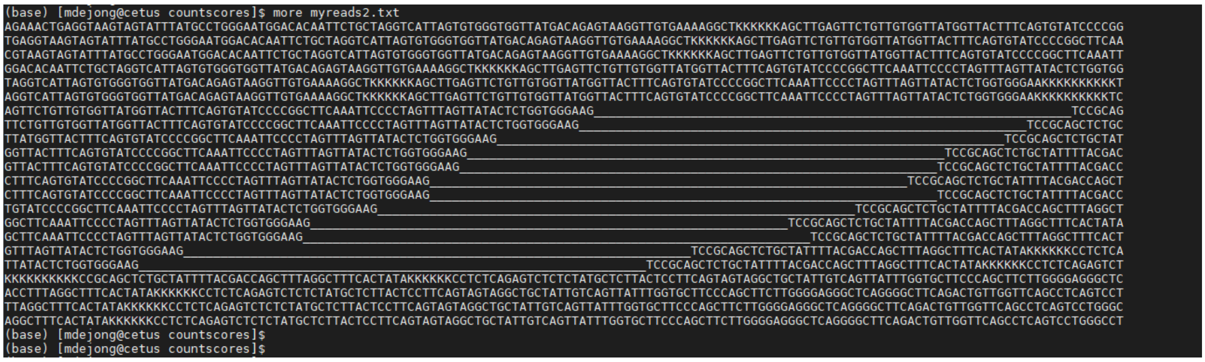

Fig. S13B. Microsatellite (VNTR) genotyping from short read sequencing data. Screen shot showing processed reads. Single occurrences of ‘ATCT’ have been masked with ‘KKKK’. Reads with truncated microsatellites have been discarded. The underscores highlight the retained microsatellites.

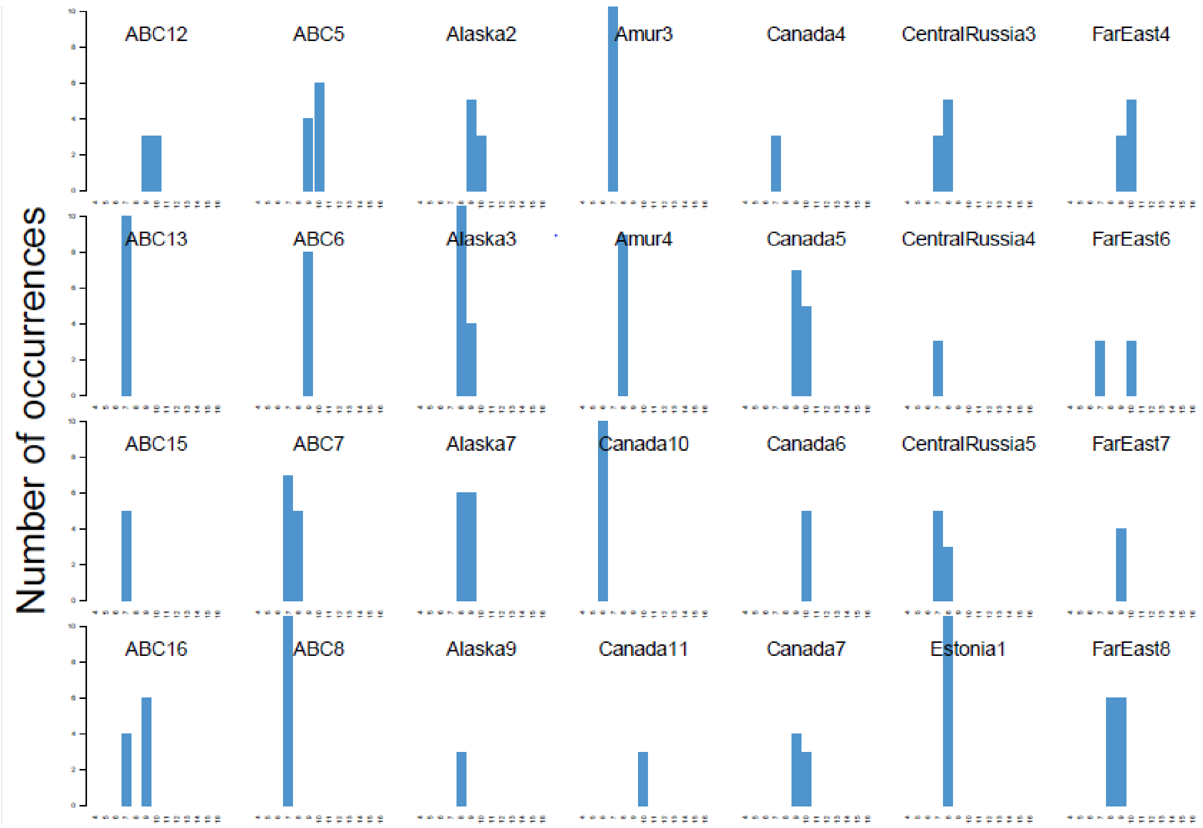

Fig. S13C. Microsatellite (VNTR) genotyping from short read sequencing data. Histograms of observed repeat lengths for one particular microsatellite locus and a random subset of individuals. Individuals with two bars with a minimum height (i.e., read depth) of 3 or higher were assumed to be heterozygous.

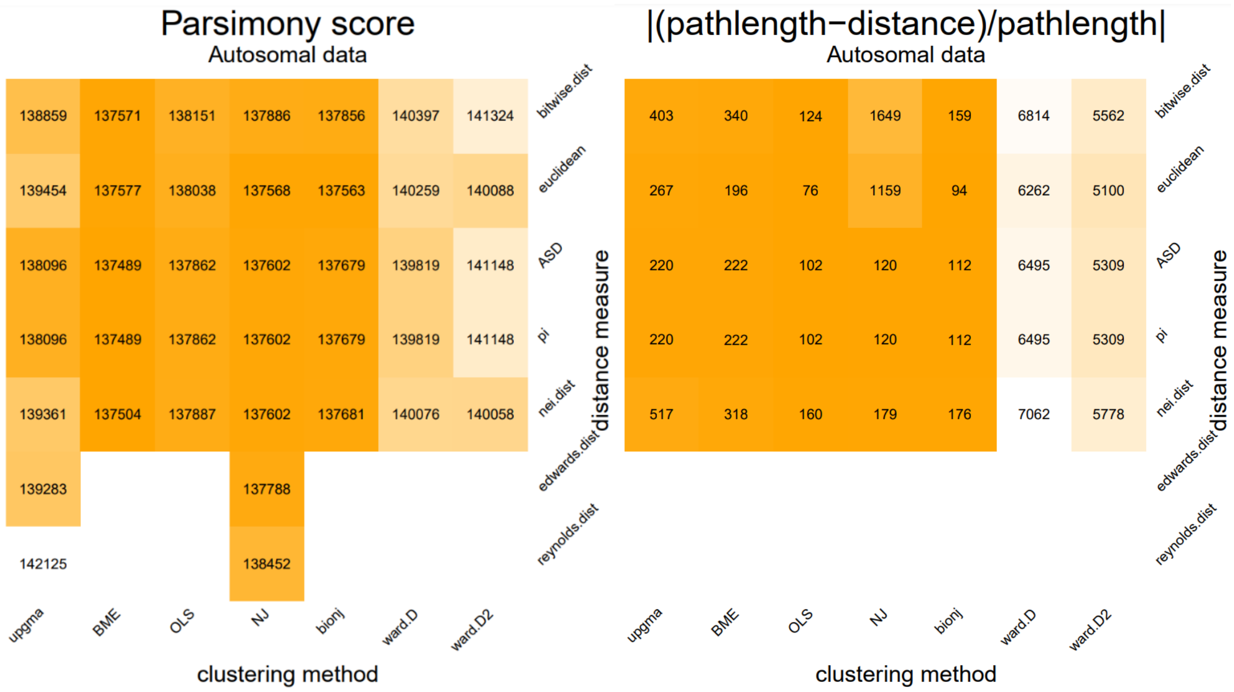

Fig. S14A. Accuracy of hierarchical clustering methods. Heatmaps showing parsimony scores and the difference in pathlength and genetic distance for dendrograms generated using combinations of distance metrics and clustering methods, applied to the autosomal SNP dataset. Lower scores (dark orange) indicate better performance. Based on the path length criterion, dendrograms generated with the Ward method (and to a less extent UPGMA and BME) and Hamming's genetic distance (bitwise.dist) perform poorly for this specific dataset. The best score are obtained with combinations involving Euclidean genetic distance and the OLS and bionj clustering method.

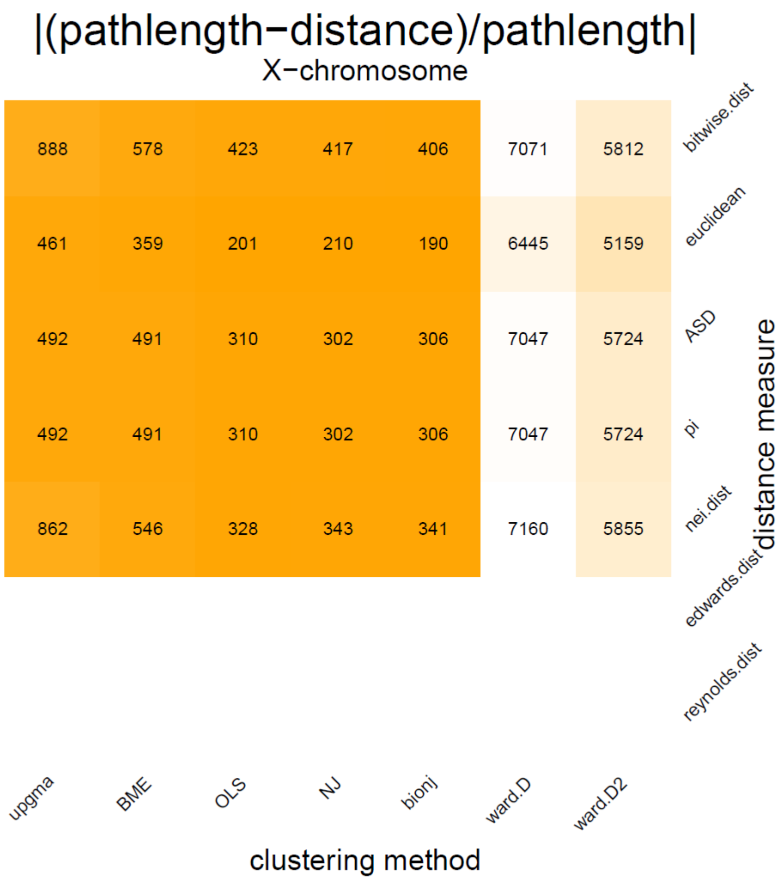

Fig. S14B. Accuracy of hierarchical clustering methods, for X-chromosomal data. Heatmap showing difference in pathlength and genetic distance for dendrograms generated using combinations of distance metrics and clustering methods, applied to the X-chromosomal SNP dataset. Lower scores (dark orange) indicate better performance. Based on the path length criterion, dendrograms generated with the Ward method (and to a less extent UPGMA and BME) and Hamming's genetic distance (bitwise.dist) perform poorly for this specific dataset. The best score is obtained with combinations involving Euclidean genetic distance.

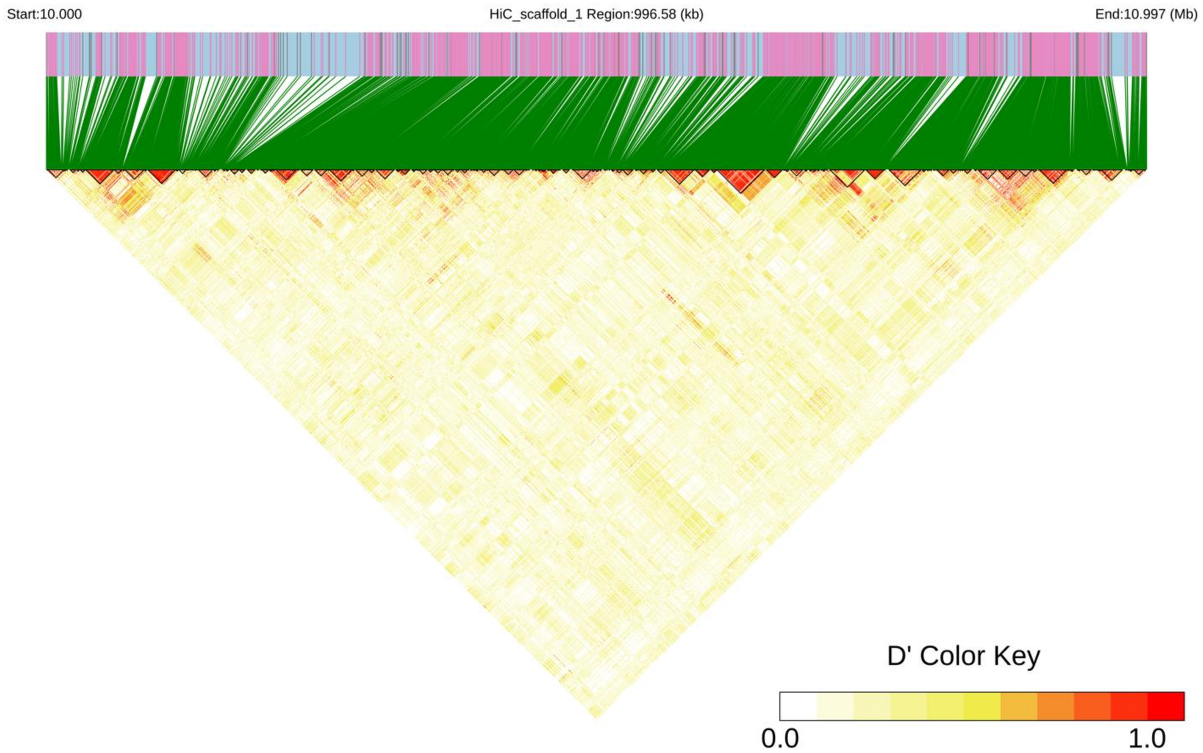

Fig. S15A. Haploblock detection. Linkage disequilibrium estimates for a randomly chosen 1 Mb region for the full dataset of 135 bears, containing 1843 SNPs with a minor allele frequency of 0.2 or higher. The black lines in this overview, generated with the software LDBlockShow, delineate the regions which are identified as haploblocks.

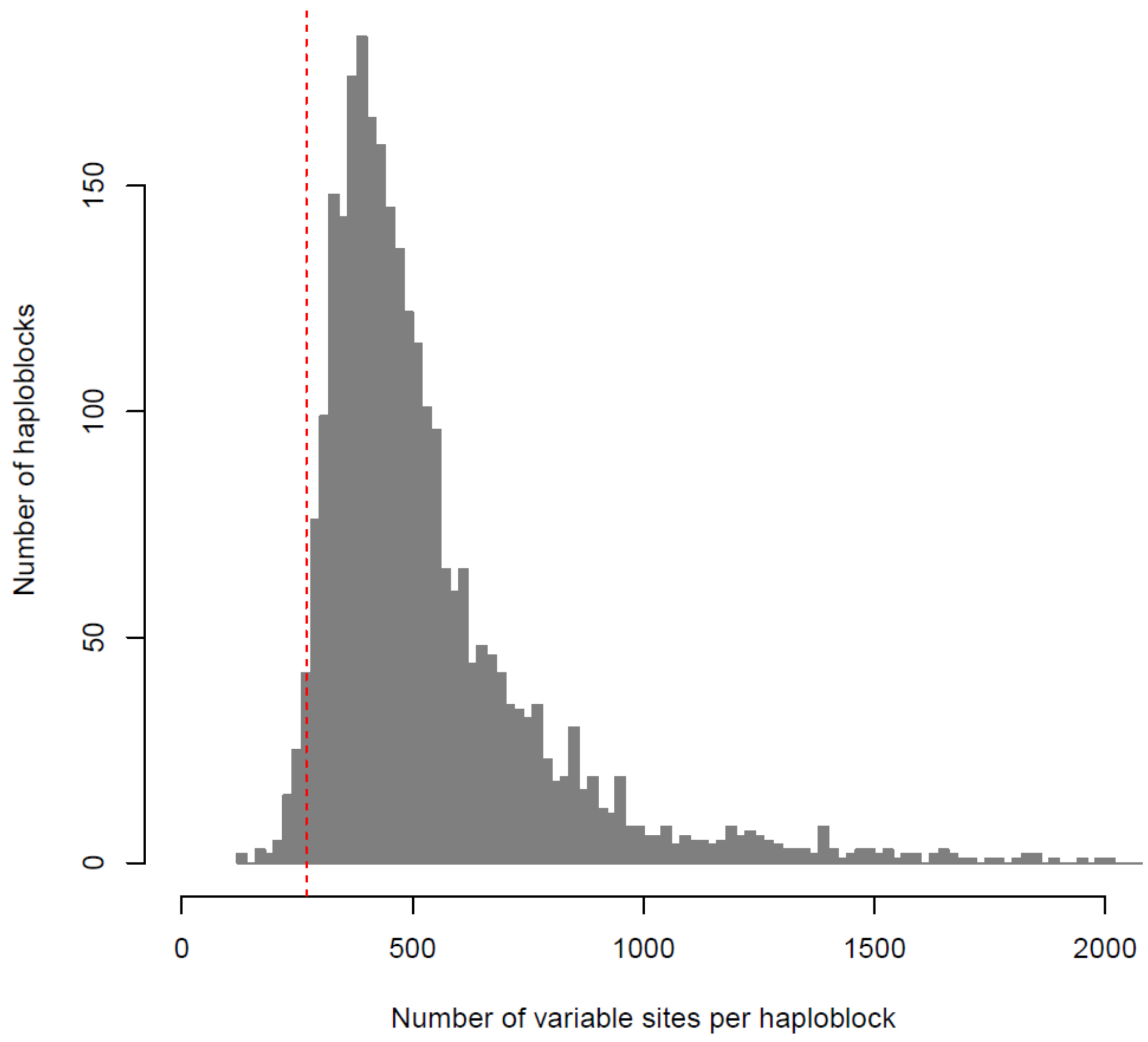

Fig. S15B. Number of variable (i.e., polymorphic) sites per haploblock. Red line indicates number of haplotypes (2x 135 diploid individuals = 270 haplotypes in total).
